# Language structure, attitudes, and learning from ambient exposure: Lexical and phonotactic knowledge of Spanish among non-Spanish-speaking Californians and Texans

**DOI:** 10.1371/journal.pone.0284919

**Published:** 2023-04-27

**Authors:** Simon Todd, Chadi Ben Youssef, Alonso Vásquez-Aguilar

**Affiliations:** Department of Linguistics, University of California, Santa Barbara, Santa Barbara, California, United States of America; Tallinn University: Tallinna Ulikool, ESTONIA

## Abstract

Recent work shows that ambient exposure in everyday situations can yield implicit knowledge of a language that an observer does not speak. We replicate and extend this work in the context of Spanish in California and Texas. In Word Identification and Wellformedness Rating experiments, non-Spanish-speaking Californians and Texans show implicit lexical and phonotactic knowledge of Spanish, which may be affected by both language structure and attitudes. Their knowledge of Spanish appears to be weaker than New Zealanders’ knowledge of Māori established in recent work, consistent with structural differences between Spanish and Māori. Additionally, the strength of a participant’s knowledge increases with the value they place on Spanish and its speakers in their state. These results showcase the power and generality of statistical learning of language in adults, while also highlighting how it cannot be divorced from the structural and attitudinal factors that shape the context in which it occurs.

## 1 Introduction

Our ambient environment is one in which we are often exposed to language. Much of the time, we may not fully engage with such ambient language, attend to it, or even be aware of it; for example, we may treat it as ‘background noise’ in offices, cafés, and public transport hubs, we may overhear someone talking as we pass them in the supermarket or on the street, or we may have a radio or television playing as we do something else. In addition, in multicultural and multilingual settings, this ambient linguistic exposure may not even contain a language that we speak. Nevertheless, there is a great deal of evidence that we learn from such ambient exposure to language, using it to identify statistical patterns and incorporate them into cognitive representations that underpin implicit linguistic knowledge (see Section 2.1).

A lot of the research that has explored language learning from ambient exposure is based in the laboratory, using contrived exposure and, often, artificial languages. While such research has provided useful insights about the basic phenomenon of language learning from ambient exposure, questions remain about how readily those insights translate to everyday situations outside the laboratory. In everyday situations, unlike in the lab, the ambient language may be encountered sporadically over many years, it may have any number of complex structural properties, and it may exist within a social space that sees its use evoke language attitudes in observers. Recent work finds evidence that ambient exposure in everyday situations can yield implicit lexical and phonotactic knowledge of a language that an observer does not explicitly speak (see Section 2.2). However, this work is based on a single language in a single context—the incidental learning of Māori in New Zealand—and thus it is unclear whether its conclusions are generalizable, let alone how they are affected by factors such as language structure and attitudes. In this paper, we replicate this recent work with a different language, in a different context, to determine how generalizable it is, and to extend it.

We explore the incidental learning of Spanish by non-Spanish-speaking Californians and Texans. Spanish in California and Texas shares with Māori in New Zealand the fact that it has a lot of non-speakers who are often ambiently exposed to it (see Section 2.3 for demographic statistics). However, it has distinct structural properties that are implicated in models of ambient language learning, allowing us to explore how learning may relate to language structure (see Section 2.3.1). In addition, it evokes a wide range of strong attitudes in Californians and Texans, allowing us to see how learning may differ with language attitudes (see Section 2.3.2). We use the incidental learning of Spanish by non-Spanish-speaking Californians and Texans to investigate the following research questions:

(RQ1) Do non-Spanish-speaking Californians and Texans show evidence of implicit lexical and phonotactic knowledge of Spanish?
(RQ2) What is the strength and nature of the implicit Spanish knowledge of Californians and Texans? How might the strength and nature of this knowledge relate to the structure of Spanish, as brought to light by meta-comparison with previous results concerning the implicit Māori knowledge of New Zealanders?
(RQ3) How does the implicit knowledge of Spanish held by non-Spanish-speaking Californians and Texans differ with their attitudes toward Spanish and its speakers?

## 2 Background

### 2.1 Statistical learning of language

A great deal of research has established that humans readily identify and track statistical patterns in sensory stimuli, via a domain-general cognitive process known as *statistical learning* (see [1] for a review). Interpreted broadly [2], statistical learning refers to the incidental identification of statistical distributions and recurrent co-occurrences in sensory stimuli, as well as the way that units representing such distributions and co-occurrences are extracted and stored in memory, which together underpin the formation of implicit knowledge through passive exposure. Upon repeated exposure to language, statistical learning builds implicit linguistic knowledge, such as knowledge of phonotactic constraints and probabilities [3–7], knowledge of the cues to word boundaries in continuous speech [8–11], knowledge of what is and is not an attested word [12–17], and knowledge of form-meaning associations [18–20]. This statistical learning of language is robust throughout the lifespan [21]; collectively, the studies cited above provide evidence for it in infants, children, and adults of all ages. Replication studies and meta-analyses [22–24] show that it is also robust across various linguistic structures and experimental tasks; however, mediators of its effects—such as influences of frequency of exposure and stimulus sub-structure—do not consistently replicate, perhaps because they are typically investigated through the learning of highly constrained artificial languages in the laboratory [24].

The building of implicit linguistic knowledge through statistical learning is both passive and powerful. It does not require the learner to understand the language they are exposed to, or even to be actively paying attention to it continuously. For example, [13] asked both children and adults to create computer illustrations while a simple artificial language played in the background, then found in a surprise recognition test that both groups of participants were able to differentiate words of the language (which they had heard) from nonwords (which they had not heard). Furthermore, the knowledge that a learner gains through statistical learning over passive exposure to language can last a long time. For example, [10] exposed young adults to ten hours of speech from an artificial language over ten days, and found that they were still able to distinguish high-frequency words from length-matched non-words with near-perfect accuracy in a surprise test three years later.

Because statistical learning can yield long-lasting implicit linguistic knowledge through passive listening, it has been appealed to as a means through which infants begin the process of language acquisition [25, 26]. In particular, theories of language acquisition propose that infants identify and extract statistically recurrent sequences of sounds—which we refer to as *morphs*—from their linguistic input and store them in a *proto-lexicon* (see [27] for a review). The proto-lexicon is a receptive precursor to the adult mental lexicon; it contains the forms (but not necessarily detailed meanings) of many morphs, and the morphs it contains may correspond to real words, part-words, or nonwords, as long as they are statistically recurrent in the ambient language [16]. It thus endows a blurry form of implicit lexical knowledge, and generalizing over the patterns contained within it yields implicit knowledge of gradient phonotactics [4, 28].

The formation of phonotactic knowledge is often thought to proceed in parallel with the construction of a proto-lexicon, with phonotactic knowledge helping to segment the speech stream and discover new morphs [29], and the discovery of new morphs helping to refine phonotactic knowledge [30]. While it has been argued that phonotactic knowledge can arise in the absence of a proto-lexicon [31], we assume here that the presence of phonotacic knowledge *alongside* implicit lexical knowledge implies the existence of a proto-lexicon. In this case, we assume for modeling purposes that the phonotactic knowledge derives unidirectionally from the proto-lexicon, in the same way as native-language phonotactic knowledge in adults is assumed to derive from the lexicon [32, 33].

### 2.2 A naturally-occurring adult proto-lexicon

It is easy to accept that a proto-lexicon may be constructed by infants during first language acquisition, as well as by adults during intentional second language acquisition, as both processes can lead to explicit linguistic knowledge in the form of a fully-fledged mental lexicon. However, it is not so easy to determine whether a proto-lexicon may be constructed by adults during everyday situations in which they are regularly exposed to a language they do not speak, with no express intention of acquiring it. There is little research investigating the extent to which adults have implicit knowledge of languages they are surrounded by but don’t speak. Furthermore, it is unclear whether it is appropriate to generalize existing research on statistical learning among adults to such situations, because it typically explores learning from artificial languages [7, 10, 13, 17] or highly constrained snippets of natural languages [6, 15], presented in laboratory settings (see also [26] for a discussion of related ecological limitations in research on infant statistical learning of language).

In recent work, [34] found evidence that adult construction of a proto-lexicon through statistical learning is not limited to artificial or highly constrained languages in the laboratory, but also occurs with sustained ambient exposure to a natural language in the world. They conducted two experiments with New Zealanders who are commonly surrounded by Māori, the Indigenous language, but do not speak it. In a Word Identification experiment, participants were able to distinguish real words from word-like (phonotactically-matched) nonwords, demonstrating implicit lexical knowledge. In a Wellformedness Rating experiment, they were able to evaluate how strongly different nonwords conform to probabilistic phonotactic patterns of the language, demonstrating implicit phonotactic knowledge. A later study by [35] replicated these effects with more tightly-controlled stimuli, and showed a link between them: participants who were better able to distinguish real Māori words from highly Māori-like nonwords in the Word Identification experiment were also more sensitive to underlying phonotactic probabilities in the Wellformedness Rating experiment. This finding underscores the idea that non-Māori-speaking New Zealanders have a Māori proto-lexicon, from which their implicit lexical and phonotactic knowledge jointly derives.

In both of [34]’s experiments, the implicit knowledge of Māori demonstrated by non-Māori-speaking participants appeared to be surprisingly strong. In particular, in the Wellformedness Rating experiment, non-Māori-speaking New Zealanders were as sensitive to underlying phonotactic probabilities as fluent Māori speakers, despite the fact that non-Māori-speaking New Zealanders typically only explicitly know 70–80 Māori words on average [36]. This result suggests that the proto-lexicon from which their knowledge is derived may be surprisingly large. Monte Carlo modeling supported this suggestion: if participants’ knowledge is assumed to stem from a proto-lexicon, that proto-lexicon would need to contain the forms of at least approximately 1,500 morphs in order to best explain the pattern of responses in the Wellformedness Rating experiment. In addition, the Monte Carlo modeling showed that the experimental results could be better explained by a proto-lexicon containing morphs that commonly occur in corpora, rather than a proto-lexicon containing a random collection of morphs from the language, underscoring the idea that the proto-lexicon is built through passive exposure.

Since the results of [34, 35] are, to our knowledge, the only evidence for a naturally-occurring proto-lexicon among adults, further work is needed in other contexts in order to determine their generality. As it stands, it is conceivable that aspects of the results might be attributable to the specific context of Māori in New Zealand, rather than to general principles of statistical learning of language. For example, they could be driven by structural factors, such as the fact that Māori has a small phoneme inventory and simple (C)V syllable structure [37, 38], as well as strictly concatenative morphology [37] that makes extensive use of compounding [38, 39], which together cause a wide range of morphs to recur across words often and thus facilitates statistical learning (see e.g. [40]). They could also be affected by the increasingly positive attitudes that Māori evokes in non-Māori-speakers (see ch. 4 of [41] for a review), given evidence that attitudes affect the ease with which implicit linguistic knowledge is encoded in, and accessed from, memory (see Section 2.3.2 for discussion). To address these open issues, and thereby both test and extend what is known about the adult proto-lexicon, we investigate implicit lexical and phonotactic knowledge of Spanish among non-Spanish-speaking Californians and Texans.

### 2.3 Spanish in California and Texas

Spanish is the largest minority language within the United States. Estimates from the American Community Survey between 2016–2020 [42] indicate that approximately 40.5 million people in the United States speak Spanish at home, accounting for 61.3% of people who speak a language other than English at home. With an additional 15 million who speak Spanish with limited proficiency outside of the home (e.g., as second-language learners; [43]), approximately 18.1% of those in the United States have some (non-trivial) degree of explicit knowledge of Spanish.

California and Texas are the states with the two largest Spanish-speaking populations in the United States, both in absolute terms and relative to state population [42]. In 2020, California was estimated to have nearly 10.5 million people who speak Spanish at home (28.3% of the population of the state), while Texas had nearly 7.7 million (28.8% of the population of the state). This means that Spanish is highly overrepresented in California and Texas relative to the United States as a whole: 44.7% of those in the United States who speak Spanish at home live in California and Texas, despite these states only accounting for 20.7% of the national population. Of course, California and Texas are not homogeneous: some areas have high concentrations of Spanish speakers, while others have very low concentrations. We assume that most of the participants in our experiments will be from large metropolitan areas, where there are particularly high concentrations of Spanish speakers [44], because we recruit them via web-based platforms whose users tend to be distributed geographically in proportion to population [45]. As a result, the non-Spanish-speaking Californians and Texans we study can be expected to have much more exposure to Spanish, on average, than Americans in general, and many may have quite high degrees of exposure.

California and Texas are also the states with the two largest Hispanic/Latinx populations in absolute terms and the second- and third-largest Hispanic/Latinx populations relative to state population. In the 2020 Decennial Census [46], there were 15.6 million people in California (39.4% of the population of the state) and 11.4 million in Texas (39.3% of the population of the state) who identified as Hispanic/Latinx. Furthermore, there is a strong correlation between being Hispanic/Latinx and speaking Spanish in these states, with 77% of Hispanics/Latinx in California and 78.4% in Texas speaking Spanish at home [47]. Californians and Texans therefore may be expected to have high degrees of exposure to Hispanic/Latinx cultures in addition to Spanish language, and may be expected to make associations between the two both cognitively and attitudinally.

As a result of the speaker statistics discussed here, Spanish in California and Texas offers an ideal case with which to assess the replicability of the results of [34, 35] discussed in Section 2.2. There are a lot of people in California and Texas who do not speak Spanish, but may be exposed to it a lot. Do such non-Spanish-speaking Californians and Texans gain implicit lexical and phonotactic knowledge of Spanish as a result of their exposure, like non-Māori-speaking New Zealanders do of Māori? At the same time, Spanish in California and Texas is different from Māori in New Zealand in crucial ways, allowing us to examine the generality of the previous results and thus extend what is known about the construction of an adult proto-lexicon. Two main areas of difference concern the structure of the language (Section 2.3.1) and the attitudes that non-speakers have toward it and its speakers (Section 2.3.2).

#### 2.3.1 Structural elements of Spanish

One important aspect of Spanish for the present study concerns its structure. Like Māori, Spanish has a (relatively) transparent mapping between orthographic and phonological forms. This transparent mapping suggests that implicit knowledge gained through exposure to written and spoken forms may converge in cognitive representations, and allows written words to activate phonotactic knowledge in a straightforward way (see e.g. [48] for a review). However, compared to Māori, Spanish has a larger phoneme inventory—24 phonemes [49], compared to 15—alongside more complex syllable structure that permits codas and certain consonant clusters [50]. Spanish also has notable morphological differences to Māori, such a lower use of compounding compared to (inflectional or derivational) affixation [51–53]; for example, in a survey of 10 Spanish dictionaries spanning approximately 1,300 years, [54] counts fewer than 3,600 compounds in total. In addition, the presence of a number of morphophonological alternations [55] means that Spanish morphology is not as concatenative as Māori morphology: roots and stems can take on a variety of forms depending on the affix(es) with which they appear.

Following the principles of learning morphological segmentation through passive exposure embodied in computational models [40], these structural differences between Spanish and Māori are likely to give rise to differences in the ability to construct a proto-lexicon and gain implicit phonotactic and lexical knowledge. On the one hand, the differences in phonological inventory size and syllable structure mean that estimating fine-grained phonotactic probabilities requires access to more word types in Spanish than it does in Māori, which may limit the amount of implicit phonotactic knowledge that non-Spanish-speakers can gain through passive exposure. But on the other hand, they also mean that phonotactic cues may be expected to be more consistent in Spanish than in Māori, which may provide advantages for the segmentation of speech that underlies the construction of a proto-lexicon (Section 2.2). Similarly, the differences in morphological structure mean that more distinct morphs are likely to occur in Spanish speech than in Māori, but fewer of them are likely to recur with statistical regularity, which may make it difficult to construct a large proto-lexicon of robust representations.

#### 2.3.2 Language attitudes toward Spanish and its speakers

A second important aspect of Spanish in California and Texas concerns the attitudes that non-speakers may have toward it. Language attitudes can be understood as a reflection of language ideologies; that is, as “socially and culturally embedded metalinguistic conceptualizations of language and its forms of usage” [56] (p. 241). In addition to being based on conceptualizations of a language or variety itself, language attitudes are based on conceptualizations of the broader group(s) of users of that language or variety; as [57] (p. 638) states, “although the immediate object may be the language, the real target is the minority”. Thus, exposure to a given language or variety can cue attitudes that implicitly reflect larger social structures and biases, either negative or positive.

The United States has a long history of tacitly assuming and socially enforcing monocultural and monolingual ideological norms [58]. As a result, there are salient negative attitudes associated with languages other than English, and their speakers, due to the cultural (oft-called ‘racial’) threat that they are perceived to bring to these norms [59–63]. These negative attitudes are correlated with nationalist, anti-immigration, and English-only stances, and are often targeted toward Spanish and its speakers, as the largest minority language group in the country (Section 2.3). For example, [60] found that, among (non-Hispanic/Latinx) White Americans, those who have negative attitudes toward speakers of languages other than English are more likely to support reducing immigration and enforcing deportation policies, and that such negative attitudes and their consequences are heightened after brief exposure to Spanish. Yet the picture is not all bad: [60] also found that the aforementioned negative attitudes and their consequences were weaker among White Americans who have a recent immigrant among close friends and family, and [63] found that (non-Hispanic/Latinx) White and Black Americans who speak a language other than English held positive attitudes toward policies on bilingual education. These two contrasting pictures reflect different dimensions of language attitudes: on the one hand, a language and its speakers may be met with negative attitudes because they are perceived to undermine the stability of large-scale ideological structures of societal monoculturalism; but on the other hand, they may be met with positive attitudes based on their perceived value at a personal or local level.

For both negative and positive language attitudes toward Spanish and its speakers in the United States, the literature indicates that key roles are played by population size and degree of exposure. When White Americans live in areas with large and growing Hispanic/Latinx populations and are exposed to Spanish often, those that do not have many close social interactions and personal relationships with Hispanics/Latinx tend to develop negative attitudes, while those that do have many social interactions and personal relationships tend to develop positive attitudes [59–62, 64, 65]. It follows that Californians and Texans, living in the states with the largest Spanish-speaking and Hispanic/Latinx populations (Section 2.3), are likely to exhibit a range of attitudes toward Spanish and its speakers.

Much work has explored the attitudes that Californians and Texans hold toward Spanish and its speakers, focusing in particular on negative attitudes. In California, a history of anti-Spanish attitudes can be seen in English-only language education policies [66]. Relatedly, [64] report stronger negative attitudes toward Hispanics/Latinx in California than in other states. Negative attitudes are also evoked in Californian listeners by brief exposure to mildly Spanish-accented English: in a verbal-guise experiment, [67] found that teenage listeners in Santa Ynez rated Spanish-accented speakers lower than (White Californian) Anglo-accented speakers across a range of personality traits. In Texas, research has highlighted equally strong negative attitudes toward Spanish and its speakers. [65] find that White Texans have negative attitudes toward Hispanics/Latinx when they do not socially interact with them regularly, particularly in areas with large Hispanic/Latinx populations. Correspondingly, [61] find relatively low support for bilingual education policy in Texas, especially in areas with large and growing Hispanic/Latinx populations. Finally, negative attitudes are also evoked in Texans by Mexican Spanish-accented English: [68] found that listeners in El Paso would be less likely to hire a Mexican Spanish-accented speaker as a local weather presenter than a Standard American-accented speaker, even though many of them ascribe value to Spanish-English bilingualism.

The attitudinal situation that the literature describes for California and Texas—with a wide range of attitudes toward Spanish and its speakers, including some very negative ones—is quite different to the situation described for New Zealand. In contemporary New Zealand culture, attitudes toward Māori and its speakers are generally positive [69, 70] (see Chapter 4 of [41] for more detailed discussion). The data collected for the present study (Section 6) confirm that the range of attitudes toward Spanish among Californians and Texans in our experiments is much wider than the range of attitudes toward Māori among New Zealanders in [35]’s experiments. [35] asked participants to rate how strongly they agreed with the idea that some Māori education should be compulsory in New Zealand schools. Only 8 of 187 participants (4.3%) disagreed, while 12 (6.4%) were neutral and 167 (89.3%) agreed. In our experiments, we asked the same question about compulsory Spanish education in California and Texas schools, and found a much broader range of responses: 25 of 79 participants (31.6%) disagreed, 22 (27.8%) were neutral, and 32 (40.5%) agreed.

We expect Californians and Texans’ attitudes toward Spanish and its speakers to be relevant to the construction of a Spanish proto-lexicon based on evidence that attitudes affect basic aspects of linguistic cognition. For example, a host of research under the umbrella of Communication Accommodation Theory [71] has shown that listeners are more likely to imitate phonetic features of speakers they feel positively inclined toward than of speakers they feel negatively inclined toward [72, 73]. Since phonetic imitation is typically assumed to occur below the level of conscious awareness, through the activation and/or updating of cognitive representations of language, these results suggest that attitudes mediate linguistic perception and memory. Further support for these suggestions is offered by direct experimental investigations of socially-based asymmetries in perception and memory of speech. For example, [74] found that Australian listeners with negative attitudes toward Asians are worse at perceptually categorizing Vietnamese English-accented vowels than listeners with positive attitudes, and [75] found that American listeners have weaker memory for words spoken by a New York City English speaker (with a ‘low-prestige’ accent that is typically negatively evaluated) than for words spoken by a General American English or British English speaker (with accents that are typically positively evaluated). Since the construction of a proto-lexicon requires perceiving language input faithfully and storing it in memory robustly, we expect that any limitations for linguistic perception and memory connected to negative attitudes toward Spanish and its speakers will also translate to limitations in Spanish proto-lexicon construction.

## 3 Methods

To explore the implicit knowledge of Spanish held by non-Spanish-speaking Californians and Texans, we ran two web-based experiments, following [34]. Experiment 1 is a Word Identification experiment, which tests the extent to which participants are able to discriminate between real Spanish words and highly word-like nonwords. Experiment 2 is a Wellformedness Rating experiment, which tests the extent to which participants draw upon phonotactic probability in evaluating the degree to which a Spanish nonword is word-like.

The experiments were reviewed and approved by the Human Subjects Committee of the University of California, Santa Barbara. Participants received information about the study when previewing an experiment and again on the first page upon accepting the experiment, and indicated consent by clicking a button.

### 3.1 Materials

Experiment 1 used a pool of 510 real Spanish words, each paired with a phonotactically legal nonword that was matched for length (both phonemic and orthographic), phonotactic probability, and presence of stress-marking character accents. Experiment 2 used a pool of all 510 nonwords created for Experiment 1. For both experiments, orthographic forms were converted to phonological forms for stimulus selection and analysis; we originally created our own rewrite rules for stimulus selection, but switched to using the rewrite rules of eSpeak NG (a publicly available text-to-speech engine) for analysis, to facilitate greater accuracy and reproducibility. Phonotactic probabilities were calculated on the basis of these phonological forms using a Witten-Bell-smoothed trigram language model over phonemes, and were converted to length-normalized *phonotactic scores* that represent the average conditional log-probability of phonemes in the stimulus (base 10). The phonotactic models were trained on unique word types in the SUBTLEX-ESP lexical database of Spanish film subtitles [76]. Full details of stimuli and of phonotactic scoring can be found in the S1 File Detailed Materials and Methods Supplement, Sections 2 and 4, respectively; here, we give an overview.

We obtained the real Spanish words from the intersection of the SPALEX Database [77] with the database utilized by Wuggy [78], so that they would be “base forms” (i.e. words that are not proper nouns, inflected forms, or compounds) for which highly similar nonwords could be easily generated. We assigned words to three bins based on their frequency as recorded in in the SUBTLEX-ESP lexical database [76] (low-frequency: fewer than 10 occurrences per million; mid-frequency: 10–100 occurrences per million; high-frequency: more than 100 occurrences per million). We then calculated a phonotactic score for each word, representing its length-normalized phonotactic probability, and we selected words of phoneme length 5–8 that spanned a wide range of phonotactic scores, in such a way that distribution of phonotactic scores was as similar as possible across lengths and frequency bins.

For the nonword stimuli, we used Wuggy [78] to generate 10 candidate nonwords for each word. Each candidate nonword was required to be the same phonemic and orthographic length as the original word, and to contain an accented vowel character (marking non-default stress assignment) wherever the original word did. We chose the best candidate nonword in each case as the one that was closest to the original word in phonotactic score, provided it was not judged by a native speaker of Spanish (the third author) as markedly nonword-like or potentially morphologically complex. For Experiment 1, the chosen nonword was assigned to the same frequency bin as its matched real word.

Within each experiment, 240 stimuli were randomly sampled from the pool per experimental session (participant). In Experiment 1, sampling was conducted over word-nonword pairs, to ensure that the properties of words and nonwords were matched within an experimental session. For each session of Experiment 1, 240 items in 120 word-nonword pairs were sampled in proportion to the sizes of groups defined by length and frequency bin. In Experiment 2, sampling was conducted over nonwords only. For each session of Experiment 2, 240 nonwords were sampled in proportion to the sizes of groups defined by length.

Both experiments presented stimuli in written form, rather than in audio form, in order to ensure that participants would perceive them as intended and would not be influenced by phonetic characteristics of a talker’s rendition (e.g., how confident or fluent they sounded). While the use of written stimuli protects the investigation from undue influence of such perceptual noise, is not without limitations: it necessitates the assumption that participants can map between orthographic and phonological representations of Spanish, and that phonological (phonotactic) knowledge obtained (at least in part) by passive listening can be accessed through reading. Nevertheless, both of these assumptions are reasonable, given the tight and transparent mapping between orthography and phonology in Spanish (as described in Section 2.3.1), the high degree of Spanish that participants are likely to encounter in California and Texas (e.g., in place names, official forms and signage, and ambient speech), and previous work on the activation of phonological knowledge by orthographic forms [48]. It is possible that non-Spanish-speaking Californians and Texans have incomplete or inaccurate knowledge of the orthography-phonology mapping in Spanish, and/or that they are influenced by both orthotactic and phonotactic properties of the written stimuli; however, full investigation of these possibilities is beyond the scope of this paper. We control for the most salient visual property of Spanish orthography—namely, the presence of accented characters 〈á, é, í, ó, ú, ñ〉 that are not found in English—in the nonword stimulus creation process described above, as well as in the statistical analyses of experimental results.

After running the experiments, we realized that the pool of stimuli included a small number of items (words and nonwords) that were orthographically identical to English words. All responses to such stimuli were excluded from analysis, under the assumption that participants’ perception of them as English words might affect their responses. We did not exclude responses to common Spanish words that have been borrowed into English, as the orthographic and phonotactic properties of such words allow them to be identified as Spanish in origin, in spite of their use in English-speaking contexts. We also realized that switching the method of orthographic-to-phonological form conversion between stimulus selection and analysis reduced the closeness of phonotactic matches between a small set of words and non-words. Accordingly, we excluded from analysis all responses to stimuli from word/non-word pairs that were closely matched for phonotactic score in stimulus selection, based on the phonological forms derived from our own rewrite rules, but were no longer closely matched in analysis, based on the phonological forms derived from eSpeak NG. For a full description of the stimulus exclusions, see the S1 File Detailed Materials and Methods Supplement, Sections 2.3–2.4.

### 3.2 Procedures

Each experiment ran in a web browser, on the participant’s own computer. Prior to an experiment starting, the participant completed screening questions that ensured their eligibility (described in more detail in Section 3.3). The participant was informed if their answers to the screening questions indicated that they were ineligible, in which case the experiment would not launch. If the participant was deemed eligible, they read instructions for the experimental task, and carried out the task as described below (for more details about the tasks and instructions, see the S1 File Detailed Materials and Methods Supplement, Section 3). Finally, they completed a post-task questionnaire, which asked for demographic information, as well as ratings of their exposure to and attitudes toward Spanish (see the S1 File Detailed Materials and Methods Supplement, Section 6.2). For each experiment, the entire procedure was designed to be completed within 20 minutes.

*Experiment 1: Word Identification.* In Experiment 1, the participant was presented with a stimulus (orthographic representation of a word or nonword) and an array of 5 radio buttons, where the edges of the button array were labeled “Confident that this is NOT a Spanish word” and “Confident that this IS a Spanish word”. The participant was instructed to click a button on the scale, indicating their degree of confidence that the stimulus was a real Spanish word.

*Experiment 2: Wellformedness rating.* Experiment 2 proceeded in exactly the same way as Experiment 1, except the stimuli were all nonwords, and the labels at the edges of the button array were “HARDLY Spanish-like” and “VERY Spanish-like”. The participant was instructed to click a button indicating how Spanish-like they perceived the nonword to be.

Each experimental session consisted of 240 critical trials, as described above, presented in random order. In addition, each session included 6 attention check trials, distributed evenly throughout the trials. In an attention check trial, an instruction to click on a particular button was shown in place of a stimulus, and the labels at the edges of the button array were hidden. If the participant failed more than one attention check trial, they were informed that they were not completing the experiment correctly, and were prevented from continuing. The six attention check trials targeted the leftmost, middle, and rightmost buttons twice each, in random order.

### 3.3 Participants

For each experiment, we recruited 100 participants via Amazon Mechanical Turk. All participants were required to have accounts registered in either California or Texas, and no participant was permitted to complete an experiment more than once from a single account. Before beginning the experiment, participants completed a pre-screening questionnaire to self-report that they were eligible for the experiment, namely: that they were American English speakers; that they grew up and currently lived in California or Texas, and had not spent more than a year outside this state; that they had never taken a college-level course in Linguistics; and that they could not hold a basic conversation in Spanish (for the full pre-screening questionnaire, see the S1 File Detailed Materials and Methods Supplement, Section 6.1). As long as a participant self-reported being eligible for the experiment—as indicated by the pre-screening questionnaire—and failed no more than one of the attention checks in the course of completing the experiment, they were paid $5 in compensation for their time. We did not screen out participants on the basis of formal education in Spanish, as some students may have been required to take Spanish prior to college but may not have learned (or retained) much; however, we did exclude from analysis participants who had taken a college-level Spanish course, on the grounds that they did so by choice and are likely to have covered a substantial amount of material.

When inspecting the data prior to analysis, we removed a number of participants who appeared not to have completed the experiment in good faith, based on indications that they completed it multiple times under different accounts, used automated methods, or were not fully attending throughout the experiment and questionnaire (despite having passed the attention checks). In total, we removed 13 participants from Experiment 1 and 9 participants from Experiment 2. In addition, we excluded from the analysis a large number of participants from each experiment who we believe to have completed the experiment in good faith, but whose answers in the post-task questionnaire cast doubt on their eligibility, who skipped multiple trials due to technical errors, or who gave largely unvarying responses. Most of these exclusions were of participants who indicated in the post-task questionnaire that they had taken a college-level Spanish course (see above), or that they could speak and/or understand Spanish fairly well. The number of participants who were excluded from the analysis is much larger than we had originally anticipated, creating potential limitations for our ability to detect fine-grained effects. These limitations have particular implications for our investigation of individual differences among participants in Section 6, but are not likely to have affected our identification of major population-level effects elsewhere. For full details of participant removals and exclusions, see the S1 File Detailed Materials and Methods Supplement, Sections 1.1–1.2.

After all removals and exclusions, the analysis of Experiment 1 is based on data from 40 participants, and the analysis of Experiment 2 is based on data from 39 participants. Participants in both experiments are evenly split between Californians and Texans, and all indicate low levels of Spanish proficiency, alongside middling degrees of exposure to Spanish and a range of attitudes toward Spanish. For a full description of the demographics of participants, see the S2 File Detailed Analysis and Results Supplement, Sections 2.2 (Experiment 1) and 3.2 (Experiment 2).

## 4 Evidence for a proto-lexicon

Our initial analysis of the experiments looks for evidence of both lexical and phonotactic knowledge. Following [34], we interpret such evidence as indicative of a Spanish proto-lexicon; that is, of implicit knowledge of a large number of Spanish wordforms.

The analyses of both experiments utilize mixed-effects (logit) ordinal regression, as implemented in the ordinal package [79] in *R* [80]. We use a statistical significance threshold of *α* = 0.05 and follow the model-fitting procedure used by [34], to ensure maximal comparability with earlier results. For details of the model-fitting procedure, see the S2 File Detailed Analysis and Results Supplement, Section 2.4. All data and code used in the analyses are available in an OSF repository at https://osf.io/au62c/.

In the following subsections, we highlight the significant effects revealed by the ordinal regression analysis, reporting in each case the coefficient, *z* value, 95% confidence interval, and *p* value returned by the model. Note that interpretation of the effects on ratings embodied by coefficients in ordinal regression models requires comparison with the decision thresholds estimated by the model. These decision thresholds are contained in full model summary tables, which are reported in the S2 File Detailed Analysis and Results Supplement, Sections 2.4.1 and 3.4.1.

### 4.1 Word identification

In Experiment 1, participants rated word and nonword stimuli for their degree of confidence that they were real Spanish words. In a similar experiment with Māori, [34] found that participants rated real words higher than nonwords, especially for high-frequency words, and that participants’ ratings increased with the phonotactic score of the stimuli. If participants in our experiment have a Spanish proto-lexicon, we expect their ratings here to follow the same patterns.

**Fig 1:**
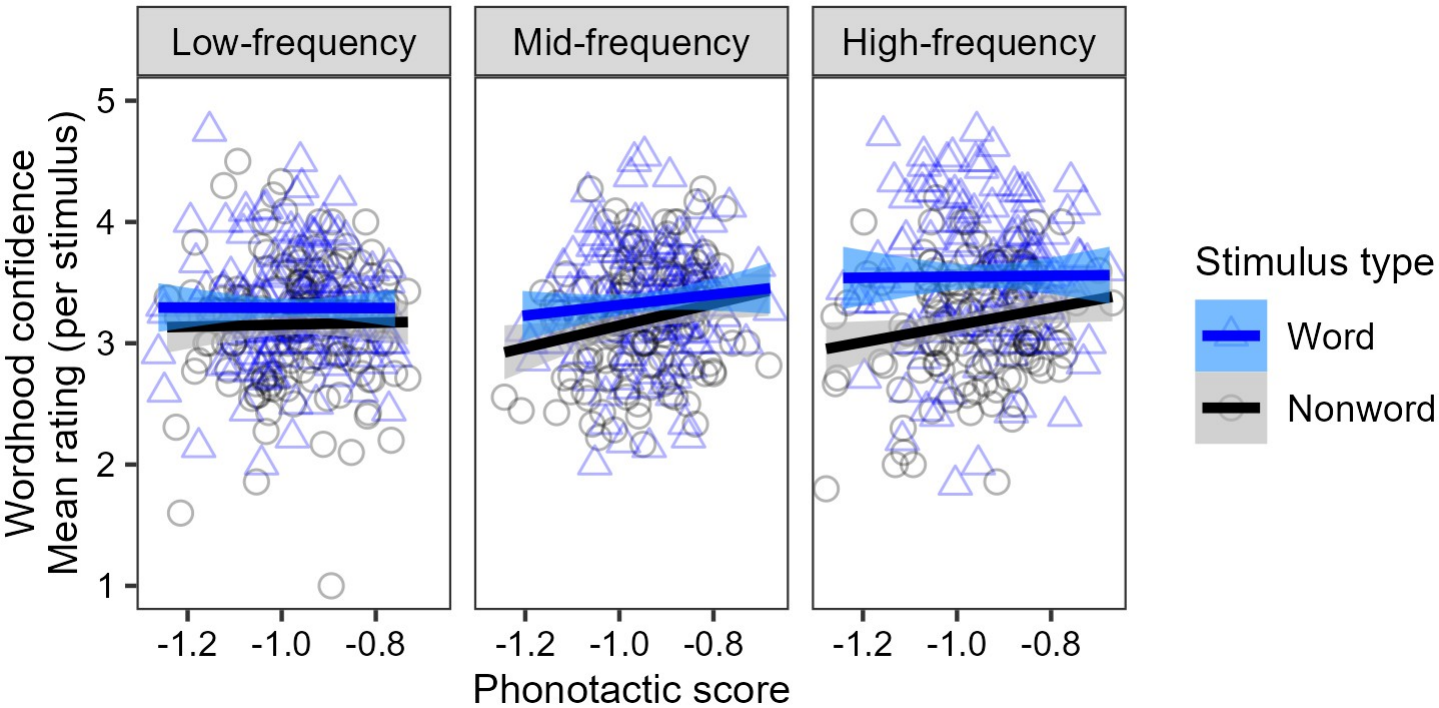
Summary of raw results from Experiment 1, showing participants’ mean wordhood confidence ratings for each stimulus per frequency bin, and their relation to phonotactic score. Points represent mean ratings for each real word (blue triangles) and nonword (black circles) within each bin, and straight lines show correlations with phonotactic scores. Across all bins, stimuli with higher phonotactic scores receive higher ratings, and real words receive higher ratings than nonwords, with this difference becoming particularly pronounced in the high-frequency bin.

Fig 1 shows a summary of the raw results from Experiment 1. On average, participants give higher wordhood confidence ratings to real Spanish words than to phonotactically-matched nonwords. This difference is strongest in the high-frequency bin, but appears to hold in general across all bins. In addition, across all frequency bins and for both words and nonwords, stimuli with higher phonotactic scores are rated more likely to be real Spanish words than those with lower phonotactic scores.

The mixed-effects ordinal regression analysis confirms these patterns. There is a significant main effect of the stimulus type (word or nonword), such that participants rate real words higher than nonwords (*β* = 0.459, *z* = 4.426, 95% CI [0.256, 0.663], *p* < 0.001). This effect of stimulus type interacts significantly with frequency bin, such that the difference is stronger in the high-frequency bin than in the low- and mid-frequency bins combined (*β* = 0.542, *z* = 3.489, 95% CI [0.238, 0.847], *p* < 0.001). Nevertheless, a post-hoc comparison of estimated marginal means confirms that the effect of stimulus type is significant in each of the frequency bins, and not just restricted to the high-frequency bin (high: EMM = 0.821, *z* = 4.517, 95% CI [0.465, 1.177], *p* < 0.001; mid: EMM = 0.301, *z* = 2.410, 95% CI [0.056, 0.547], *p* = 0.016; low: EMM = 0.256, *z* = 2.403, 95% CI [0.047, 0.465], *p* = 0.016). In addition, there is a significant main effect of phonotactic score (*β* = 1.083, *z* = 3.050, 95% CI [0.387, 1.778], *p* = 0.002), which does not interact with stimulus type or frequency bin.

We interpret these results as indicating that participants possess both lexical and phonotactic knowledge of Spanish. We take this knowledge to derive from a proto-lexicon—an implicit memory store of a large number of wordforms, built automatically through passive exposure—for four main reasons. First, though participants’ ratings of words reflect lexical knowledge by being higher than their ratings of nonwords, the fact that they are nevertheless between 3 and 4 on the scale on average indicates that participants are generally not confident in their lexical knowledge, which suggests that this knowledge is implicit rather than explicit. Second, the fact that participants give the highest ratings to high-frequency words suggests that the implicit knowledge is built through exposure, as they are likely to have been exposed to such words more often than they have to lower-frequency words. Third, the fact that participants show evidence of lexical knowledge even for low-frequency words suggests that their acquisition of knowledge is guided by automatic processes during passive exposure, since they are likely not exposed to such words often enough to benefit from active or intentional learning. And fourth, the fact that participants are sensitive to fine-grained phonotactic probabilities in their rating of both words and nonwords suggests that their knowledge is composed in such a way as to permit them to reference to a large set of Spanish words, in order to establish what is typical of Spanish words in general.

The patterns that exist in these results replicate the main findings of the word identification experiment of [34]. However, there appear to be differences in the strength of the patterns seen in the two studies. In particular, [34] found a very strong effect of phonotactic score, whereby increasing from −1.2 to −0.8 on the phonotactic score scale (i.e., increasing phonotactic probability by a factor of 2.5, from an average conditional probability of approximately 1/16 per phoneme to approximately 1/6) was associated with an increase of approximately 0.5 points on the wordhood confidence rating scale, on average. By comparison, we found an effect that was numerically much weaker, with the same increase on the phonotactic score scale being associated with an increase of approximately 0.25 points on the wordhood confidence rating scale, on average. While it is not appropriate to conduct a statistical comparison of these effect sizes, we would not be surprised if the effect of phonotactic score in Māori is indeed statistically significantly stronger than the effect in Spanish, since the structural differences between the two languages (Section 2.3.1) imply that phonotactic probabilities in Māori can be estimated firmly from a smaller set of lexical items. Of course, there are many other factors that may differ between Māori in New Zealand and Spanish in the United States, such as attitudes (see Section 6) and degrees of exposure, and it is possible that these factors may provide additional or alternative sources to the apparent difference in effect sizes across languages.

Finally, we note that the mixed-effects ordinal regression analysis also reveals a significant main effect of the presence of non-English characters 〈á, é, í, ó, ú, ñ〉, such that participants are likely to give a stimulus a higher rating if it contains any non-English character(s) (*β* = 1.122, *z* = 4.264, 95% CI [0.606, 1.638], *p* < 0.001). This effect does not interact with any of the other effects described previously; thus, we do not interpret it as particularly relevant to implicit lexical or phonotactic knowledge, but rather as a simple indicator of the visual salience of such characters for English-speaking participants.

### 4.2 Wellformedness ratings

In Experiment 2, participants rated nonword stimuli for how Spanish-like they were. In a similar experiment with Māori, [34] found that participants gave higher ratings to nonwords with higher phonotactic scores, indicating phonotactic knowledge that is assumed to derive from a proto-lexicon. If participants in our experiment have a Spanish proto-lexicon, we expect their ratings here to follow the same pattern.

**Fig 2:**
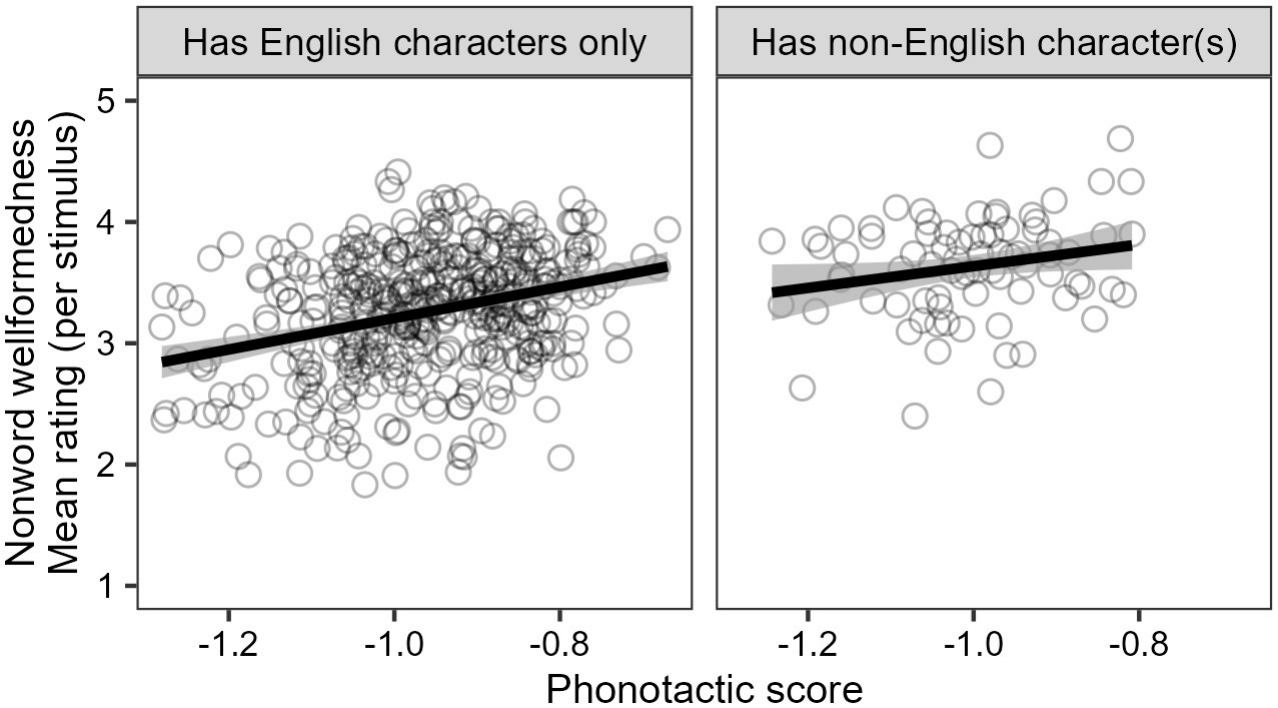
Summary of raw results from Experiment 2, showing participants’ mean wellformedness ratings for each nonword stimulus and their relation to phonotactic score, split based on whether or not the stimulus contains a non-English accented character. Points represent mean ratings for each nonword, and straight lines show correlations with phonotactic scores. In general, ratings increase with phonotactic score, and nonwords containing a non-English character are rated higher than those without any such characters.

Fig 2 **shows a summary of the raw results from Experiment 2**. On average, participants’ ratings do indeed appear to increase with phonotactic score. This pattern seems to hold for nonwords both with and without non-English characters 〈á, é, í, ó, ú, ñ〉, though ratings also seem to be higher on average for nonwords containing at least one non-English character.

The mixed-effects ordinal regression analysis confirms these indications from the raw data. There is a statistically significant main effect of phonotactic score (*β* = 2.178, *z* = 5.105, 95% CI [1.342, 3.014], *p* < 0.001), as well as a significant main effect of the presence of non-English characters (*β* = 0.884, *z* = 3.478, 95% CI [0.386, 1.382], *p* < 0.001), and these two effects do not interact. Thus, participants’ ratings are separately influenced by phonotactic score and by non-English characters. Furthermore, the effect of phonotactic score cannot be reduced to an effect of how “Spanish-like” or “English-like” the written stimuli *look*, as measured by how closely they follow gradient Spanish or English orthotactics; while Spanish and English orthotactic scores both play a role in participant’s rating decisions, each does so independently of, and more weakly than, Spanish phonotactic score (for details, see the S2 File Detailed Analysis and Results Supplement, Sections 4.1–4.2).

We interpret the effect of phonotactic score as a direct confirmation of the suggestion from Experiment 1 that participants have implicit phonotactic knowledge of Spanish. Given the results of Experiment 1, as well as assumptions in the literature that phonotactic knowledge is sourced from generalizations over a large memory store of wordforms (Section 2.2), we take this confirmation as further evidence that participants have a Spanish proto-lexicon. As in Experiment 1, we interpret the effect of non-English characters as an indicator of visual salience, in a way that is not directly related to the proto-lexicon.

The results of Experiment 2 replicate the findings of the wellformedness rating experiment of [34]. However, as in Experiment 1, there appear to be differences in the strength of the effects across the two studies. [34] found a strong effect of phonotactic score, whereby increasing from −1.2 to −0.8 on the phonotactic score scale (i.e., increasing phonotactic probability by a factor of 2.5, from an average conditional probability of approximately 1/16 per phoneme to approximately 1/6) was associated with an increase of approximately 0.75 points on the wellformedness rating scale, on average, for non-Māori-speaking New Zealanders. Here, we appear to have a weaker effect of phonotactic score, whereby the same increase on the phonotactic score scale is associated with an increase of approximately 0.5 points on the wellformedness rating scale, on average. As in Experiment 1, a difference of this sort would follow straightforwardly from the implications that the structural differences between Māori and Spanish have for the ability to estimate phonotactic probabilities firmly from a small set of lexical items, though it may also be affected by differences in other factors such as attitudes and exposure.

## 5 Modeling the proto-lexicon

The results presented in Section 4 establish that participants have implicit lexical and phonotactic knowledge of Spanish. In this section, we use simulations to identify the source of participants’ phonotactic knowledge.

As described previously (Sections 2.1–2.2), the literature typically assumes that phonotactic knowledge derives from generalization over a large set of wordforms, stored in memory (but see e.g. [31] for an alternative view that phonotactic knowledge may be learned directly). In the absence of explicit and fully-fledged knowledge in the form of a lexicon, this large set of wordforms constitutes a proto-lexicon. Here, we construct proto-lexicons with various different properties and use them to generate different phonotactic scoring systems for experimental stimuli. Each proto-lexicon in our analysis consists of a given number of phonological forms with given properties, which we use to train a new Witten-Bell-smoothed trigram language model over phonemes. We use this model to calculate a new phonotactic score for each experimental stimulus, which we substitute into the ordinal regression model of participants’ wellformedness ratings of nonwords in Experiment 2 (Section 4.2). We compare different proto-lexicons to ask which properties give rise to phonotactic scores that permit best explanation of participants’ wellformedness ratings.

First, in Section 5.1, we assume that the proto-lexicon is composed of words, and we ask how many words are necessary. Then, in Section 5.2, we consider whether the proto-lexicon could instead consist of statistically recurring parts of words, called *morphs*. We ask how many morphs are necessary, and whether there is evidence that participants attempt to parse stimuli into morphs in order to evaluate their phonotactic wellformedness.

Throughout, we use Monte Carlo analyses involving fixed effects (logit) ordinal regression and model comparison using the Akaike Information Criterion (AIC) [81], following [34]. To enable more targeted comparison of individual representative models, we supplement these analyses with mixed effects ordinal regression based on the average phonotactic scores across corresponding Monte Carlo samples. These latter analyses do not reflect any single fixed proto-lexicon, but rather the assumption of a mixture of proto-lexicons across a population of individuals.

We describe the analyses at a high level in the relevant sections below; for full details, including a description of the calculation of phonotactic scores, see the S1 File Detailed Materials and Methods Supplement, Sections 4–5.

### 5.1 A proto-lexicon composed of words

The results of Experiment 2 (Section 4.2) showed that participants’ wellformedness ratings are sensitive to phonotactic scores, indicating that they have knowledge of fine-grained phonotactic probabilities. We argued that this knowledge derives from a proto-lexicon, i.e. an implicit memory store of a large number of wordforms. The phonotactic scores used in the analysis were based on nearly 94,000 distinct Spanish wordforms appearing in the SUBTLEX-ESP lexical database [75]. Is it reasonable to assume that participants have this many wordforms in their proto-lexicon? Or can the experimental results be explained just as well—and perhaps ever better—by the assumption of a smaller proto-lexicon?

In a similar analysis conducted by [34], the wellformedness ratings of non-Māori-speaking New Zealanders were explained adequately by the assumption of a proto-lexicon consisting of a subset of around 3,000 wordforms (out of a total of nearly 19,000 unique words with entries in a dictionary), sampled in such a way as to reflect frequencies of occurrence in large corpora. If non-Spanish-speaking Californians and Texans derive their phonotactic knowledge in a similar way, then we expect it to be able to be captured by a similar proto-lexicon, consisting of a subset of wordforms sampled according to frequency.

#### 5.1.1 Approach

To investigate whether the results of Experiment 2 can be explained adequately by the assumption of knowledge of a smaller set of words, we sampled vocabularies of a range of sizes, used them to generate new phonotactic scores, and explored the extent to which these phonotactic scores explained participants’ wellformedness ratings in ordinal regression models. We explored vocabulary sizes between 500 and 90,000 words. For each vocabulary size, we drew 1,000 different samples of that many words from the SUBTLEX-ESP lexical database, either uniformly at random (the *unweighted* sampling scheme), or weighted by the frequency of the words in the database (the *frequency-weighted* sampling scheme). Based on each sample, we constructed phonotactic scores for the experimental stimuli and used them to predict participants’ wellformedness ratings, by substituting the phonotactic score predictor in a fixed effects equivalent of the ordinal regression analysis presented in Section 4.2. We extracted the AIC score for each model, which represents the amount of error in the model’s predictions, and collated these scores for all models by sampling scheme and vocabulary size. The Monte Carlo analysis consists of comparing these collated AIC scores (where lower scores are better). For details of the interpretation of AIC scores, see the S1 File Detailed Materials and Methods Supplement, Section 5.2.

The Monte Carlo analysis described above does not permit us to control for additional sources of variation within a mixed-effects regression framework, as it is too computationally intensive to fit mixed-effects ordinal regression models based on each sampled phonotactic scoring system. To address this issue, we also constructed a single representative (population-level-averaged) phonotactic scoring system for each vocabulary size, and fit a single mixed-effects regression model for each vocabulary size using this phonotactic scoring system. We compared AIC scores across mixed-effects models with phonotactic scores based on vocabularies of different sizes, in order to gain additional perspective on which size proto-lexicon best explains participants’ wellformedness ratings. For details of the calculation of representative phonotactic scoring systems, see the S1 File Detailed Materials and Methods Supplement, Section 5.3.

#### 5.1.2 Results and discussion

**Fig 3:**
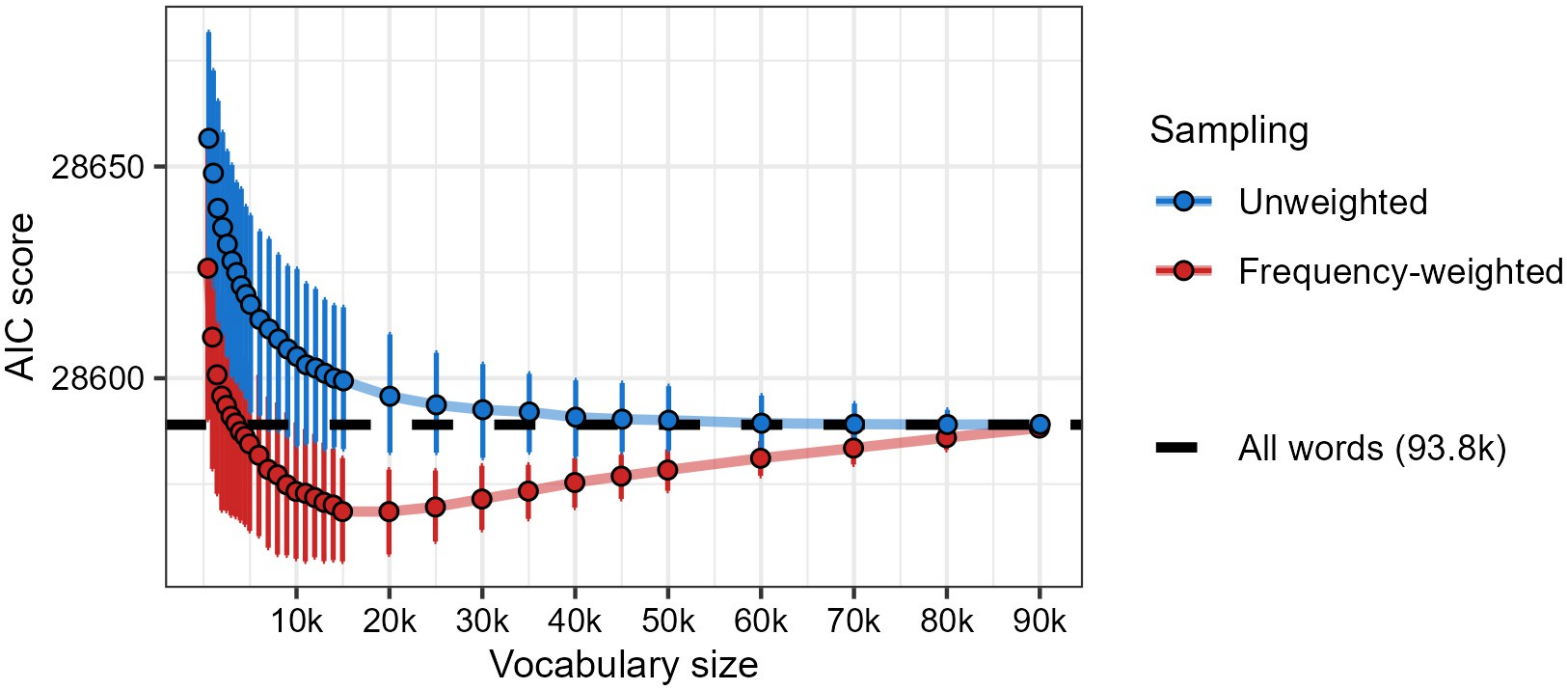
Word-based Monte Carlo analysis of results from Experiment 2, based on 1,000 samples for each vocabulary size. Lower AIC score indicates greater ability to predict participants’ wellformedness judgments. The black dashed line shows the AIC score of a model that assumes phonotactic knowledge generated from the full set of 93,777 words from SUBTLEX-ESP. Points represent mean AIC scores and error bars represent 95% bootstrap percentile intervals.

Fig 3 shows the results of the Monte Carlo analysis. Three patterns are immediately apparent. First, the frequency-weighted sampling scheme consistently enables better explanation of participants’ wellformedness ratings (i.e. lower AIC) than the unweighted sampling scheme. Second, even among the unweighted sampling scheme, phonotactic scores based on a subset of words are able to explain wellformedness ratings at least as well as phonotactic scores based on the full database of nearly 94,000 words. Third, almost all of the phonotactic scoring systems based on frequency-weighted samples of words explain wellformedness ratings even better than phonotactic scores based on the full database.

The first two patterns replicate those seen in the analysis of [34], indicating a commonality in the way that non-Māori-speaking New Zealanders and non-Spanish-speaking Californians and Texans may derive their phonotactic knowledge. In both cases, there is no need to assume that participants have access to the full range of wordforms that may be available to a fluent speaker; their wellformedness ratings can be explained adequately under the assumption of a proto-lexicon consisting of a subset of wordforms that likely occur frequently in their experience.

The third pattern, however, represents a point of departure from previous results. [34] found that a subsampled proto-lexicon consisting of a subset of wordforms could explain participants’ wellformedness ratings as well as a full proto-lexicon consisting of all available wordforms, but not substantially better. Here, the difference is quite stark: for vocabulary sizes of approximately 10,000 wordforms or more, practically all subsampled proto-lexicons in the Monte Carlo analysis explained the participants’ ratings better than the full proto-lexicon, as evidenced by the fact that the 95% boostrap percentile intervals do not include the AIC value seen when the proto-lexicon is assumed to consist of all 93,777 wordforms. A mixed effects regression analysis based on single representative (population-level-averaged) phonotactic scoring systems supports this idea: a proto-lexicon containing 15,000 Spanish wordforms explains participants’ ratings best, with a decrease of more than 20 AIC points compared to the full proto-lexicon (for details, see the S2 File Detailed Analysis and Results Supplement, Section 3.4.2).

While this difference between present results for Spanish and previous results for Māori is no doubt influenced by the fact that the Spanish analyses are based on more wordforms than the Māori analyses, we believe that it is also influenced by the structural differences between the languages. As described in Section 2.3.1, Spanish contains a relatively low number of compounds relative to Māori, and a much larger number of inflected forms. Consequently, larger subsampled proto-lexicons of Spanish are likely to overrepresent inflectional suffixes, which contain very particular phonotactic sequences that are not strongly representative of a large number of base forms, rather than compound components that occur across a variety of base forms. Since a smaller subsampled proto-lexicon of Spanish will contain fewer inflectional suffixes, relative to the number of distinct base forms, it will permit the estimation of phonotactic probabilities that are more representative of wordforms in general.

The Monte Carlo analysis suggests that, if the Spanish proto-lexicon of non-Spanish-speaking Californians and Texans consists of wordforms, it is likely to contain anywhere between 12,000 and 20,000 wordforms. These results raise two important questions: why does there appear to be such a large range of proto-lexicon sizes, and why does the proto-lexicon appear to contain such a large number of wordforms? In response to the first question, we suspect that the wide range of sizes may be related to the wide range of attitudes and exposures to Spanish in California and Texas, as described in Section 2.3; we return to this suspicion in Section 6. In response to the second question, it is possible that the proto-lexicon does not contain the forms of whole words at all, but rather of smaller units that recur across many words. Thus, the appearance of a large proto-lexicon may be an illusion brought about by the combinatorial redundancy of choosing too large a unit as the basis of proto-lexical representations. We explore this possibility in Section 5.2.

### 5.2 What if the proto-lexicon is composed of morphs?

In Section 5.1, we explored proto-lexicons containing full wordforms. However, it is not clear that the proto-lexicon need contain full wordforms. Since the proto-lexicon is built through passive exposure, the units contained within it will be those that a non-speaker of the language can segment out from continuous streams of speech. In the absence of clear and consistent acoustic cues to word boundaries, such units may not be whole words, but rather *parts* of words that recur with statistical regularity. For example, a productive suffix may be segmented out separately from an uncommon stem, since it occurs much more often. We follow [34] in referring to such parts as *morphs*.

In this section, we ask whether there is evidence that the Spanish proto-lexicon consists of morphs rather than words, and if so, how many. That is, we ask whether the wellformedness ratings from Experiment 2 can be better explained by the assumption of phonotactic scores based on a subset of morphs. In parallel, we also ask whether the ratings can be better explained by assuming that participants treat stimuli as potentially composed of multiple morphs, or by assuming that they treat each stimulus as decidedly consisting of a single morph.

In a similar analysis conducted by [34], the wellformedness ratings of non-Māori-speaking New Zealanders were explained better by a morph-based proto-lexicon than a word-based proto-lexicon, as long as participants were assumed to treat stimuli as potentially composed of multiple morphs. In addition, the morph-based proto-lexicon offering best explanation of wellformedness ratings was smaller than the best word-based proto-lexicon, consisting of a subset of approximately 1,500 common morphs (out of nearly 4,000) as opposed to 3,000 common words (out of nearly 19,000). This 50% reduction in size offered by assuming a proto-lexicon composed of morphs rather than words is largely due to the fact that Māori uses strictly concatenative morphology including extensive compounding, which means that a lot of morphs are shared between words, and thus that knowledge of the phonotactic probabilities within a small set of morphs can generalize readily to a larger set of words. Here, we likewise expect that the wellformedness ratings of non-Spanish-speaking Californians and Texans can be well explained by the dual assumptions that the proto-lexicon consists of morphs, and that participants treat stimuli as potentially composed of multiple morphs. However, we do not expect to see as large a reduction in proto-lexicon size when moving from words to morphs as was seen previously for Māori, due to the existence of morphophonological alternations and the relatively low degree of compounding in Spanish (Section 2.3.1). These morphological properties of Spanish limit the extent to which a small set of morphs are phonotactically representative of a large set of words.

#### 5.2.1 Approach

To obtain a pool of Spanish morph types corresponding to the 93,777 word types previously used to generate phonotactic scores, we applied Morfessor 2.0 [82] to the set of word types, converted to phonological forms. Morfessor uses an unsupervised recursive algorithm based on a probabilistic generative model [40] to segment words into morphs in a way that is optimal from the perspective of a Minimum Description Length framework [83]. That is, it identifies the smallest inventory of morphs that best enables a set of words to be compressed efficiently and then reconstructed from this compression faithfully. In this way, it is analogous to a na ive learner who is exposed to, and attempts to segment, a continual stream of wordforms, without access to semantic information. For further details of our use of Morfessor, see the S1 File Detailed Materials and Methods Supplement, Section 4.2.2.

In the set of 93,777 word types, Morfessor identified an inventory of 12,773 distinct morph types. Given this inventory of morphs, we followed the same basic approach outlined previously in Section 5.1: we sampled morph sets of a range of sizes, used them to generate new phonotactic scores, and explored the extent to which these phonotactic scores explained participants’ wellformedness ratings in ordinal regression models. We explored morph set sizes between 500 and 12,000 morphs, where we drew 1,000 different samples for each size, following either an *unweighted* scheme (sampling morphs uniformly at random) or a *frequency-weighted* scheme (sampling morphs weighted by the frequency with which they occur (within words) in the SUBTLEX-ESP database). Based on each sample, we constructed phonotactic scores for the experimental stimuli, used them to predict participants’ wellformedness ratings in a fixed effects ordinal regression model, and extracted the resulting AIC score. As mentioned previously, the phonotactic scores were generated in two ways, depending on whether participants were assumed to treat stimuli as potentially complex and thus attempt to *parse* them into morphs, or whether they were assumed to treat stimuli as simplex and leave them *unparsed*, as single morphs; for details of the generation of these scores, see the S1 File Detailed Materials and Methods Supplement, Section 4.

In the same way as for the word-based Monte Carlo analysis described in Section 5.1.1, we also constructed a single representative (population-level-averaged) phonotactic scoring system for each morph set size, and we compared how well these representative scoring systems explain participants’ wellformedness ratings using mixed-effects ordinal regression. These representative scoring systems assume that participants attempted to parse stimuli into morphs, and are constructed in the manner described in the S1 File Detailed Materials and Methods Supplement, Section 5.3.

#### 5.2.2 Results and discussion

**Fig 4:**
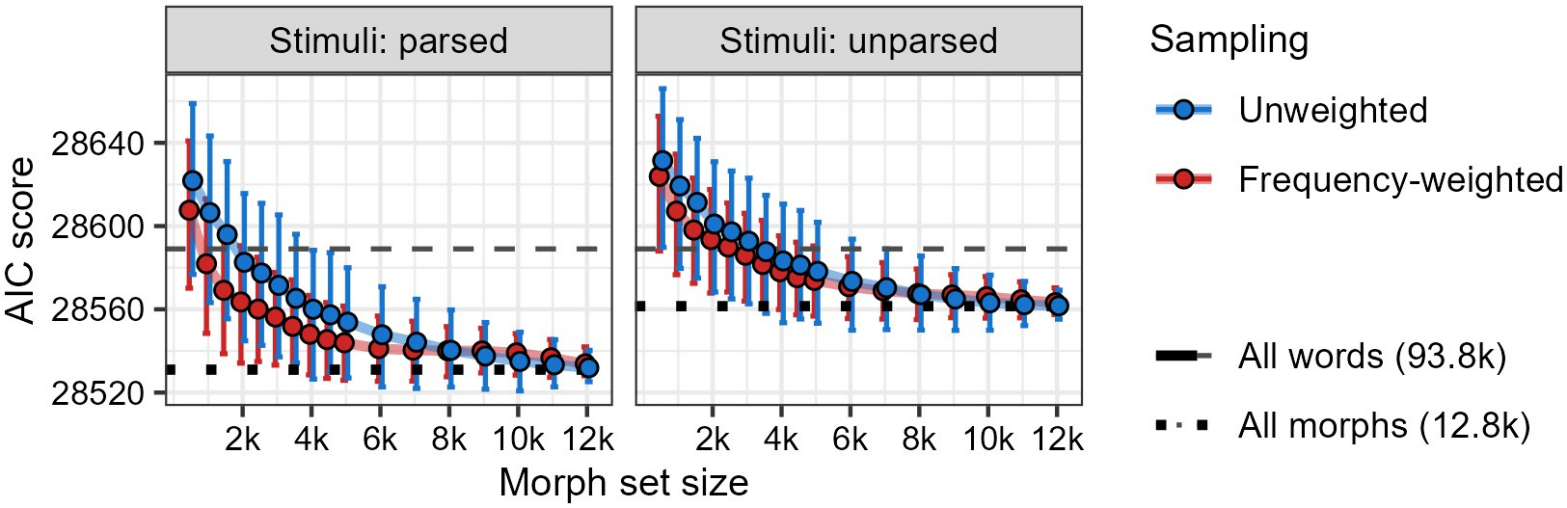
Morph-based Monte Carlo analysis of results from Experiment 2, based on 1,000 samples for each morph set size. Lower AIC score indicates greater ability to predict participants’ wellformedness judgments. The gray dashed line shows the AIC score of a model that assumes phonotactic knowledge generated from the full set of 93,777 words from SUBTLEX-ESP, and the black dotted line shows the AIC score of a model that assumes phonotactic knowledge generated from the full set of 12,773 morphs. The left facet shows the results of assuming that participants are treating stimuli as potentially complex and parsing them into morphs, and the right facet shows the results of assuming that participants are treating stimuli as simplex and leaving them unparsed, as single morphs. Points represent mean AIC scores and error bars represent 95% bootstrap percentile intervals.

Fig 4 shows the results of the Monte Carlo analysis. Four patterns are immediately apparent. First, participants’ wellformedness ratings are explained better under the assumption of a morph-based proto-lexicon than under the assumption of a word-based proto-lexicon consisting of all 93,777 words. Second, participants’ ratings are also explained better under the assumption that they treat stimuli as potentially complex and thus attempt to parse them into morphs. Third, under the two assumptions already described, if participants have a proto-lexicon consisting of 8,000 or fewer morphs, then their ratings are better explained if those morphs are sampled in a frequency-weighted manner than in an unweighted matter. Fourth, while a morph-based proto-lexicon consisting of as few as 6,000 morphs explains participants’ ratings almost as well as a larger proto-lexicon of up to 11,000 morphs (with a difference of mean AIC scores *Δ*AIC ≤ 4.5), a proto-lexicon of 11,000 morphs does not explain participants’ ratings as well, on average, as a full proto-lexicon of 12,773 morphs (with a difference of mean AIC scores *Δ*AIC ≥ 5.6). In fact, a mixed effects regression analysis based on single representative (population-level-averaged) phonotactic scoring systems suggests that no subsampled proto-lexicon explains participants’ ratings *better* than a full proto-lexicon (for details, see the S2 File Detailed Analysis and Results Supplement, Section 3.4.4). This is quite unlike what was seen for word-based proto-lexicons in Section 5.1.2 and for morph-based proto-lexicons of Māori by [34].

The first three patterns replicate the findings of [34] (albeit in a weaker manner for the third one), pointing again to a commonality in the way that non-Māori-speaking New Zealanders and non-Spanish-speaking Californians and Texans may derive their phonotactic knowledge. This is to be expected, given the assumption that phonotactic knowledge derives from a proto-lexicon that is built automatically through passive exposure. It makes sense that the fundamental units of that proto-lexicon should be the statistically recurrent phonological subsequences that a listener without explicit lexical knowledge could plausibly identify and extract from streams of speech. It also makes sense that the same process of identifying morphs utilized in the construction of a proto-lexicon should also be used in determining how to apply the phonotactic knowledge generated from that proto-lexicon.

The fourth pattern, however, may indicate a fundamental difference in the proto-lexicons of Māori and Spanish. For Māori, [34] found that the morph-based proto-lexicon offering best explanation of wellformedness ratings contained substantially fewer than the total possible number of morphs (fewer than 40% of the total possible morphs) and was substantially smaller than the corresponding word-based proto-lexicon (representing a reduction in size of approximately 50%). For Spanish, a morph-based proto-lexicon offering best explanation of participants’ wellformedness ratings contains all 12,773 morphs (100% of the total possible morphs) and represents a reduction in size of less than 15% compared to the word-based proto-lexicon offering best explanation of wellformedness ratings (15,000 words; Section 5.1.2). Put another way, the reduction in proto-lexicon size offered by moving from words to morphs as the assumed units proto-lexical units is substantial for Māori, but not for Spanish. As described in Section 5.2, this difference is expected given differences in the morphological structures of Māori and Spanish; namely, the fact that Māori uses strictly concatenative morphology with extensive compounding, while Spanish does not.

The morphological structure of Spanish also provides an answer to the question of why subsampling the Spanish proto-lexicon does not improve the ability to explain participants’ wellformedness ratings if the proto-lexicon is assumed to consist of morphs, but does if it is assumed to consist of words. At the core of this answer is the fact that Spanish involves a substantial amount of inflection (Section 2.3.1). As discussed in Section 5.1.2, large word-based proto-lexicons offer reduced explanation of wellformedness ratings because they over-represent inflectional suffixes, since they are common to large numbers of words. The same disadvantage is not seen in morph-based proto-lexicons because each inflectional suffix is separated out into its own morph, and each morph can only be represented in the proto-lexicon once. Thus, inflectional suffixes are likely represented in all morph-based proto-lexicons (due to their high frequency), but are represented equally in each of these proto-lexicons; larger morph-based proto-lexicons can thus represent a more diverse range of bases without causing over-representation of inflectional suffixes.

Due to these two consequences of the morphological structure of Spanish (limited reduction in proto-lexicon size going from words to morphs, and limited utility of subsampling a proto-lexicon of morphs), it is little surprise that the Spanish proto-lexicon appears to be substantially larger than the Māori proto-lexicon (over 12,000 morphs for Spanish, vs. approximately 1,500 morphs for Māori). While the notion that non-Spanish-speaking Californians and Texans may have an implicit memory store of more than 12,000 morphs may seem implausible, it is consistent with the enormous storage capacity implicit in neural networks [84] and with episodic models of human memory in which perceptual traces are constantly being stored [85]. It is also consistent with experimental results in which extensive information about surface-level characteristics of a 1000-word artificial language was stored in memory for as long as three years on the basis of just ten 1-hour auditory exposure blocks [10]. Nevertheless, it is somewhat surprising that non-Spanish-speaking Californians and Texans appear to have such a large Spanish proto-lexicon, given that their lexical and phonotactic knowledge appears to be quite weak. We return to discuss this point further, and its implications for the theory and modeling of the proto-lexicon, in Section 7.

## 6 Probing individuals’ knowledge

So far, we have established that non-Spanish-speaking Californians and Texans show evidence of lexical and phonotactic knowledge of Spanish, which, we have argued, derives from a proto-lexicon constructed automatically from passive exposure to Spanish. These results replicate those seen for non-Māori-speaking New Zealanders in the studies reported by [34], with some small apparent differences in the details and strength of effects. We have argued that some of these differences likely derive from structural differences between Spanish and Māori, but it is also possible that they derive from differences in attitudes that participants may hold toward Spanish and Māori, as described in Section 2.3.2. Given the inhibitory roles that negative attitudes can play in perception and memory of language [74, 75], we might expect that the appearance of weaker effects for Spanish than for Māori is driven, in part, by the existence of more negative attitudes toward Spanish among Californians and Texans than toward Māori among New Zealanders. To test this idea, we now examine participants’ knowledge on an individual level, asking whether participants with more negative attitudes show weaker evidence of lexical and phonotactic knowledge.

Examining individuals’ knowledge also enables us to explore the extent to which participants’ exposure to Spanish determines their implicit knowledge of it. Since the proto-lexicon account assumes that knowledge derives from exposure, we may expect to find that participants with more exposure to Spanish show evidence of more implicit knowledge. However, given that [34] did not find clear-cut statistical evidence for such an effect, it is also possible that even small degrees of exposure, accumulated over many years, are sufficient to reach the limits of proto-lexical construction (i.e., to construct as large a proto-lexicon as can be expected through ambient exposure). In this case, we would not expect to see any statistically significant effect of Spanish exposure on degree of evidence for implicit lexical and phonotactic knowledge. We ask what effect, if any, different degrees of exposure to Spanish appear to have on participants’ individual-level knowledge.

### 6.1 Measuring attitudes, exposure, and effects on knowledge

To measure a participant’s attitude and exposure to Spanish, we use self-ratings from the post-task questionnaire. We divide the notion of attitude into two dimensions, which we call *Spanish value* and *nationalism*. We ask two questions on each dimension, which are based on prior research in the area of cultural (‘racial’) threat [59–63]. Spanish value is based on the extent to which the participant views Spanish and its speakers as making important and positive contributions to their state of residence (California or Texas), as assessed by questions (A) and (B) below; positive Spanish value is assumed to be reflected through favor for promoting Spanish language education through state-level policy, and through viewing Hispanic and Latino cultures as important in the state. Nationalism is based on the participant’s views of linguistic diversity and immigration within the United States as a whole, as assessed by questions (C) and (D) below; high nationalism is assumed to be reflected through favor for prioritizing English over other languages in the United States, and through opposition to immigration. For each dimension, we calculate a score by combining the ratings that the participant gives to the two questions, each measured on a 5-point scale, by adding them or subtracting one from the other (based on their polarity). These two dimensions are not highly correlated with each other (polychoric *ρ* ≈ −0.14 in both experiments), and they capture underlying structure revealed by an exploratory factor analysis; for details, see the S2 File Detailed Analysis and Results Supplement, Sections 2.2 (correlations in Experiment 1), 3.2 (correlations in Experiment 2), and 4.3 (exploratory factor analysis).

We also calculate a score for the participant’s self-rated exposure to Spanish in the same way, based on exposure through both the media and social interaction, as assessed by questions (E) and (F) below. The exposure scores are positively correlated with Spanish value (polychoric *ρ* = 0.53 in Experiment 1, *ρ* = 0.25 in Experiment 2) and are not consistently correlated with nationalism across experiments (polychoric *ρ* = −0.24 in Experiment 1, *ρ* = 0.03 in Experiment 2); for details, see the S2 File Detailed Analysis and Results Supplement, Sections 2.2 and 3.2.

For the labels of the scale used for each question, as well as details of score calculation, see the S1 File Detailed Materials and Methods Supplement, Sections 5.4–5.5.

Spanish value
  (A) *How do you feel about the following statement?* Some Spanish language education should be compulsory in school for all children in California/Texas.
  (B) *How do you feel about the following statement?* Hispanic and Latino cultures are important in California/Texas.
Nationalism
  (C) *How do you feel about the following statement?* People in the United States should speak English, not foreign languages.
  (D) *How would you complete the following statement?* I think that the number of immigrants from foreign countries who are permitted to come to the United States to live should be _______.
Exposure
  (E) How often do you think you are exposed to the Spanish language in your daily life, by means of media such as Spanish radio, Spanish TV, online media, etc.?
  (F) How often do you think you are exposed to the Spanish language in your daily life, in conversation at work, at home, or in social settings?

For both attitudes and exposure, the use of self-reported responses presents limitations: we only know how participants *report* feeling or how much exposure they *estimate* having. In addition, the use of a small number of specific questions to gauge general constructs leaves room for undesired external influences: for example, participants could give a negative response to (A) based on their feelings about the educational system or the relative prioritization of Spanish and a third language, rather than based on a perceived negative value of Spanish per se. Similarly, they could respond to questions (E) and (F) to indicate little exposure at the time of the experiment, even if they had high degrees of exposure in the past. In the absence of more comprehensive and direct measurements of attitudes and exposure, which were not feasible within the constraints of the present experimental setup, we acknowledge that our measurements are imperfect and may underestimate variation between participants. Nevertheless, the variation that is captured by our measurements appears to be both valid and useful; for example, analyses that use (B) alone as a measure of Spanish value find the same key results that we report based on the combination of (A) and (B), showing that our analysis is not derailed by alternatively-motivated responses to Spanish language policy in education (see the S2 File Detailed Analysis and Results Supplement, Section 4.4, for details).

To measure the effect of a participant’s Spanish value, nationalism, and exposure scores on their demonstrated lexical and phonotactic knowledge, we added them to the mixed effects ordinal regression models from Sections 4.1 and 4.2, in interactions with each of the already-established terms. For all models, we added by-item random slopes for attitude and exposure scores, and we followed the same model fitting procedure as earlier; for details, see the S2 File Detailed Analysis and Results Supplement, Sections 2.5 and 3.5.

Due to the limited number of participants retained for analysis after screening (Section 3.3), our investigation of the effects of attitudes and exposure on individual-level knowledge suffers from lower statistical power than we had planned. Consequently, we treat the results—especially null results—as suggestive only, and as an opportunity to identify patterns that should be subjected to further scrutiny in future work.

### 6.2 Word identification

Recall that, in Experiment 1, participants rated word and nonword stimuli for their degree of confidence that they were real Spanish words. The basic results described in Section 4.1 showed evidence of both lexical and phonotactic knowledge, as well as evidence for the visual salience of non-English characters 〈á, é, í, ó, ú, ñ〉. For lexical knowledge, participants gave higher ratings to words than to matched nonwords in general, especially for high-frequency words. For phonotactic knowledge, participants gave stimuli (words and nonwords) higher ratings as their phonotactic scores increased.

**Fig 5:**
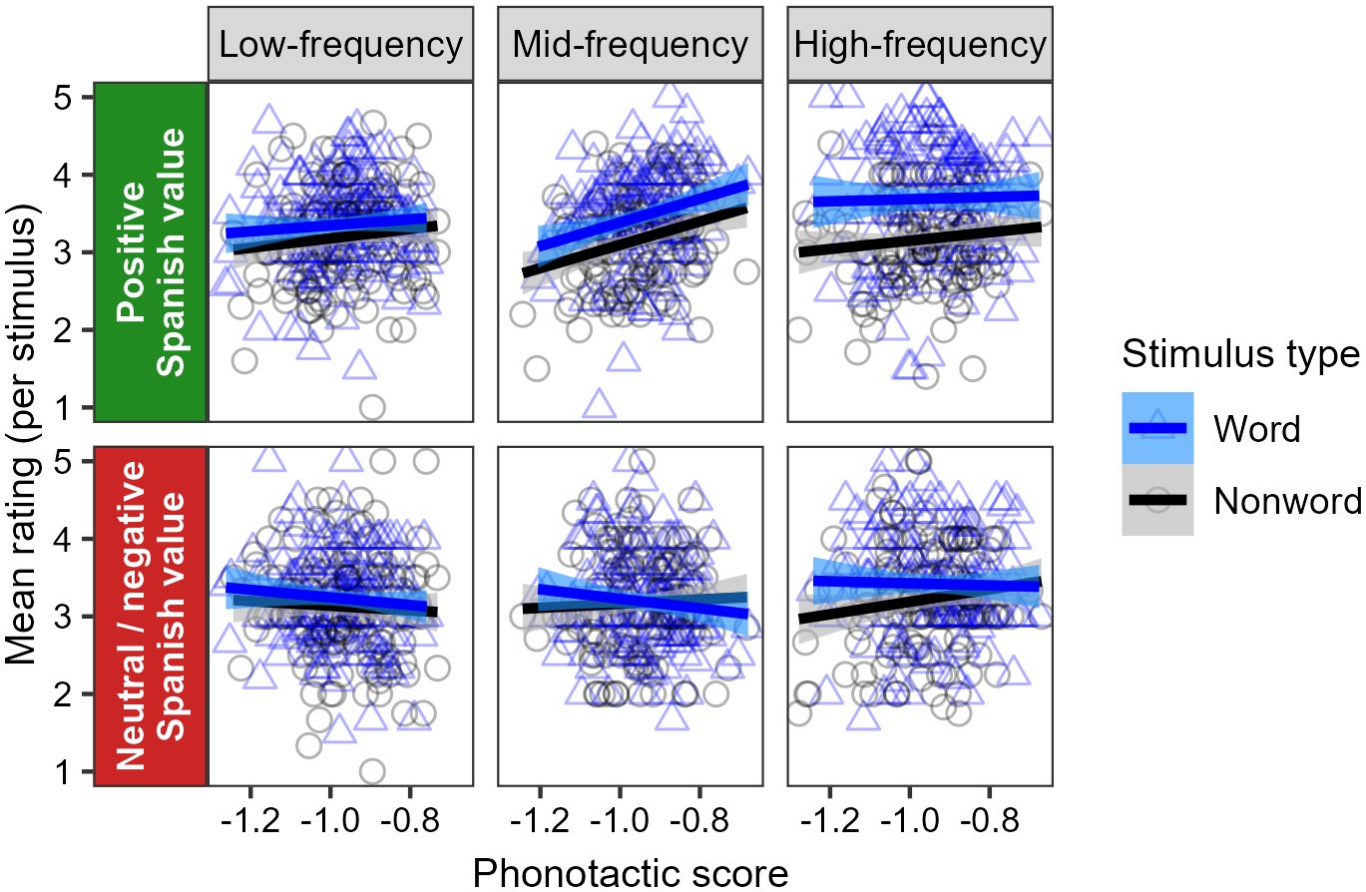
Summary of raw results from Experiment 1, showing participants’ mean wordhood confidence ratings for each stimulus per frequency bin, and their relation to phonotactic score, separating participants with positive Spanish value (top facets) from those with neutral/negative Spanish value (bottom facets). Points represent mean ratings for each real word (blue triangles) and nonword (black circles) within each bin, and straight lines show correlations with phonotactic scores. Participants with positive Spanish value show a separation between ratings for words and nonwords, which grows with word frequency, and give higher ratings as phonotactic score increases; participants with negative Spanish value show neither pattern consistently.

If participants’ construction of a proto-lexicon is affected by attitudes, then we expect to see participants with more positive attitudes toward Spanish and its speakers show more evidence of lexical and phonotactic knowledge. That is, we expect participants with higher Spanish value scores and/or lower nationalism scores to have a greater separation between their ratings for word and nonword stimuli, especially for high-frequency words, and to show a sharper increase in the ratings they give stimuli as phonotactic score increases. We find such effects based on Spanish value, as demonstrated in Fig 5 (but no effects of nationalism).

In the mixed effects ordinal regression model, there is a statistically significant interaction between Spanish value and wordhood status of a stimulus (*β* = 0.164, *z* = 3.479, 95% CI [0.072, 0.257], *p* < 0.001), which further interacts with word frequency (mid- vs. low-frequency: *β* = 0.132, *z* = 1.805, 95% CI [−0.011, 0.275], *p* = 0.071; high- vs. mid-/low-frequency: *β* = 0.189, *z* = 2.776, 95% CI [0.055, 0.322], *p* = 0.006). As shown in the raw data in Fig 5, this interaction sees the separation in ratings between words and nonwords grow with a participant’s attitude toward Spanish and its speakers, and also sees all differences in ratings of words and nonwords practically disappear for participants with strongly negative attitudes. The model also reveals a similar interaction between Spanish value and phonotactic score of a stimulus (*β* = 0.390, *z* = 2.268, 95% CI [0.053, 0.727], *p* = 0.023), whereby the effect of phonotactic score grows more pronounced with a participant’s attitude toward Spanish and its speakers, and disappears entirely for participants with strongly negative attitudes. For a full model summary, see the S2 File Detailed Analysis and Results Supplement, Section 2.5.2.

If participants’ proto-lexicons have not reached their limits by growing to the maximum size that might be expected through ambient exposure, then, given the assumption that the proto-lexicon is construction through automatic processes based on passive exposure, we expect to see participants with more exposure to Spanish showing evidence of more lexical and/or phonotactic knowledge. Our statistical analysis does not reveal any effects of exposure on sensitivity to lexical status and/or phonotactic score: the exposure × wordhood status and exposure × phonotactic score interactions are removed by our model selection process on the basis of neither having coefficients that are significantly different from zero nor contributing significantly to model fit. These null results suggest that participants’ proto-lexicons may indeed have already reached their limits; that is, even small degrees of exposure, accumulated over many years, may be sufficient to ‘fill up’ the Spanish proto-lexicon of a non-Spanish-speaking Californian or Texan, as reported previously by [34] for the Māori proto-lexicon of a non-Māori-speaking New Zealander. However, as noted previously, it is possible that null results such as these are due to lower than planned statistical power; further investigation is required before we can definitively say that large degrees of exposure offer no advantage in constructing implicit knowledge over small degrees of exposure sustained over long periods.

### 6.3 Wellformedness ratings

Recall that, in Experiment 2, participants rated nonword stimuli of a range of phonotactic scores for how Spanish-like they seemed. Similar to the findings for Experiment 1, the basic results described in Section 4.2 showed evidence of phonotactic knowledge and of the visual salience of non-English characters 〈á, é, í, ó, ú, ñ〉. We are primarily interested in phonotactic knowledge, which saw participants give nonwords higher ratings as their phonotactic scores increased.

**Fig 6:**
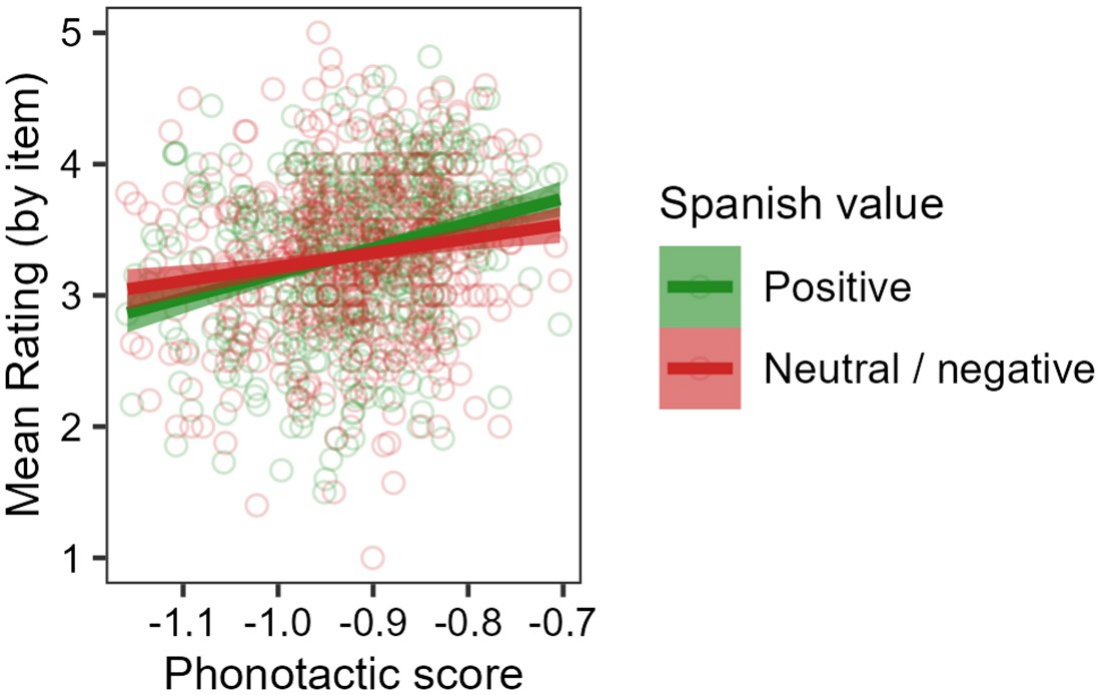
Summary of raw results from Experiment 2, showing participants’ mean wellformedness ratings for each nonword stimulus and their relation to phonotactic score, separating participants with positive Spanish value (green) from those with negative Spanish value (red). Points represent mean ratings for each nonword, and straight lines show correlations with phonotactic scores. In general, ratings increase with phonotactic score, and this pattern appears to be stronger for participants with positive Spanish value than for participants with negative Spanish value; however, the interaction between Spanish value and phonotactic score is not statistically significant.

As in Experiment 1, if participants’ construction of a proto-lexicon is affected by attitudes, then we expect participants with higher Spanish value scores and/or lower nationalism scores to show a sharper increase in the ratings they give stimuli as phonotactic score increases. We see indications of such an effect based on Spanish value in the raw data shown in Fig 6; however, the interaction between Spanish value and phonotactic score is not statistically significant in the mixed effects ordinal regression model (*β* = 0.434, *z* = 1.570, 95% CI [−0.108, 0.975], *p* = 0.116; for a full model summary, see the S2 File Detailed Analysis and Results Supplement, Section 3.5.2). This lack of effect is intriguing, given the presence of such an effect on word identification in Experiment 1, and calls for further investigation; as it stands, it could represent a task-based difference, or a power issue given the large number of participants we had to exclude from our analysis (see Section 3.3).

In the same way as seen for Experiment 1, our statistical analysis does not reveal any effect of a participant’s exposure to Spanish on their phonotactic knowledge, as measured by the degree to which their ratings are sensitive to phonotactic score: the exposure × phonotactic score interaction is removed by our model selection process on the basis of neither having coefficients that are significantly different from zero nor contributing significantly to model fit. If this null result stands up to replication with more statistical power, it would again suggest that the accumulation of small degrees of exposure over many years is sufficient to build an extensive proto-lexicon, as put forth by [34].

## 7 General discussion

In this paper, we set out to answer the following research questions:

(RQ1) Do non-Spanish-speaking Californians and Texans show evidence of implicit lexical and phonotactic knowledge of Spanish?
(RQ2) What is the strength and nature of the implicit Spanish knowledge of Californians and Texans? How might the strength and nature of this knowledge relate to the structure of Spanish, as brought to light by meta-comparison with previous results concerning the implicit Māori knowledge of New Zealanders?
(RQ3) How does the implicit knowledge of Spanish held by non-Spanish-speaking Californians and Texans differ with their attitudes toward Spanish and its speakers?

With regard to (RQ1), our results imply that non-Spanish-speaking Californians and Texans do indeed have implicit lexical and phonological knowledge of Spanish, replicating the findings of [34, 35] for knowledge of Māori among non-Māori-speaking New Zealanders. In both cases, the evidence suggests that this knowledge is underpinned by a vast proto-lexicon of morphs. Our results therefore confirm that the construction of a proto-lexicon in a language that someone doesn’t speak, through ambient exposure to that language in everyday situations, is not limited to the context of Māori in New Zealand. As expected from the widespread observation of statistical learning (Section 2.1), it seems that proto-lexicon construction is an entirely general passive process: humans cannot help but build implicit knowledge of a language when exposed to it often enough.

With regard to (RQ2), we have found that the traces of implicit lexical and phonotactic knowledge observed among non-Spanish-speaking Californians and Texans appear to be quite weak, and that they appear to draw on a relatively large proto-lexicon of morphs. These results are quite distinct from the previous results presented by [34, 35] on implicit knowledge of Māori among non-Māori-speaking New Zealanders; meta-comparison suggests that the knowledge of non-Spanish speakers may be weaker than that of non-Māori speakers, but may be based on a larger proto-lexicon. At first glance, this comparison appears to raise an inconsistency: if implicit linguistic knowledge derives from a proto-lexicon, then shouldn’t a larger proto-lexicon imply more knowledge? This inconsistency can be resolved by consideration of differences in language structure, given the assumption from exemplar-based approaches [28] that representations in the proto-lexicon may have different strengths, based on the extent to which they are reinforced through passive exposure. The phonotactic and morphological structure of Spanish relative to Māori (Section 2.3.1) may facilitate the extraction of a large number of morphs from a speech stream, because there is a large morph inventory that may have relatively consistent phonotactic cues to segmentation, but the representations of these morphs in the proto-lexicon may remain weak, because most of them do not recur often due to the presence of allomorphy and the relative lack of compounding. Moreover, each Spanish morph may contribute relatively less than each Māori morph to the estimation of accurate phonotactic probabilities, given the larger phonological inventory and more complex syllable templates in Spanish than in Māori. Thus, the implicit lexical and phonotactic knowledge that can be formed by accessing or generalizing over the proto-lexicon may also remain weak, in spite of the proto-lexicon being quite large. Our current modeling approach limits us from being able to explore this idea further, as it assumes that all representations in the proto-lexicon have the same (“full”) strength. Further modeling work is required to work through the underpinnings and consequences of an alternative assumption of variable-strength representations, and how it would connect to findings that phonotactic knowledge of a language that one knows explicitly appears to be derived from type-level representations that all have the same strength [32, 33]. One possibility is that the representations in the mental lexicon for a language that is explicitly known have simply all reached a strength ceiling, whereas the representations in the proto-lexicon may not have (yet). Through consideration of such possibilities, our findings relating to language structure offer great potential to extend theoretical understanding of the formation of implicit linguistic knowledge and the construction of a proto-lexicon.

Finally, with regard to (RQ3), our results show that the formation of implicit lexical and phonotactic knowledge that non-Spanish-speaking Californians and Texans have of Spanish appears to be related to their attitudes toward Spanish and its speakers. Crucially, those with more positive attitudes—i.e. those that place more value on Spanish and its speakers in the context of their state—show evidence of more lexical and phonotactic knowledge, and these differences do not seem to be driven by differences in degree of exposure to Spanish. This finding not only extends current understanding of statistical learning of language and the proto-lexicon, but also adds support to claims of a dual-route language processing system, in which social and linguistic factors jointly shape cognitive representations of language [86]. It is consistent with results from the speech perception literature that words spoken in low-prestige accents or accents that evoke negative attitudes are perceived less accurately and encoded in memory less strongly than words spoken in high-prestige accents or accents that evoke positive attitudes [74, 75]. Such results suggest a possible explanation for the relation between attitudes and implicit linguistic knowledge: negative attitudes toward Spanish and its speakers may affect both the size of the proto-lexicon and the strength of representations contained within it, by introducing perceptual variability that makes it harder to identify statistically recurrent morphs, or by otherwise reducing the strength with which extracted morphs are encoded. Further work is required to evaluate this intriguing possibility. If it is true, one major implication would be that the statistical learning of language, while passive and powerful, is also deeply *social*; not only is it facilitated by actual social interaction as opposed to asocial exposure [87], but it is also facilitated by some kind of social investment in such interaction, as reflected by positive attitudes. Of course, our results are correlational, so it is also possible that statistical learning plays a role a role in shaping attitudes, in addition to or instead of the reverse.

We have focused on the acquisition of implicit knowledge of a second language through passive exposure, but our findings also have implications for first language acquisition. For example, the idea that the proto-lexicon may be composed of variable-strength representations suggests that first-language phonotactic knowledge among infants may show effects of token frequency of words and morphs, instead of or in addition to the effects of type frequency that have been found for adults (cf. [32, 33]). There is some support for this suggestion; for example, [88] find that, when young children are exposed to words spoken by multiple distinct talkers, both type and token frequency contribute to the degree to which they are able to accurately produce consonant clusters from those words in other words, which is taken to reflect the degree to which they form generalizable phonotactic knowledge.

More broadly, our results also have implications for practices that hinge upon language learning, such as second language education and language revitalization. Studies indicate that adults who are exposed to a language they don’t speak and thereby form some sort of proto-lexicon have advantages in the explicit learning of vocabulary (i.e. form-meaning mappings) of that language [89–92]. Thus, second language education and pedagogy may find benefits simply from encouraging learners to immerse themselves in ambient exposure to the target language. Furthermore, our results imply that such immersion will be more effective if learners hold positive attitudes toward the target language. This implication complements work indicating that language classroom performance is affected by “integrativeness” and other forms of attitudes toward a language and/or its speakers [93, 94], thereby bolstering the importance of fostering positive attitudes among language students. Similar implications hold for the context of language revitalization, where a key goal is to build the number of speakers of an endangered language. In this context, our results reinforce the value of strong efforts in both the linguistic and social domains; for example, creating a broad range of language resources that facilitate ambient exposure whilst also building general awareness of—and interest in—the language, and investing in efforts to counter ideologies that portray the language as undesirable (e.g. based on notions of standardness) and instead promote positive attitudes.

Our work suggests that the implicit learning of language by passive exposure is extremely powerful, and that it may be affected by language structure and attitudes. Much work remains to be done to dig into these suggestions, especially in light of the limitations of the current work. For example, our experiments included only non-Spanish speakers from California and Texas, where there can be expected to be a relatively large degree of ambient Spanish on average, and did not include control groups of fluent Spanish speakers or participants from areas where there is little or no ambient Spanish. In order to better understand the strength of implicit Spanish knowledge held by non-Spanish-speaking Californians and Texans, it is important to replicate the current study with control groups for comparison. In this replication, it would be valuable to address the issues of the current study that caused us to exclude many participants and a few stimuli from analysis, which may have affected our ability to robustly detect fine-grained effects. In addition, in order to explore the generality of the results, as well the suggested effects of language structure and attitudes, it is important to replicate this work in other languages and/or contexts, potentially using more sophisticated methods to measure attitudes and exposure. In ongoing research, we are developing and extending the present work in these and other ways.

## 8 Conclusion

In Word Identification and Wellformedness Rating experiments, we have found evidence that non-Spanish-speaking Californians and Texans have implicit lexical and phonotactic knowledge of Spanish, replicating earlier work that has found implicit lexical and phonotactic knowledge of Māori in non-Māori-speaking New Zealanders [34, 35]. Through computational modeling, we have shown that, if this implicit knowledge of Spanish derives from a proto-lexicon—a memory store of forms of words and word-parts, extracted from ambient speech via statistical learning—then the proto-lexicon may contain representations for as many as 15,000 forms. However, despite the fact that the Spanish proto-lexicon may be quite large, our results indicate that the implicit knowledge derived from it may be quite weak, at least in comparison to the knowledge derived from smaller proto-lexicons of Māori in earlier work. This weakness of knowledge implies a weakness of the representations in the Spanish proto-lexicon, which we have suggested may be due to structural properties of Spanish that limit (relative to Māori) the ability for a wide range of forms to recur in ambient speech. In addition, we have found evidence that the strength of a non-Spanish-speaking Californian or Texan’s implicit knowledge of Spanish is affected by their attitude toward Spanish and its speakers: the more positive their attitude, the stronger their lexical and phonotactic knowledge. These effects of language structure and attitudes build on the replication of earlier work, extending it new directions. In this way, our results showcase the power and generality of statistical learning of language in adults, while also highlighting how it cannot be divorced from the structural and attitudinal factors that shape the context in which it occurs.

## Supporting information

S1 File Detailed materials and methods supplement. This file contains full details about the participants, stimuli, experimental procedures, phonological forms and phonotactic models, statistical analysis, and questionnaires.
(PDF)

S2 File Detailed analysis and results supplement. This file contains all the code used for data exclusion, analysis, and plotting. It describes each step of the analysis and includes all statistical results (e.g. model summaries).
(HTML)

## References

[1] Aslin RN. Statistical Learning: A Powerful Mechanism that Operates by Mere Exposure. Wiley Interdisciplinary Reviews: Cognitive Science. 2017;8(1-2):1–7. doi: 10.1002/wcs.1373 27906526 PMC5182173

[2] Thiessen ED, Kronstein AT, Hufnagle DG. The Extraction and Integration Framework: A Two-Process Account of Statistical Learning. Psychological Bulletin. 2013;139(4):792–814. doi: 10.1037/a0030801 23231530

[3] Jusczyk PW, Friederici AD, Wessels JMI, Svenkerud VY, Jusczyk AM. Infants’ Sensitivity to the Sound Patterns of Native Language Words. Journal of Memory and Language. 1993;32(3):402–420. doi: 10.1006/jmla.1993.1022

[4] Jusczyk PW, Luce PA, Charles-Luce J. Infants’ Sensitivity to Phonotactic Patterns in the Native Language. Journal of Memory and Language. 1994;33(5):630–645. doi: 10.1006/jmla.1994.1030

[5] Chambers KE, Onishi KH, Fisher C. Infants Learn Phonotactic Regularities from Brief Auditory Experience. Cognition. 2003;87(2). doi: 10.1016/s0010-0277(02)00233-0 12590043

[6] Gullberg M, Roberts L, Dimroth C, Veroude K, Indefrey P. Adult Language Learning After Minimal Exposure to an Unknown Natural Language. Language Learning. 2010;60(SUPPL. 2):5–24. doi: 10.1111/j.1467-9922.2010.00598.x

[7] Richtsmeier P. Word-Types, Not Word-Tokens, Facilitate Extraction of Phonotactic Sequences by Adults. Laboratory Phonology. 2011;2(1). doi: 10.1515/labphon.2011.005 34531931 PMC8443219

[8] Christophe A, Dupoux E, Bertoncini J, Mehler J. Do Infants Perceive Word Boundaries? An Empirical Study of the Bootstrapping of Lexical Acquisition. Journal of the Acoustical Society of America. 1994;95(3):1570–1580. doi: 10.1121/1.408544 8176060

[9] Mattys SL, Jusczyk PW. Phonotactic Cues for Segmentation of Fluent Speech by Infants. Cognition. 2001;78(2):91–121. doi: 10.1016/S0010-0277(00)00109-8 11074247

[10] Frank MC, Tenenbaum JB, Gibson E. Learning and Long-Term Retention of Large-Scale Artificial Languages. PLoS ONE. 2013;8(1):1–6. doi: 10.1371/journal.pone.0052500 23300975 PMC3534673

[11] Archer SL, Curtin S. Nine-Month-Olds Use Frequency of Onset Clusters to Segment Novel Words. Journal of Experimental Child Psychology. 2016;148:131–141. doi: 10.1016/j.jecp.2016.04.004 27181298

[12] Saffran JR, Aslin RN, Newport EL. Statistical Learning by 8-Month-Old Infants. Science. 1996;274(5294):1926–1928. doi: 10.1126/science.274.5294.1926 8943209

[13] Saffran JR, Newport EL, Aslin RN, Tunick RA, Barrueco S. Incidental Language Learning: Listening (and Learning) out of the Corner of Your Ear. Psychological Science. 1997;8(2):101–105. doi: 10.1111/j.1467-9280.1997.tb00690.x

[14] Pelucchi B, Hay JF, Saffran JR. Statistical Learning in a Natural Language by 8-Month-Old Infants. Child Development. 2009;80(3):674–685. doi: 10.1111/j.1467-8624.2009.01290.x 19489896 PMC3883431

[15] Kittleson MM, Aguilar JM, Tokerud GL, Plante E, Asbøjrnsen AE. Implicit Language Learning: Adults’ Ability to Segment Words in Norwegian. Bilingualism. 2010;13(4):513–523. doi: 10.1017/S1366728910000039 21512605 PMC3079201

[16] Ngon C, Martin A, Dupoux E, Cabrol D, Dutat M, Peperkamp S. (Non)words, (Non)words, (Non)words: Evidence for a Protolexicon During the First Year of Life. Developmental Science. 2013;16(1):24–34. doi: 10.1111/j.1467-7687.2012.01189.x 23278924

[17] Palmer SD, Hutson J, Mattys SL. Statistical Learning for Speech Segmentation: Age-Related Changes and Underlying Mechanisms. Psychology and Aging. 2018;33(7):1035–1044. doi: 10.1037/pag0000292 30247045 PMC6233520

[18] Tincoff R, Jusczyk PW. Some Beginnings of Word Comprehension in 6-Month-Olds. Psychological Science. 1999;10(2):172–175. doi: 10.1111/1467-9280.00127

[19] Bergelson E, Swingley D. At 6-9 Months, Human Infants Know the Meanings of Many Common Nouns. Proceedings of the National Academy of Sciences of the United States of America. 2012;109(9):3253–3258. doi: 10.1073/pnas.1113380109 22331874 PMC3295309

[20] Tincoff R, Jusczyk PW. Six-Month-Olds Comprehend Words that Refer to Parts of the Body. Infancy. 2012;17(4):432–444. doi: 10.1111/j.1532-7078.2011.00084.x 32693484

[21] Thiessen ED, Girard S, Erickson LC. Statistical Learning and the Critical Period: How a Continuous Learning Mechanism Can Give Rise to Discontinuous Learning. Wiley Interdisciplinary Reviews: Cognitive Science. 2016;7(4):276–288. 27239798 10.1002/wcs.1394

[22] Hartshorne JK, Skorb L, Dietz SL, Garcia CR, Iozzo GL, Lamirato KE, et al. The Meta-Science of Adult Statistical Word Segmentation: Part 1. Collabra: Psychology. 2019;5(1):1–24. doi: 10.1525/collabra.181

[23] Hartshorne JK, Ricketts W. Evaluating Unsupervised Word Segmentation in Adults: A Meta-Analysis. In: Culbertson J, Perfors A, Rabagliati H, Ramenzoni V, editors. Proceedings of the 44th Annual Conference of the Cognitive Science Society. Austin, TX: Cognitive Science Society; 2022. p. 3500–3507.

[24] Isbilen ES, Christiansen MH. Statistical Learning of Language: A Meta-Analysis Into 25 Years of Research. Cognitive Science. 2022;46(9):e13198. doi: 10.1111/cogs.13198 36121309

[25] Swingley D. Contributions of Infant Word Learning to Language Development. Philosophical Transactions of the Royal Society B: Biological Sciences. 2009;364(1536):3617–3632. doi: 10.1098/rstb.2009.0107 19933136 PMC2828984

[26] Erickson LC, Thiessen ED. Statistical Learning of Language: Theory, Validity, and Predictions of a Statistical Learning Account of Language Acquisition. Developmental Review. 2015;37:66–108. doi: 10.1016/j.dr.2015.05.002

[27] Johnson EK. Constructing a Proto-Lexicon: An Integrative View of Infant Language Development. Annual Review of Linguistics. 2016;2:391–412. doi: 10.1146/annurev-linguistics-011415-040616

[28] Pierrehumbert JB. Phonetic Diversity, Statistical Learning, and Acquisition of Phonology. Language and Speech. 2003;46(2-3):115–154. doi: 10.1177/00238309030460020501 14748442

[29] Daland R, Pierrehumbert JB. Learning Diphone-Based Segmentation. Cognitive Science. 2011;35(1):119–155. doi: 10.1111/j.1551-6709.2010.01160.x 21428994

[30] Thiessen ED, Saffran JR. When Cues Collide: Use of Stress and Statistical Cues to Word Boundaries by 7- to 9-Month-Old Infants. Developmental Psychology. 2003;39(4):706–716. doi: 10.1037/0012-1649.39.4.706 12859124

[31] Adriaans F, Kager R. Learning Novel Phonotactics from Exposure to Continuous Speech. Laboratory Phonology: Journal of the Association for Laboratory Phonology. 2017;8(1):1–14. doi: 10.5334/labphon.20

[32] Hay J, Pierrehumbert JB, Beckman M. Speech Perception, Well-Formedness and the Statistics of the Lexicon. In: Local J, Ogden R, Temple R, editors. Phonetic Interpretation: Papers in Laboratory Phonology VI. Cambridge: Cambridge University Press; 2004. p. 58–74.

[33] Frisch SA, Large NR, Zawaydeh B, Pisoni DB. Emergent Phonotactic Generalizations in English and Arabic. In: Bybee J, Hopper P, editors. Frequency and the Emergence of Linguistic Structure. Amsterdam: John Benjamins; 2001. p. 159–180.

[34] Oh Y, Todd S, Beckner C, Hay J, King J, Needle J. Non-Māori-Speaking New Zealanders have a Māori Proto-Lexicon. Scientific Reports. 2020;10(1):22318. doi: 10.1038/s41598-020-78810-4 33339844 PMC7749111

[35] Panther F, Mattingley W, Todd S, Hay J, King J. Proto-lexicon Size and Phonotactic Knowledge are Linked in Non-Māori-Speaking New Zealand Adults. Laboratory Phonology. 2023;14(1). doi: 10.16995/labphon.7943

[36] Macalister J. A Survey of Māori Word Knowledge. English Aotearoa. 2004;52:69–73.

[37] Krupa V. The Maori Language. Moscow: Nauka; 1968.

[38] Bauer W. Maori. London: Routledge; 1993.

[39] Harlow R. Lexical Expansion in Maori. Journal of the Polynesian Society. 1993;102(1):99–107.

[40] Creutz M, Lagus K. Unsupervised Models for Morpheme Segmentation and Morphology Learning. ACM Transactions on Speech and Language Processing. 2007;4(1):1–34. doi: 10.1145/1322391.1322394

[41] Todd SJ. The Listener in Language Change [Ph.D. Dissertation]. Stanford University; 2019. Available from: http://purl.stanford.edu/mm624dn7355.

[42] U S Census Bureau. Language Spoken at Home: American Community Survey 2016–2020 (5-Year Estimates), Table S1601; 2020. Available from: https://data.census.gov/cedsci/table?g=0100000US%240400000&tid=ACSST5Y2020.S1601.

[43] Instituto Cervantes. El Español en el Mundo 2020: Anuario del Instituto Cervantes. Alcalá de Henares, Madrid; 2020.

[44] Carreira M. The Vitality of Spanish in the United States. Heritage Languages Journal. 2013;10(3):396––413. doi: 10.46538/hlj.10.3.10

[45] Boas TC, Christenson DP, Glick DM. Recruiting Large Online Samples in the United States and India: Facebook, Mechanical Turk, and Qualtrics. Political Science Research and Methods. 2020;8(2):232–250. doi: 10.1017/psrm.2018.28

[46] U S Census Bureau. Hispanic or Latino, and not Hispanic or Latino by Race: Decennial Census 2020, Table P2; 2020. Available from: https://data.census.gov/cedsci/table?tid=DECENNIALPL2020.P2.

[47] U S Census Bureau. Selected Social Characteristics in the United States: American Community Survey 2006–2010 (5-Year Estimates), Table DP02; 2010. Available from: https://data.census.gov/cedsci/table?t=400%20-%20Hispanic%20or%20Latino%20%28of%20any%20race%29&g=0100000US%240400000&tid=ACSDP5YSPT2010.DP02.

[48] Ziegler JC, Goswami U. Reading Acquisition, Developmental Dyslexia, and Skilled Reading Across Languages: A Psycholinguistic Grain Size Theory. Psychological Bulletin. 2005;131(1):3–29. doi: 10.1037/0033-2909.131.1.3 15631549

[49] Campos-Astorkiza R. The Phonemes of Spanish. In: Hualde JI, Olarrea A, O’Rourke E, editors. The Handbook of Hispanic Linguistics. Malden, MA: Blackwell; 2012. p. 89–110.

[50] Colina S. Syllable Structure. In: Hualde JI, Olarrea A, O’Rourke E, editors. The Handbook of Hispanic Linguistics. Malden, MA: Blackwell; 2012. p. 133–151.

[51] Lang MF. Spanish Word Formation: Productive Derivational Morphology in the Modern Lexis. New York, NY: Routledge; 1990.

[52] Pérez Saldanya M. Morphological Structure of Verbal Forms. In: Hualde JI, Olarrea A, O’Rourke E, editors. The Handbook of Hispanic Linguistics. Malden, MA: Blackwell; 2012. p. 227–246.

[53] Varela S. Derivation and Compounding. In: Hualde JI, Olarrea A, O’Rourke E, editors. The Handbook of Hispanic Linguistics. Malden, MA: Blackwell; 2012. p. 209–226.

[54] Moyna MI. Compound Words in Spanish: Theory and History. Philadelphia, PA: John Benjamins; 2011.

[55] Eddington D. Morphophonological Alternations. In: Hualde JI, Olarrea A, O’Rourke E, editors. The Handbook of Hispanic Linguistics. Malden, MA: Blackwell; 2012. p. 193–208.

[56] Blommaert J. Language Policy and National Identity. In: Ricento T, editor. An Introduction to Language Policy: Theory and Method. Malden, MA: Blackwell; 2006. p. 238–254.

[57] Porcel J. Language Maintenance and Language Shift among US Latinos. In: Díaz-Campos M, editor. The Handbook of Hispanic Sociolinguistics. Malden, MA: Wiley-Blackwell; 2011. p. 623–645.

[58] Ricento TK. National Language Policy in the United States. In: Ricento TK, Burnaby B, editors. Language and Politics in the United States and Canada: Myths and Realities. Mahwah, NJ: Lawrence Erlbaum; 1998. p. 85–112.

[59] Rocha RR, Espino R. Racial Threat, Residential Segregation, and the Policy Attitudes of Anglos. Political Research Quarterly. 2009;62(2):415–426. doi: 10.1177/1065912908320931

[60] Newman BJ, Hartman TK, Taber CS. Foreign Language Exposure, Cultural Threat, and Opposition to Immigration. Political Psychology. 2012;33(5):635–657. doi: 10.1111/j.1467-9221.2012.00904.x

[61] Hempel LM, Dowling JA, Boardman JD, Ellison CG. Racial Threat and White Opposition to Bilingual Education in Texas. Hispanic Journal of Behavioral Sciences. 2013;35(1):85–102. doi: 10.1177/0739986312461626

[62] Hopkins DJ, Tran VC, Williamson AF. See No Spanish: Language, Local Context, and Attitudes Toward Immigration. Politics, Groups, and Identities. 2014;2(1):35–51. doi: 10.1080/21565503.2013.872998

[63] Shin H, Leal DL, Ellison CG. Does Anti-Hispanic Bias Motivate Opposition to Non-English Languages? Sociological Inquiry. 2015;85(3):375–406.

[64] Hood MV III, Morris IL. ¿Amigo o Enemigo?: Context, Attitudes, and Anglo Public Opinion toward Immigration. Social Science Quarterly. 1997;78(2):309–323.

[65] Stein RM, Post SS, Rinden AL. Reconciling Context and Contact Effects on Racial Attitudes. Political Research Quarterly. 2000;53(2):285–303. doi: 10.1177/106591290005300204

[66] Alamillo L, Palmer D, Viramontes C, García EE. California’s English-Only Policies: An Analysis of Initial Effects. In: Valenzuela A, editor. Leaving Children Behind: How “Texas-style” Accountability Fails Latino Youth. Albany, NY: State University of New York Press; 2005. p. 201–224.

[67] Dailey RM, Giles H, Jansma LL. Language Attitudes in an Anglo-Hispanic Context: The Role of the Linguistic Landscape. Language and Communication. 2005;25:27–38. doi: 10.1016/j.langcom.2004.04.004

[68] Aguilar M. Language Attitudes Toward Mexican Spanish-Accented and Standard Varieties of English [M.A. Thesis]. University of Texas at El Paso; 2018. Available from: https://www.proquest.com/dissertations-theses/language-attitudes-toward-mexican-spanish/docview/2175624216/se-2.

[69] Hashimoto D. Loanword Phonology in New Zealand English: Exemplar Activation and Message Predictability [Unpublished doctoral dissertation]. University of Canterbury; 2019. Available from: http://ir.canterbury.ac.nz/handle/10092/16634.

[70] Sibley CG, Liu JH. New Zealand = Bicultural? Implicit and Explicit Associations between Ethnicity and Nationhood in the New Zealand Context. European Journal of Social Psychology. 2007;37:1222–1243. doi: 10.1002/ejsp.459

[71] Giles H, Coupland N, Coupland J. Accommodation Theory: Communication, Context, and Consequence. In: Giles H, Coupland N, Coupland J, editors. Contexts of Accommodation: Developments in Applied Sociolinguistics. Cambridge, UK: Cambridge University Press; 1991. p. 1–68.

[72] Babel M. Dialect Divergence and Convergence in New Zealand English. Language in Society. 2010;39(4):437–456. doi: 10.1017/S0047404510000400

[73] Yu ACL, Abrego-Collier C, Sonderegger M. Phonetic Imitation from an Individual-Difference Perspective: Subjective Attitude, Personality and “Autistic” Traits. PLoS ONE. 2013;8(9). doi: 10.1371/journal.pone.0074746 24098665 PMC3786990

[74] Nguyen N, Shaw JA, Tyler MD, Pinkus RT, Best CT. Affective Attitudes Towards Asians Influence Perception of Asian-Accented Vowels. In: The Scottish Consortium for ICPhS 2015, editor. Proceedings of the 18th International Congress of Phonetic Sciences. Glasgow: University of Glasgow; 2015.Available from: http://www.internationalphoneticassociation.org/icphs-proceedings/ICPhS2015/Papers/ICPHS0561.pdf.

[75] Sumner M, Kataoka R. Effects of Phonetically-Cued Talker Variation on Semantic Encoding. The Journal of the Acoustical Society of America. 2013;134(6):485–491. doi: 10.1121/1.4826151 25669293

[76] Cuetos F, González-Nosti M, Barbón A, Brysbaert M. SUBTLEX-ESP: Spanish Word Frequencies Based on Film Subtitles. Psicológica. 2011;32(2):133–143.

[77] Aguasvivas JA, Carreiras M, Brysbaert M, Mandera P, Keuleers E, Duñabeitia JA. SPALEX: A Spanish Lexical Decision Database from a Massive Online Data Collection. Frontiers in Psychology. 2018;9:2156. doi: 10.3389/fpsyg.2018.02156 30483181 PMC6240651

[78] Keuleers E, Brysbaert M. Wuggy: A Multilingual Pseudoword Generator. Behavior Research Methods. 2010;42(3):627–633. doi: 10.3758/BRM.42.3.627 20805584

[79] Christensen RHB. ordinal—Regression Models for Ordinal Data [R package installed from source dated 8/22/2022]; 2020. Available from: https://github.com/runehaubo/ordinal.

[80] R Core Team. R: A Language and Environment for Statistical Computing [version 4.1.2]; 2021. Available from: https://www.R-project.org/.

[81] Wagenmakers EJ, Farrell S. AIC Model Selection Using Akaike Weights. Psychonomic Bulletin & Review. 2004;11(1):192–196. doi: 10.3758/BF03206482 15117008

[82] Virpioja S, Smit P, Grönroos SA, Kurimo M. Morfessor 2.0: Python Implementation and Extensions for Morfessor Baseline. Helsinki: Department of Signal Processing and Acoustics, Aalto University; 2013.

[83] Rissanen J. Modelling by Shortest Data Description. Automatica. 1978;14:465–471. doi: 10.1016/0005-1098(78)90005-5

[84] Wang Y, Liu D, Wang Y. Discovering the Capacity of Human Memory. Brain and Mind. 2003;4:189–198. doi: 10.1023/A:1025419826662

[85] Landauer TK. How Much do People Remember? Some Estimates of the Quantity of Learned Information in Long-Term Memory. Cognitive Science. 1986;10(4):477–493. doi: 10.1207/s15516709cog1004_4

[86] Sumner M, Kim SK, King E, McGowan KB. The Socially Weighted Encoding of Spoken Words: A Dual-Route Approach to Speech Perception. Frontiers in Psychology. 2014;4. doi: 10.3389/fpsyg.2013.01015 24550851 PMC3913881

[87] Kuhl PK, Conboy BT, Coffey-Corina S, Padden D, Rivera-Gaxiola M, Nelson T. Phonetic Learning as a Pathway to Language: New Data and Native Language Magnet Theory Expanded (NLM-e). Philosophical Transactions of the Royal Society B: Biological Sciences. 2008;363(1493):979–1000. doi: 10.1098/rstb.2007.2154 17846016 PMC2606791

[88] Richtsmeier P, Gerken L, Ohala D. Contributions of Phonetic Token Variability and Word-Type Frequency to Phonological Representations. Journal of Child Language. 2011;38(5):951–978. doi: 10.1017/S0305000910000371 21126387 PMC7359303

[89] Mirman D, Magnuson JS, Estes KG, Dixon JA. The Link Between Statistical Segmentation and Word Learning in Adults. Cognition. 2008;108(1):271–280. doi: 10.1016/j.cognition.2008.02.003 18355803 PMC2486406

[90] Kuppens AH. Incidental Foreign Language Acquisition from Media Exposure. Learning, Media and Technology. 2010;35(1):65–85. doi: 10.1080/17439880903561876

[91] Bisson MJ, van Heuven WJB, Conklin K, Tunney RJ. Incidental Acquisition of Foreign Language Vocabulary through Brief Multi-Modal Exposure. PLoS ONE. 2013;8(4). doi: 10.1371/journal.pone.0060912 23579363 PMC3620316

[92] Mattingley W, Panther F, King J, Hay J, Todd S, Keegan P. Awakening the Proto-Lexicon: A Proto-Lexicon Gives Learning Advantages for Intentionally Learning a Language; under review.

[93] Spolsky B. Attitudinal Aspects of Second Language Learning. Language Learning. 1969;19(3-4):271–285. doi: 10.1111/j.1467-1770.1969.tb00468.x

[94] Masgoret AM, Gardner RC. Attitudes, Motivation, and Second Language Learning: A Meta-Analysis of Studies Conducted by Gardner and Associates. Language Learning. 2003;53(SUPPL. 1):167–210. doi: 10.1111/1467-9922.00227

