# Supplementary material for: Language structure, attitudes, and learning from ambient exposure: Lexical and phonotactic knowledge of Spanish among non-Spanish-speaking Californians and Texans
Source: PLoS One. 2023 Apr 27;18(4):e0284919. doi: 10.1371/journal.pone.0284919 (PMC10138781; doi:10.1371/journal.pone.0284919)
Supplement: S1 File — This file contains full details about the participants, stimuli, experimental procedures, phonological forms and phonotactic models, statistical analysis, and questionnaires. (PDF) [file pone.0284919.s001.pdf]

Language structure, attitudes, and learning from ambient exposure: Lexical and phonotactic knowledge of Spanish among non-Spanish-speaking Californians and Texans

Detailed Materials and Methods Supplement

Simon Todd[a], Chadi Ben Youssef[a], Alonso Vásquez-Aguilar[a]

[a] *Department of Linguistics, University of California Santa Barbara*

OSF repository

All of the files referenced in this supplement can be found in an OSF repository.

1. Participants

In both experiments, non-Spanish-speaking participants (NSS) were recruited via Amazon Mechanical Turk. Amazon requires all workers in the Mechanical Turk service to be over the age of 18, and we also stated on the first page of each experiment that participants were required to be over 18. The experiments were only visible to workers whose Mechanical Turk account was registered in California or Texas.

A pre-screening questionnaire (Section 6.1) determined eligibility for participation; if a participant did not respond as expected to any screening question, they were blocked from continuing to the experiment. Participants were also screened out during each experiment if they failed more than one of six attention check trials (see Section 3). Participants who were screened out for either of these reasons did not finish the experiment, and their responses are not retained in the dataset.

In addition to screening out participants before or during the experiment, we also removed or excluded a number of participants after they completed the experiment (all of whom were paid for completing the experiment). Some participants who completed an experiment fully were removed from the dataset on suspicion of using automated tools in the experiment or otherwise not fully attending (despite passing the requisite attention checks), while others were kept in the dataset but excluded from the analysis on the basis of responses to a post-task questionnaire. For details of removals and exclusions, see Sections 1.1 and 1.2; for the post-task questionnaire, see Section 6.2).

1.1. Experiment 1

For Experiment 1, we recruited 100 participants who passed the pre-screening questionnaire. Participants were paid US $5 for completing the experiment.

14 participants were removed from the dataset in Experiment 1, on suspicion of using automated tools in the experiment or otherwise not fully attending. The reasons for exclusion were as follows:

- 5 participants were removed for writing the name of a US state in response to a post-task question asking for their gender.

- 1 participant was removed for writing the name of a US state in response to a post-task question asking whether they speak any Romance languages.
- 4 participants were removed for writing inappropriate responses to a post-task question asking which Spanish-speaking countries they have lived in (1 entry of a US state name; 2 entries of a degree of proficiency, e.g. 'well'; 1 entry of 'english').
- 1 participant was removed for skipping 93 trials (due to the use of automated methods, as trials could not be skipped ordinarily).
- 2 participants were removed on suspicion of being duplicates of each other, based on identical or highly similar very specific entries in free fields. Both participants also had skipped trials and a missed attention check, both of which are suggestive of the use of automated methods.

These removals left us with responses from 86 participants, which are included in the dataset distributed with this paper. However, not all of those participants were included in the analysis. In total, 46 participants were excluded from the analysis, for the following reasons:

- *Skipped trials*[1]: 4 participants skipped more than 3 trials.
- *Spanish proficiency*[2]: 18 participants listed Spanish among the languages that they speak well, and a further 6 participants indicated that they could speak or comprehend Spanish at least "fairly well".
- *Spanish education*[2]: 12 participants indicated that they had studied Spanish at college level.
- *Knowledge of related Romance languages*: 1 participant indicated knowledge of a related Romance language, and 1 participant reported having lived in a country in which a Romance language is widely spoken.
- *Uniformity of ratings*: 4 participants had highly uniform ratings across all items, as indicated by the fact that they used a single rating for more than 180 of the 240 trials.

We analyzed data from 40 participants in Experiment 1. For a breakdown of their demographics, see Section 2.2 of the Detailed Analysis and Results Supplement.

[1]Participants could not elect to skip trials. Skipped trials could only be a result of improper engagement with the experiment, e.g. using automated tools or pressing the *Tab* key rapidly between trials.

[2]The large number of participants indicating proficiency or college-level education in Spanish in the post-task questionnaire, despite indicating that they could not hold a basic conversation in Spanish in the pre-screening questionnaire, indicates limitations to our attempts to screening. We can see three possible reasons for this discrepancy: (a) participants were purposefully dishonest in the pre-screening questionnaire; (b) participants did not answer the post-task questionnaire carefully; or (c) participants did not understand the pre-screening and/or post-task questions. In future work, we plan to rewrite the questions and separate the questionnaires from the experiment, so that participants will not know the expected answers to screening questions, and then to invite only those participants who answer the screening questions as expected to complete the experiment.

1.2. Experiment 2

For Experiment 2, we also recruited 100 participants who passed the pre-screening questionnaire. Participants were not restricted based on their participation in Experiment 1. Participants were paid US $5 for completing the experiment.

9 participants were removed from the dataset in Experiment 2, on suspicion of using automated tools in the experiment or otherwise not fully attending. The reasons for exclusion were as follows:

- 3 participants were removed for writing the name of a US state in response to a post-task question asking for their gender.
- 3 participants were removed for writing inappropriate responses to a post-task question asking which Spanish-speaking countries they have lived in (2 entries of a degree of proficiency, e.g. 'well'; 1 entry of 'english').
- 3 participants were removed on suspicion of being duplicates of each other, based on identical or highly similar very specific entries in free fields. These participants all stated they were in California but had IP addresses outside of California, wrote the name of a Californian city in response to a post-task question asking for their ethnicity, and had very similar comments along the lines of "THIS EXPERIMENT IS VERY INTERESTED".

These removals left us with responses from 91 participants, which are included in the dataset distributed with this paper. However, not all of those participants were included in the analysis. In total, 52 participants were excluded from the analysis, for the following reasons:

- *Skipped trials*[1]: 7 participants skipped more than 3 trials.
- *Spanish proficiency*[2]: 13 participants listed Spanish among the languages that they speak well, and a further 6 participants indicated that they could speak or comprehend Spanish at least "fairly well".
- *Spanish education*[2]: 20 participants indicated that they had studied Spanish at college level.
- *Knowledge of related Romance languages*: 1 participant indicated knowledge of a related Romance language, and 1 participant reported having lived in a country in which a Romance language is widely spoken.
- *Uniformity of ratings*: 4 participants had highly uniform ratings across all items, as indicated by the fact that they used a single rating for more than 180 of the 240 trials.

We analyzed data from 39 participants in Experiment 2. For a breakdown of their demographics, see Section 3.2 of the Detailed Analysis and Results Supplement.

2. Stimuli

We obtained stimuli following a two-step process. First, we generated a large set of potential stimuli, including both words and matched nonwords. Then, we selected a subset of potential stimuli, to constitute the pool from which stimuli were sampled on an experiment- and participant-wise manner.

2.1. *Generation of stimuli*

We generated a set of potential real word stimuli and a set of potential nonword stimuli. The set of potential real word stimuli was obtained from the intersection of the Spanish lexical database SPALEX (Aguasvivas, Carreiras, Brysbaert, Mandera, Keuleers & Duñabeitia, 2018) with the Spanish database underlying the multilingual nonword generator Wuggy (Keuleers & Brysbaert, 2010). By intersecting these two databases, we ensured that the real words in the experiment would be "base forms" (i.e. words that are not proper nouns, inflected forms, or compounds) for which highly similar nonwords could be easily generated. The set of potential nonword stimuli was obtained from Wuggy, with reference to the pool of real words. Both sets were then filtered to exclude stimuli with fewer than 5 or more than 8 phonemes and stimuli with the same orthographic form as an English word (including borrowings).

2.1.1. *Potential real words: SPALEX*

We took the potential real word stimuli from SPALEX (Aguasvivas et al., 2018), which contains 44,853 words. We used SPALEX because it only includes "base forms" – i.e. words that are not proper nouns, inflected forms, or compounds – which allows us to minimize potential influences of morphology. SPALEX also lists the frequency of each word, according to the EsPal corpus (Duchon, Perea, Sebastián-Gallés, Martí & Carreiras, 2013).

We did not use the whole SPALEX dataset to generate the stimuli. Instead, we used the subset of 13,318 words that are also contained in the Spanish database used by Wuggy (see Section 2.1.2 below), so that we could easily use the words to generate nonwords. We further reduced the subset by including only words of 5 to 8 phonemes, resulting in a final list of 7,690 potential real word stimuli.

Each word in the final list was put in a frequency bin, based on tokens per million words as recorded in SPALEX. These bins follow standard definitions in psycholinguistics (Brysbaert, Mandera & Keuleers, 2018). We used the following thresholds to generate 3 bins summarized below:

- *Low frequency* (1–10 million words per million): 5,274 words.
- *Mid frequency* (10–100 million words per million): 2,026 words.
- *High frequency* (≥100 million words per million): 390 words.

Each word was then converted to phonological form according to the procedure described in Section 4.1, and had its phonotactic score calculated according to the procedure described in Section 4.3.

2.1.2. *Potential nonwords: Wuggy*

We generated the potential nonword stimuli in such a way as to be highly similar to the potential real word stimuli, using Wuggy (Keuleers & Brysbaert, 2010). Wuggy is a multilingual nonword generator widely used in psycholinguistic experiments. For a given real word, it generates nonword candidates that match the word closely in (orthographic) subsyllabic structure and transition probabilities, in a positionally-sensitive manner and up to a given threshold of deviation. The nonword candidates are returned in a ranked list, according to how closely they match the word in these respects.

We used Wuggy to generate ten nonword candidates for each of the 7,690 words in our list of potential real word stimli. If the original word contained an accented vowel indicating non-default stress, we assigned an accent to the corresponding vowel in each nonword candidate. We converted the candidates to phonological form following the procedure outlined in Section 4.1 and discarded any whose phonemic length did not match that of the original word (e.g. due to the use of a silent ⟨h⟩).[3] We then calculated a phonotactic score for each nonword candidate (see Section 4.3.1), and identified the two candidates with phonotactic scores most similar to the original word.

From these two nonwords candidates for each word, we selected the best via manual inspection. To avoid having to manually inspect all 7,690 word-nonword candidate sets, we chose a subset for inspection, based on the idea that we wanted a similar number of stimuli in each frequency bin, where the word and nonword were closely matched for phonotactic score and did not have extreme (outlier) phonotactic scores. To form this subset, we excluded candidate sets where the phonotactic score of the real word was below −1.5, split the remaining candidate sets according to the frequency bin of the real word, and sorted them from smallest to largest absolute difference in phonotatic score between the real word and the phonotactically-closest nonword candidate. From these sorted bins, we inspected all 390 candidate sets with a high-frequency real word, 372 candidate sets with a mid-frequency real word, and 271 candidate sets with a low-frequency real word. We aimed for there to be at least 250 chosen stimuli in each frequency bin.

The manual inspection was carried out by a fluent Spanish speaker (the third author). For each set of candidate nonwords, he chose the one in each case that seemed most word-like, provided it did not appear to be a proper noun, morphologically complex, homophonous to a real Spanish word, or to have the same orthographic form as an existing English word. If neither nonword candidate in a set was satisfactory according to these criteria, the entire set was discarded.

The result of these efforts was a list of 961 pairs of closely-matched potential word and nonword stimuli.

2.2. Selection of stimuli

From the 961 potential stimulus pairs, we selected a smaller pool of pairs to use in our experiments. This selection process was guided by the desire for a final pool of stimuli that spanned a range of frequency bins, lengths, and phonotactic scores, in a highly controlled manner. We made efforts to ensure: that each word and nonword within a pair were as phonotactically similar to each other as possible; that the same number of stimuli were included in each frequency bin; that the distributions of stimulus lengths across those frequency bins were matched; and that the words and nonwords in different groups defined by length and frequency bin had as similar distributions of phonotactic scores as possible.

We began by excluding any word-nonword pairs in which the word and nonword differed in phonotactic score by more than 0.1. We also excluded any word-nonword pairs in which one member had a phonotactic score lower than −1.3, since manual inspection of phonotactic score distributions indicated that such values were likely outliers. After these exclusions, we were left with 807 potential stimulus pairs, distributed across frequency bins and lengths as shown in Table 1.

[3]As described in Section 4.1, we realized after running the experiment that our original method of conversion to phonological form, which was based on manually-identified letter-to-sound correspondences, had some deficiencies. As such, our original stimulus selection process was also affected by these deficiencies; phonological lengths and phonotactic scores of word-nonword pairs may not have been as closely matched as we intended. We adopted a new method of conversion that accounts for these deficiencies in the analysis, and excluded stimuli with length or

Table 1: Distribution of potential stimuli word-pairs across frequency bins and lengths

Freq. bin		Length	Pairs
Low		5	38
		6	71
		7	60
		8	58
	Total		227
Mid		5	77
		6	88
		7	70
		8	72
	Total		307
High		5	90
		6	76
		7	67
		8	40
	Total		273
	Grand total		807

As shown in Table 1, there were fewer stimulus pairs in the low-frequency bin than in other bins. In order to have the same number of stimuli in each frequency bin, we therefore needed to subsample from the other bins. In order to facilitate matching of phonotactic score distributions across lengths and frequency bins, we decided to also subsample from the high-frequency bin. To preserve the approximate distribution of lengths, we subsampled 170 word-nonword pairs within each frequency bin as follows:

- *Length 5*: 34 word-nonword pairs per bin
- *Length 6:* 51 word-nonword pairs per bin
- *Length 7:* 51 word-nonword pairs per bin
- *Length 8:* 34 word-nonword pairs per bin

To identify subsamples that best match the phonotactic score distributions across lengths and frequency bins, we randomly drew 1,000 sets of subsamples and compared them. Using Jensen-Shannon divergence (Lin, 1991), we identified the subsamples in which the distribution of phonotactic scores within each length group was most similar across frequency bins. Then, we used manual inspection to reduce the number of stimuli with phonotactic scores near the edge of the distribution, by identifying alternative pairs of stimuli that could be substituted without drastically changing the Jensen-Shannon divergence. Figure 1 shows the distribution of phonotactic scores across bins and lengths in the final subsamples.

phonotactic score mismatches at that point.

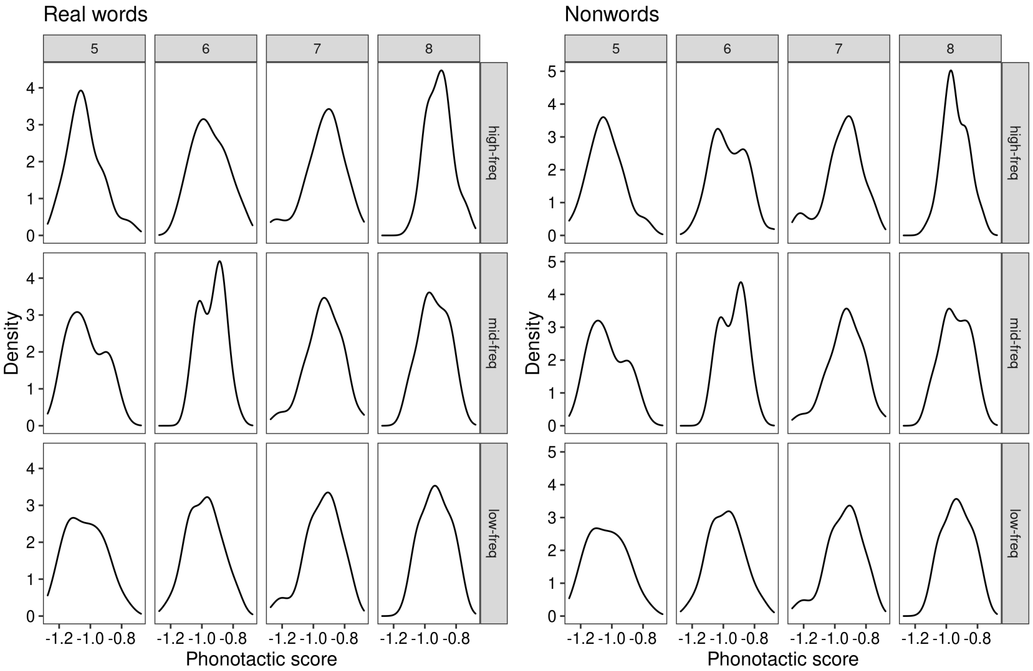

Figure 1: Distribution of phonotactic scores of stimuli in the final stimulus pool, split by lexical status (word or nonword), length, and frequency bin. Note that these distributions were calculated based on the original phonological forms and phonotactic scores used for stimulus selection, not those used for the final analysis (see Section 4.1).

The final stimulus pool contains 510 real word stimuli, each paired with a phonotactically-matched nonword. The frequencies of the real word stimuli in each bin are shown in Table 2 below.

Table 2: Descriptive statistics of each bin in the stimuli dataset

Bin	Min. freq.	Max. freq.	Median	Mean	SD
High	101.3	9585.0	171.3	288.8	745.4
Mid	10.7	89.7	21.1	30.1	20.8
Low	1.0	9.5	2.8	3.4	2.1

The final stimulus pool is available in the file `data/stimuli-pairs.csv` in the OSF repository associated with these supplementary materials. For each word-nonword pair, we list the frequency bin that the pair was assigned to (based on the frequency of the word), the orthographic forms, the phonological forms, the phonotactic scores, and the phonemic lengths.[4]

2.3. Experiment 1

In Experiment 1, each participant responds to 120 words and 120 matched nonwords, broken down by length and frequency bin as shown in Table 3. The word and nonwords are randomly sampled in pairs from the larger pool of 510 stimulus pairs.

After running the experiment, we realized that some stimuli (words and nonwords) that had the same orthographic form as English words had made it into our pool. We excluded responses to these stimuli, and their matched partners, from the analysis. Table 4 lists the excluded pairs, together with their lengths and the frequency bin of the real word.

Table 4: Excluded stimulus pairs where one member has the same orthographic form as an English word. For each pair, the word or nonword with the same orthographic form as an English word is italicized.

Word	Nonword	Freq. bin	Length
dante	zunte	low	5
limbo	fombo	low	5
magma	mazga	low	5
panda	marda	low	5
cantor	mandor	low	6
postal	montal	low	6
retina	devica	low	6
nominal	nomiton	low	7
soprano	robrino	low	7
vaginal	tavinas	low	7
cardinal	cansanal	low	8

Continued on next page

[4] As described in footnote 3, our stimulus selection process used a different method of converting to phonological forms than our final analysis. The stimulus file contains the phonogical forms, phonotactic scores, and phonemic lengths derived from both the new method and the original method.

Table 4 – continued from previous page

Real word	Nonword	Freq. bin	length
marginal	malvanal	low	8
pastoral	poncoral	low	8
placenta	claranta	low	8
vascular	vertular	low	8
comer	cogir	mid	5
cruel	cruer	mid	5
india	intio	mid	5
moral	pocal	mid	5
mover	mobor	mid	5
vigor	legor	mid	5
animal	amazal	mid	6
corona	coloto	mid	6
fiscal	vircal	mid	6
formal	vermal	mid	6
indias	intios	mid	6
portal	martal	mid	6
arsenal	algenas	mid	7
academia	amamegio	mid	8
circular	cusgular	mid	8
conceder	carcedir	mid	8
culpable	cuarible	mid	8
festival	lestañal	mid	8
judicial	nuvicias	mid	8
peculiar	perusior	mid	8
tropical	prosacal	mid	8
local	roral	high	5
negro	gedro	high	5
plaza	blava	high	5
porque	*mosque*	high	5
radio	samio	high	5
crisis	brimis	high	6
debate	redite	high	6
primer	clicer	high	6
sector	ructor	high	6
central	cistral	high	7
control	confrel	high	7
embargo	emporzo	high	7
general	nereras	high	7
natural	natutes	high	7
anterior	anceniar	high	8
director	dicortor	high	8
especial	encesial	high	8
exterior	eztetiar	high	8

Continued on next page

Table 4 – continued from previous page

Real word	Nonword	Freq. bin	length
interior	imperier	high	8
original	osipicaz	high	8
superior	sugetiar	high	8
victoria	veltorio	high	8

In addition, because we used different phonological forms for stimulus generation and analysis (see Section 4.1), not all stimuli ended up meeting our original inclusion criteria. To account for this, we re-evaluated the stimuli with respect to these criteria at the analysis phase, and decided to exclude a small number of additional stimuli in order to meet the level of control that was the target of our original experimental design. We excluded from the analysis 5 word-nonword pairs that were no longer matched for length according to the revised phonological forms; these pairs are shown in Table 5. We also excluded 11 word-nonword pairs that were no longer closely matched for phonotactic score, as indicated by having a word-nonword phonotactic score difference of greater than 0.15 in absolute value[5]; these pairs are shown in Table 6.

[5] Our original threshold for word-nonword phonotactic score difference was 0.1, but inspection of the distribution of score differences showed that this was not a particularly extreme value, so we extended it to 0.15.

Table 3: Number of word-nonword stimulus pairs observed by a participant in Experiment 1, broken down by length and frequency bin

Freq. bin		Length	Pairs
Low		5	8
		6	12
		7	12
		8	8
	Total		40
Mid		5	8
		6	12
		7	12
		8	8
	Total		40
High		5	8
		6	12
		7	12
		8	8
	Total		40
	Grand total		120

Table 5: Excluded stimulus pairs where the word and nonword no longer match for phonological length under the revised phonological forms. For each pair, the word or nonword whose length no longer matches the original value (given in the Length column) is italicized.

Word	Nonword	Freq. bin	Length
empresa	*extrela*	high	7
escena	enreca	mid	6
ascenso	almenco	mid	7
pretexto	tretalto	mid	8
contexto	canticto	mid	8

Table 6: Excluded stimulus pairs where the word and nonword no longer match closely enough for phonotactic score under the revised phonological forms (i.e. no longer have a word-nonword phonotactic score difference of less than 0.15 in absolute value).

Word	Nonword	Freq. bin	Length	Score diff.
banda	ranga	high	5	0.338
ámbito	ágnato	high	6	0.228
síndrome	díngrome	mid	8	0.227
miembro	mienglo	high	7	0.212
octubre	olcucre	high	7	0.181
bandido	venvido	low	7	0.178
monje	menve	mid	5	0.173
siempre	siamble	high	7	0.163
período	recíoda	high	7	0.160
monja	morba	low	5	−0.160
rango	rarno	mid	5	−0.386

2.4. Experiment 2

In Experiment 2, each participant responds to 240 nonwords, consisting of 48 nonwords of phoneme length 5, 72 nonwords of length 6, 72 nonwords of length 7, and 48 nonwords of length 8. The nonwords are randomly sampled from the larger pool of 510 nonword stimuli, without regard to their matched words or frequency bin.

After running the experiment, we realized that a single nonword with the same orthographic form as an English word (*mosque*, phonemic length 5) had made it into our pool. We excluded responses to this stimulus from the analysis.

In addition, because we used different phonological forms for stimulus generation and analysis (see Section 4.1), not all stimuli ended up meeting our original inclusion criteria. To account for this, we re-evaluated the stimuli with respect to these criteria at the analysis phase, and decided to also exclude the nonword *gedro* (length 5), which had an extreme (outlier) phonotactic score of lower than -1.3.

3. Procedure

In both experiments, participants begin with a pre-screening questionnaire (Section 6.1) and end with a post-task questionnaire (Section 6.2). Here, we describe the main task in-between these questionnaires for each experiment.

Both experiments present stimuli in written form, rather than in audio form, in order to ensure that participants perceive them as intended. The 240 critical trials are presented in random order.

Both experiments also include six attention check trials, interspersed evenly with critical trials. In an attention check, a prompt such as "Click the rightmost button" is shown, and participants must respond by clicking the indicated button among an array of radio buttons. Two of the attention check trials ask participants to click the rightmost button, two ask them to click the leftmost button, and two ask them to click the middle button; these trials are randomly shuffled among the slots for attention check trials. If a participant fails more than one attention check trial, the experiment ends prematurely and they do not receive payment.

3.1. Experiment 1

Experiment 1 is a Word Identification task. On each critical trial, the participant is shown the orthographic form of a stimulus (word or nonword) in large font at the top-center of the screen. Underneath the stimulus, there is an array of 5 radio buttons, laid out horizontally. At the left edge of the array is the label "Confident that this is NOT a Spanish word", where the words *is NOT* are presented on their own line, enlarged and colored red. At the right edge is the label "Confident that this IS a Spanish word", where the word *IS* is presented on its own line, enlarged and colored green. The participant is instructed to click a button on the scale, indicating their degree of confidence that the stimulus is a real Spanish word. 200 milliseconds after the participant clicks a button, the display clears, and a new stimulus appears after a further 300 millisecond pause.

The instructions presented to the participant at the beginning of the experiment are as follows:

> On each trial of the experiment, you will see text in large font in the middle of the screen, like in the image below.

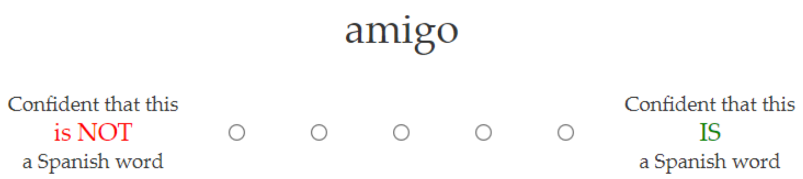

The text will be either a **real** Spanish word or a **made-up** Spanish word.

You will rate how confident you are that the text is a **real** Spanish word, by clicking on one of 5 circular buttons under the text.

As as you click one of the buttons, the experiment will automatically advance to the next trial. Please be wary of accidentally clicking a button you did not mean to choose.

Trust your first instincts, and **please do not look up the text in a dictionary or ask anyone else for help**. We are interested in what **you** know about Spanish, even if you are not certain of that knowledge.

IMPORTANT: Some trials of the experiment will look different, and will feature an English sentence. These trials are **attention checks**, and the sentences are instructions. In order to receive payment for your HIT, follow the instructions in attention checks carefully.

3.2. Experiment 2

Experiment 2 is a Wellformedness Rating task. The setup is identical to Experiment 1, except participants are informed that the stimuli are nonwords, and are asked to rate each nonword for how Spanish-like they perceive it to be. The label at the left edge of the radio button array is "HARDLY Spanish-like" and the label at the right edge is "VERY Spanish-like", where the words *HARDLY* and *VERY* are each presented on their own line, enlarged and colored red and green, respectively.

The instructions presented to the participant at the beginning of the experiment are as follows:

On each trial of the experiment, you will see **a made-up word** in large font in the middle of the screen, like in the image below.

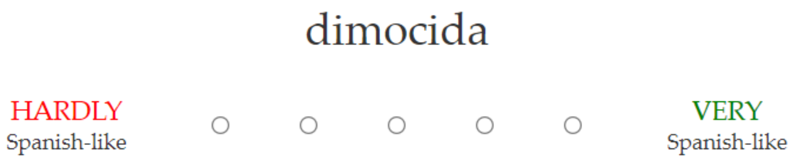

You will rate how **Spanish-like** the made-up word seems; that is, how good you think it would be as a Spanish word. For example, a rating for *dimocida* (a very Spanish-like made-up word) would be higher than a rating for *iocojea*

(a hardly Spanish-like made-up word).

You will give your rating by clicking on one of 5 circular buttons under the text. As as you click one of the buttons, the experiment will automatically advance to the next trial. Please be wary of accidentally clicking a button you did not mean to choose.

Trust your first instincts, and **please do not ask anyone else for help**. We are interested in what you know about Spanish, even if you are not certain of that knowledge.

IMPORTANT: Some trials of the experiment will look different, and will feature an English sentence. These trials are **attention checks**, and the sentences are instructions. In order to receive payment for your HIT, follow the instructions in attention checks carefully.

4. Phonological forms and phonotactic models

A key component of the experimental design is the manipulation of phonotactic probability. In this section, we describe the process by which orthographic stimuli were converted to phonological form, and the process by which phonotactic probability scores were calculated for these forms.

4.1. Conversion to phonological form

In order to calculate phonotactic scores, experimental stimuli and phonotactic model training data must be converted from orthographic to phonological form. To accomplish this, we used a series of rewrite rules. We originally created our own rewrite rules, which we used to select stimuli; however, we discovered some deficiencies in our rules after the experiment had been conducted. To account for this, we adopted the rule-based approach of eSpeak NG (version 1.52-dev), with some minor modifications, to use in our analysis. We describe only this revised version here.

We first converted words to IPA, with a space between each phoneme symbol, using the following eSpeak NG command:

```
cat <INPUT> | espeak-ng -qx -ves-419 -ipa -sep=" " -phonout <OUTPUT>
```

In this conversion, we used the set of rewrite rules that eSpeak NG designates for Latin American Spanish (voice `es-419`), under the assumption that Latin American varieties would constitute the majority of Spanish to which participants in our experiments are exposed.[6] Because eSpeak NG is a text-to-speech engine, the resulting phonological forms are represented at the surface allophone level rather than a deeper phoneme level.

From these IPA transcriptions, we removed stress-marking symbols[7], /ˈ/ and /ˌ/, as well as the length marker /ː/, which eSpeak NG had inserted for orthographic ⟨ss⟩ (/sː/), for orthographic ⟨ll⟩

[6]Mexicans account for more than 60% of self-identification as Latinx in 2017 according to the Pew Research Center tabulations of the 2017 American Community Survey.

[7]There exist minimal pairs in Spanish distinguished only by stress (which is orthographically marked when unpredictable). Our removal of stress-marking symbols can thus cause the neutralization of minimal pairs, giving rise to duplicate phonological forms in word lists. Though we do not represent stress in our phonological forms, we

(/jː/, which eSpeak NG wrote out as /j j/), and for /p/ before /t/. Finally, we converted each IPA phoneme symbol to ASCII characters, as shown in Table 7. Note that we converted rising diphthongs to two characters (where /ɪ/ became j and /ʊ/ became w), for consistency with eSpeak NG's treatment of lowering diphthongs and /oʊ/ (which eSpeak NG writes as /o w/).

Table 7: Mapping from IPA symbols to ASCII characters used in the conversion to phonological form

IPA	ASCII	IPA	ASCII
/p/	p	/b/	b
/t/	t	/d/	d
/tʃ/	C	/ɟ/	J
/k/	k	/g/	g
/f/	f	/β/	B
/s/	s	/ð/	D
/x/	x	/ɣ/	G
/ʃ/	S		
/m/	m	/ɾ/	R
/n/	n	/r/	r
/ɲ/	Y	/l/	l
/ŋ/	N		
/w/	w	/j/	j
/a/	a	/aɪ/	a j
/ɛ/	e	/eɪ/	e j
/i/	i	/oɪ/	o j
/o/	o	/aʊ/	a w
/u/	u	/eʊ/	e w

The full process of converting orthographic to phonological forms is accomplished by the script `orth2phon/spanish-orth2phon.sh` in the OSF repository associated with these supplementary materials.

Of course, alternative treatments of phonological form conversion are possible, such as conversions that are represented at a deeper phoneme level rather than the surface allophone level, conversions that maintain stress or length distinctions, or conversions that treat diphthongs differently. Since each treatment corresponds to an assumption about the way in which non-Spanish speakers perceive and represent Spanish sounds, it is an empirical question which one is most appropriate. A Wellformedness Rating task could be used to compare various treatments, analogously to the way in which Oh, Todd, Beckner, Hay, King & Needle (2020) determined that it was appropriate to collapse vowel length distinctions in Māori. Since there are many possible alternatives, we leave thorough investigation of them for future work.

refrain from merging duplicate phonological forms that result from this lack of representation; in this way, each lexical item maintains its own entry. This approach can be justified on the basis of results from Ortín & Simonet (2023), which suggest that English-speaking listeners are able to hear the differences between members of a stress-based minimal pair in Spanish, even if they do not represent this difference in memory due to its peripheral role in English phonology.

4.2. Phonotactic model training data

4.2.1. SUBTLEX-ESP

The phonotactic model used for stimulus generation and selection, as well as most of the analyses, was trained on word types in SUBTLEX-ESP (Cuetos, González-Nosti, Barbón & Brysbaert, 2011). SUBTLEX-ESP is a word frequency database constructed from a corpus of 41 million words from Spanish subtitles of film and TV series that aired between 1990 and 2009. The database contains 94,344 word types. Unlike SPALEX, from which we obtained potential real word stimuli (Section 2.1.1), SUBTLEX-ESP does not control for morphological complexity. It is a reflection of Spanish as it may be overheard, containing proper nouns, compounds, and inflected forms.

To prepare the SUBTLEX-ESP word list for phonotactic modeling, we converted Spanish orthographic forms to phonological forms following the procedure described in Section 4.1. We then removed loan words that don't conform to categorical Spanish phonotactics, and we combined duplicate entries with the same orthographic form (adding together their counts from the database). This left us with 93,777 distinct Spanish word types.

The list of word types from SUBTLEX-ESP are available in the supplementary file `monte-carlo/words-and-counts.txt` in the OSF repository associated with these supplementary materials. For each word type, we note the orthographic form and the phonological form, as well as its frequency (count in the database).[8]

4.2.2. Morphs

Following Oh et al. (2020), we also consider the possibility that participants' phonotactic knowledge could be built over units smaller than words, i.e. *morphs*.

To segment words into morphs, we used the Morfessor Baseline algorithm (Creutz & Lagus, 2002), as implemented in the Morfessor 2.0 Python package (Virpioja, Smit, Grönroos & Kurimo, 2013). Morfessor uses an unsupervised machine learning approach to identify morphs that recur with statistical regularity across words, mirroring what might be expected from a listener exposed to a language that they do not speak. Note that Morfessor assumes a strictly concatenative model of morphology, which means that it is unable to reverse-engineer phonologically-conditioned allomorphy, and thus is likely to arrive at an inflated and relatively surface-level morph inventory involving redundant listings of each such allomorph. Indeed, manual inspection of Morfessor's segmentations highlights that they often do not align with expectations from Spanish grammars or intuitions of native speakers. We do not view this as particularly problematic, as we are not attempting to model Spanish grammar or native speaker intuition; rather, we are attempting to model the representations that non-Spanish speakers may form upon repeated incidental exposure to Spanish, which are likely to be relatively surface-level and imprecise.

We trained the model on the phonological forms of the 93,777 word types from SUBTLEX-ESP (ignoring frequency), using the default parameter settings (and with random seed set to 1234). This gave us a set of 12,773 morphs.

We calculated the frequency (count) of each morph based on the SUBTLEX-ESP frequencies of the words within which it occurs. That is, for each morph, we used the Morfessor segmentations to identify the words from SUBTLEX-ESP that it occurs in, and we added together the frequencies of those words.

[8] The phonological forms used in this file are derived from the revised method of phonological conversion used for the analysis, rather than the original one used for stimulus selection (see Section 4.1). Thus, phonotactic scores generated from this file will match those used in the analysis, but not those originally used for stimulus selection.

The list of morphs and their frequencies is available in the supplementary file `monte-carlo/morphs-and-counts.txt` in the OSF repository associated with these supplementary materials.[9]

4.3. Phonotactic scoring

Phonotactic scores were calculated using length-normalized log-probabilities, which were generated with the SRI Language Modeling Toolkit (SRILM) (Stolcke, 2002). We trained a Witten-Bell-smoothed trigram language model over phonemes, based on the phonological forms of (isolated) words and morphs from SUBTLEX-ESP.

The model used to generate phonotactic scores measures the probability of generating or predicting each phoneme in turn, as well as the end-of-word symbol. Accordingly, the phonotactic score of a stimulus of phoneme length L is calculated by dividing its model-provided log-probability by $(L + 1)$.

Our methods follow those of Oh et al. (2020). For more description, including the intuition behind the models, please see their Detailed Materials and Methods Supplement, Section 4.2.2–4.2.3.

4.3.1. Assuming stimuli are not parsed into morphs

In the generation and selection of stimuli, as well as most of the analysis, we use phonotactic scores that assume participants have a proto-lexicon composed of words. To derive these phonotactic scores, we train a model on the phonological forms of word types in SPALEX.

The SRILM command used to train the model is as follows (where the types in the input are composed of individual characters representing phonemes, separated by whitespace):

```
ngram-count -text <INPUT> -lm <OUTPUT> -order 3 -wbdiscount -interpolate
```

The SRILM command used to derive phonotactic probabilities for the stimuli is as follows (where each stimulus is composed of individual characters representing phonemes, separated by whitespace):

```
ngram -lm <MODEL> -ppl <STIMULI> -debug 1 > <OUTPUT>
```

In the analysis of Experiment 2, we also use phonotactic scores that assume that participants have a proto-lexicon composed of morphs, but do not parse stimuli into morphs (i.e. stimuli are treated as morphologically simplex). We derive these phonotactic scores in the same way as the word-based scores above, except we train the model on the phonological forms of morph types rather than word types.

The full process of calculating phonotactic scores that assume stimuli are not parsed into morphs is accomplished by the script `phonotactics/score-unparsed.sh` in the OSF repository associated with these supplementary materials.

[9] The phonological forms used in this file are derived from the revised method of phonological conversion used for the analysis, rather than the original one used for stimulus selection (see Section 4.1).

4.3.2. Assuming stimuli are parsed into morphs

Our analysis of Experiment 2 also uses a third phonotactic score, which assumes that participants have a proto-lexicon composed of morphs, and that they may parse stimuli into morphs (i.e. that stimuli are treated as potentially morphologically complex). These scores must be derived in a different way.

The model is trained on morph types in the same way as described in Section 4.3.1; however, we make two small tweaks to the output model file. First, we replace the start symbol "<s>" and end symbol "</s>" with the morph boundary symbol "+", to enable a sequence of morphs to be generated. Second, we add a bigram with a log-probability of -99, to prevent a morph boundary from being generated immediately after another boundary.

The model is applied in different way, as a language model with hidden events (Stolcke & Shriberg, 1996) rather than a plain language model. The SRILM command used to derive morph-based phonotactic probabilities for the stimuli, assuming participants are parsing stimuli into morphs, is as follows (where each stimulus is composed of individual characters representing phonemes, plus an initial and final morph boundary "+", all separated by whitespace, and where the file `-hidden-vocab morph-boundary.txt` contains only the morph boundary symbol, "+"):

```
ngram -lm <MODEL> -ppl <STIMULI> -debug 2 -hidden-vocab morph-boundary.txt
-no-eos -no-sos > <OUTPUT>
```

The phonotactic probability derived in this way includes a component for generating an initial morph boundary symbol "+", which is undesirable. We remove this component by subtracting the unigram log-probability of generating the initial morph boundary from the log-probability of the stimulus. We then calculate the phonotactic score by dividing the log-probability by the number of phonemes, plus one, as described in Section 4.3.

The full process of calculating phonotactic scores that assume stimuli are parsed into morphs is accomplished by the script `phontactics/score-parsed.sh` in the OSF repository associated with these supplementary materials.

5. Statistical analysis

5.1. Ordinal regression

For all the statistical analyses performed in the study, we use (logit) ordinal regression, as implemented in the functions `clm` (fixed-effects only) and `clmm` (mixed-effects) from the *R* package `ordinal` (Christensen, 2020). (Logit) Ordinal regression can be seen as an extension of logistic regression that models the relationship between responses on a discretized scale and one or more explanatory variables (Liddell & Kruschke, 2018). For a description of the underlying ideas, see Section 5.1 of the Detailed Materials and Methods Supplement of Oh et al. (2020).

In all regression models using phonotactic scores, we include an additional binary predictor for the presence or absence of non-English characters (together with its own by-participant random slope in mixed effects models). We make this distinction to account for the fact that the visual presence of non-English characters (i.e. accented characters) may prompt a high "Spanish-ness" judgment, regardless of phonotactics.

When plotting the partial effects of certain predictors in ordinal regression results, we present the expected value of the rating, using the method described in Section 5.1 of the Detailed Materials and Methods Supplement of Oh et al. (2020).

5.2. Model comparison with AIC

To analyze the results of Experiment 2, we perform a series of comparisons of different ordinal regression models (fitted on the same data). Each model is based on different assumptions about participants' phonotactic knowledge, encapsulated in the use of alternative phonotactic scores. We compare models using the AIC score (Burnham & Anderson, 2004; Wagenmakers & Farrell, 2004), where better models have lower AIC.

When comparing models with AIC, it is not the absolute AIC scores that matter, but rather the magnitude of their difference. The difference in the AIC scores of two models relates to the relative strength of the evidence for one model over the other in probabilistic terms (Burnham & Anderson, 2004; Wagenmakers & Farrell, 2004). When comparing models, a standard rule-of-thumb states that a difference of less than 2 AIC points indicates that they perform similarly (well or poorly), while a difference of 10 AIC points or more indicates that the model(s) with higher AIC performs worse than the model(s) with lower AIC (Wagenmakers & Farrell, 2004).

5.3. Monte Carlo analyses

In order to assess the size of the proto-lexicon that permits best explanation of the NSS' behaviorally-observed phonotactic knowledge, we use Monte Carlo methods. We train a host of phonotactic models on different-sized subsets of the data (in words or morphs), use the resultant phonotactic scores in (logit) ordinal regression models of participants' wellformedness ratings in Experiment 2, and collate the AIC scores of these regression models for comparison. This approach lets us (implicitly) perform model selection over phonotactic models with different-sized sets.

Our Monte Carlo methods use random sampling to form a distribution over the performance of ordinal regression models (as quantified by their AIC scores) relative to each proto-lexicon size. This is a computationally intense procedure, so we use fixed-effects ordinal regression (`clm`) rather than mixed-effects ordinal regression (`clmm`). Although we naturally expect our quantitative results to be affected by such a simplification, we do not expect it to have a substantial effect qualitatively.

For each proto-lexicon size N, we perform the following steps 1,000 times:

1. Sample N types (words or morphs) from the training data.
2. Train a phonotactic model on these types and calculate phonotactic scores for the stimuli, using the appropriate configuration from Section 4.3.
3. Run an ordinal regression predicting participant ratings from phonotactic score and the presence of non-English characters, and extract the AIC value.

We adopt two different sampling schemes to account for different ways of forming a proto-lexicon of a given size. Each sampling scheme subsamples N types from the full set of T types, without replacement, thus yielding different probability distributions over the set of possible proto-lexicons.

- Unweighted: samples types uniformly at random.
  - The probability of the ith th sample S_i yielding an as-yet unsampled type y is: $P(S_i = t) = 1/\,(T - i + 1)$.
  - Here, all proto-lexicons are equally likely. In other words, this scheme assumes that proto-lexicon formation is not influenced by experiential statistics, i.e. by the frequency with which different units are experienced in ambient exposure.
- Frequency-weighted: samples types proportional to their frequency.

– The probability of the ith sample S_i yielding an as-yet unsampled type t of frequency $f(t)$ is: $P(S_i = t) = f(t)/\Sigma_{\{x:x \notin S\}} f(x)$.
– Here, proto-lexicons containing high-frequency types are more likely. This means that proto-lexicon formation is highly sensitive to experiential statistics: every encounter with a type represents an opportunity to add it to the proto-lexicon. Taking into account individual-level variation in experiential statistics, this sensitivity could lead different individuals to form considerably different proto-lexicons, with different low-frequency types.

Since the Monte Carlo analysis only uses fixed-effects regression models, the results may be overly influenced by particular participants or stimuli. In order to account for this possibility, and to obtain a more informative sense of the best proto-lexicon size, we develop a single representative phonotactic score system for each size, which we can compare using mixed-effects regression.

We base our representative phonotactic scores on the frequency-weighted sampling scheme. To collapse the 1000 different phonotactic score systems at each vocabulary size into a single score system, we convert the 1000 scores for each stimulus to probabilities (via exponentiation), take the means of these probabilities, and then convert them back to scores (via log-transformation). The resultant scores represent an aggregate-level average of a situation in which participants may all possess slightly different proto-lexicons of the same size, taking into account the probability of a participant having a particular proto-lexicon.

We separately compare mixed-effect regression models using representative phonotactic scores based on different proto-lexicon sizes, to get another perspective on which proto-lexicon size may be best.

5.4. Attitude scoring

To assess how participants' formation of a proto-lexicon may be affected by their attitudes toward Spanish and its speakers – both within their state and at the national level – we included questionnaire responses in the regression model.

The post-task questionnaire included 4 sub-questions about explicit attitudes toward Spanish and its speakers (see Section 6.2). Two sub-questions asked about the value the participant places on Spanish language and culture in their state, while the other two asked about nationalist attitudes relating to immigration and the role of English in the US. We created two attitude variables from participants' responses to these sub-questions, as follows:

- *Spanish value*: a measure of how strongly the participant is in favor of Spanish language and culture in their home state. This measure is obtained by quantifying the responses to questions (16a) and (16b) on a 5-point scale, adding them together, and subtracting 6. The results can range from -4 to 4, with positive numbers indicating a positive attitude toward Spanish.

- *Nationalism*: a measure of the strength of the participant's nationalist feelings toward the US. This measure is obtained by quantifying the responses to questions (16c) and (16d) on a 5-point scale and subtracting the response to (16c) from the response to (16d). The results can range from -4 to 4, with positive numbers indicating a nationalist attitude toward the US.

5.5. *Exposure*

In a similar manner as for attitude, we created a variable which represents the level of the participant's overall exposure to Spanish, based on post-task questions about their frequency of exposure through media and social contact (see Section 6.2). This variable was obtained by quantifying the participant's responses to questions (14) (media exposure) and (15) (social exposure) on a 5-point scale and adding them together. The resultant scores can range from 2 to 10, and can be seen as a log-like estimate of the level of exposure for each participant.

6. Questionnaires

6.1. *Pre-screening questionnaire*

In each experiment, participants had to answer a pre-screening questionnaire to determine participation eligibility. The pre-screening questionnaire contained the following questions:

1. What is your native language? (This is the language that you learned and used most before the age of 7)
   In order to start the experiment, participants had to select *English*.

2. In which country did you learn English?
   In order to start the experiment, participants had to select *United States of America*.

3. Where are you currently located?
   In order to start the experiment, participants had to select either *California* or *Texas*. (Options listed all US states, as well as *Outside of the US*.)

4. What is the longest period of time you have lived outside of this state[10] since the age of 7?
   In order to start the experiment, participants had to select any period of time less than 1 year, which includes the options *I have not lived outside of this state*, *1 month or less*, *1-3 months*, *3-6 months*, and *6 months - 1 year*.

5. Have you ever taken a college-level course in Linguistics?
   In order to start the experiment, participants had to select *No*.

6. Can you hold a basic conversation in Spanish?
   In order to start the experiment, participants had to select *No*.

Our pre-screening questionnaire also automatically estimated the participant's geolocation based on their IP address. We did not screen based on this variable, due to reports from participants that it may not have been accurate in some cases. Nevertheless, we retain the automated geolocation information in the dataset distributed with this paper.

[10] The word *California* or *Texas* was substituted into pre-screening question 4, based on the participant's response to pre-screening question 3.

6.2. Post-task questionnaire

After completing an experiment, participants answered the following post-task questionnaire:

1. Which age group do you belong to?
   - 18-29
   - 30-39
   - 40-49
   - 50-59
   - 60+
2. Please state your gender.
3. Please state your ethnicity.
4. Which political party do you identify with most strongly?
   - Democratic party
   - Republican party
   - Other (Please specify)
   - None
5. What is your highest level of education?
   - Some grade school
   - 8th grade
   - High school diploma / GED
   - 2-year college degree
   - 4-year college degree
   - Graduate-level college degree (Master's or Doctorate)
6. Please list any languages other than English that you can speak well.
7. Do you speak/understand any Romance languages such as French, Italian, Portuguese, or Romanian, even at a basic level?
   - Yes (Please specify)
   - No
8. Have you ever lived in a country outside the US where Spanish is predominantly spoken?
   - Yes (Please specify)
   - No
9. Have you ever lived in a country outside the US where a Romance language (such as French, Italian, Portuguese, or Romanian) is predominantly spoken?
   - Yes (Please specify)
   - No
10. What is the highest level of education at which you have studied Spanish?

- At elementary school
- At high school
- At undergraduate level at college, or at community college
- At graduate level at college
- Never studied it

11. How well are you able to speak Spanish?

    - Very well (I can talk about almost anything in Spanish)
    - Well (I can talk about many things in Spanish)
    - Fairly well (I can talk about some things in Spanish)
    - Not very well (I can only talk about simple/basic thinks in Spanish)
    - No more than a few words or phrases
    - Not at all

12. How well are you able to understand/read Spanish?

    - Very well (I can understand almost anything said/written in Spanish)
    - Well (I can understand many things said/written in Spanish)
    - Fairly well (I can understand some things said/written in Spanish)
    - Not very well (I can only understand simple/basic things said/written in Spanish)
    - No more than a few words or phrases
    - Not at all

13. Please check all of the following that you know how to say in Spanish.

    - Basic phrases (e.g. My name is... / I'm from...)
    - Questions (e.g. What is your name? / Where are you from?)
    - Commands (e.g. Sit down / Come here)
    - Greetings (e.g. Hello / How are you?)
    - Numbers up to 10
    - Numbers above 10
    - Body parts
    - Colors

14. How often do you think you are exposed to the Spanish language in your daily life, by means of media such as Spanish radio, Spanish TV, online media, etc.?

    - About once a year, or less
    - About once a month, or less
    - About once a week, or less
    - About once a day, or less
    - Multiple times a day

15. How often do you think you are exposed to the Spanish language in your daily life, in conversation at work, at home, or in social settings?

    - About once a year, or less
    - About once a month, or less
    - About once a week, or less

- About once a day, or less
- Multiple times a day

16. How do you feel about each of the following statements?

    a. Some Spanish language education should be compulsory in school for all children in California/Texas.
        - Strongly agree
        - Somewhat agree
        - Neither agree nor disagree
        - Somewhat disagree
        - Strongly disagree

    b. Hispanic and Latino cultures are important in California/Texas.
        - Strongly agree
        - Somewhat agree
        - Neither agree nor disagree
        - Somewhat disagree
        - Strongly disagree

    c. People in the United States should speak English, not foreign languages.
        - Strongly agree
        - Somewhat agree
        - Neither agree nor disagree
        - Somewhat disagree
        - Strongly disagree

    d. How would you complete the following statement?
    I think that the number of immigrants from foreign countries who are permitted to come to the United States to live should be _____.
        - Increased a lot
        - Increased a little
        - Left the same
        - Decreased a little
        - Decreased a lot

17. Any general comments about the experiment? Did you run into any issues? [optional]

References

Aguasvivas, J. A., Carreiras, M., Brysbaert, M., Mandera, P., Keuleers, E., & Duñabeitia, J. A. (2018). SPALEX: A Spanish lexical decision database from a massive online data collection. *Frontiers in Psychology*, *9*, 2156. doi:10.3389/fpsyg.2018.02156.

Brysbaert, M., Mandera, P., & Keuleers, E. (2018). The word frequency effect in word processing: An updated review. *Current Directions in Psychological Science*, *27*, 45–50. doi:10.1177/0963721417727521.

Burnham, K. P., & Anderson, D. R. (2004). Multimodel inference: Understanding AIC and BIC in model selection. *Sociological Methods & Research*, *33*, 261–304. doi:10.1177/0049124104268644.

Christensen, R. H. B. (2020). ordinal—Regression models for ordinal data [R package installed from source dated 8/22/2022]. URL: https://github.com/runehaubo/ordinal.

Creutz, M., & Lagus, K. (2002). Unsupervised discovery of morphemes. In *Proceedings of the ACL-02 Workshop on Morphological and Phonological Learning* (pp. 21–30). Association for Computational Linguistics. doi:10.3115/1118647.1118650.

Cuetos, F., González-Nosti, M., Barbón, A., & Brysbaert, M. (2011). SUBTLEX-ESP: Spanish word frequencies based on film subtitles. *Psicológica*, *32*, 133–143.

Duchon, A., Perea, M., Sebastián-Gallés, N., Martí, A., & Carreiras, M. (2013). EsPal: One-stop shopping for Spanish word properties. *Behavior Research Methods*, *45*, 1246–1258. doi:10.3758/s13428-013-0326-1.

Keuleers, E., & Brysbaert, M. (2010). Wuggy: A multilingual pseudoword generator. *Behavior Research Methods*, *42*, 627–633. doi:10.3758/BRM.42.3.627.

Liddell, T. M., & Kruschke, J. K. (2018). Analyzing ordinal data with metric models: What could possibly go wrong? *Journal of Experimental Social Psychology*, *79*, 328–348. doi:10.1016/j.jesp.2018.08.009.

Lin, J. (1991). Divergence measures based on the Shannon entropy. *IEEE Transactions on Information Theory*, *37*, 145–151. doi:10.1109/18.61115.

Oh, Y., Todd, S., Beckner, C., Hay, J., King, J., & Needle, J. (2020). Non-Māori-speaking New Zealanders have a Māori proto-lexicon. *Scientific Reports*, *10*, 22318. doi:10.1038/s41598-020-78810-4.

Ortín, R., & Simonet, M. (2023). Perceptual sensitivity to stress in native English speakers learning Spanish as a second language. *Laboratory Phonology*, *14*. doi:10.16995/labphon.7978.

Stolcke, A. (2002). SRILM - an extensible language modeling toolkit. In J. H. L. Hansen, & B. Pellom (Eds.), *Proceedings of the Seventh International Conference on Spoken Language Processing* (pp. 901–904).

Stolcke, A., & Shriberg, E. (1996). Automatic linguistic segmentation of conversational speech. In H. T. Bunnell, & W. Idsardi (Eds.), *Proceeding of the Fourth International Conference on Spoken Language Processing* (pp. 1005–1008).

Virpioja, S., Smit, P., Grönroos, S.-A., & Kurimo, M. (2013). *Morfessor 2.0: Python Implementation and Extensions for Morfessor Baseline*. Technical Report Department of Signal Processing and Acoustics, Aalto University Helsinki.

Wagenmakers, E.-J., & Farrell, S. (2004). AIC model selection using Akaike weights. *Psychonomic Bulletin & Review*, *11*, 192–196. doi:10.3758/BF03206482.
