# Supplementary material for: Language structure, attitudes, and learning from ambient exposure: Lexical and phonotactic knowledge of Spanish among non-Spanish-speaking Californians and Texans
Source: PLoS One. 2023 Apr 27;18(4):e0284919. doi: 10.1371/journal.pone.0284919 (PMC10138781; doi:10.1371/journal.pone.0284919)
Supplement: S2 File — This file contains all the code used for data exclusion, analysis, and plotting. It describes each step of the analysis and includes all statistical results (e.g. model summaries). (HTML) [file pone.0284919.s002.html]

Language structure, attitudes, and learning from ambient exposure: Lexical and phonotactic knowledge of Spanish among non-Spanish-speaking Californians and Texans


Code 

- Show All Code
- Hide All Code

# Language structure, attitudes, and learning from ambient exposure: Lexical and phonotactic knowledge of Spanish among non-Spanish-speaking Californians and Texans

### Detailed Analysis and Results Supplement

#### Simon Todd, Chadi Ben Youssef, and Alonso Vásquez-Aguilar

#### University of California, Santa Barbara

#### April 13, 2023

```
# Setup -----

library(matrixStats)
library(knitr)
library(tidyverse)
library(ordinal)
library(effects)
library(MASS)
library(kableExtra)
library(egg)
library(purrr)
library(grid)
library(car)
library(polycor)
library(psych)
library(emmeans)

opts_chunk$set(echo=TRUE, message=FALSE, warning=FALSE, fig.show='hold', results='hold')
options(dplyr.summarise.inform = FALSE)
options(knitr.kable.NA = '')

# Local functions ----

# A function to center variables
c. <- function (x) scale(x, scale = FALSE)

# Custom function to format ordinal regression results in a nice table
clm_table <- function(mod, digits=3, ...) {
  table <- summary(mod)$coefficients %>%
    data.frame() %>%
    rlang::set_names(c("beta", "se", "z", "p")) %>%
    rownames_to_column("parameter") %>%
    mutate(
      parameter = parameter %>%
        str_replace_all(
          ., 
          str_c("(", str_c(names(attr(mod$terms, "dataClasses"))[attr(mod$terms, "dataClasses") %in% c("factor", "logical", "character")][-1], collapse="|"), ")"), 
          "\\1 = "
        ) %>%
        str_replace_all(
          .,
          "(?<=^|\\:)(?:c\\.\\()([^:]+)(?:\\))(?=$|\\:)",
          "\\1 (centered)"
        ) %>%
        str_replace_all(., fixed(":"), " &times; "),
      significance = case_when(
        p < 0.001 ~ "\\*\\*\\*",
        p < 0.01 ~ "\\*\\*",
        p < 0.05 ~ "\\*",
        p < 0.1 ~ ".",
        TRUE ~ ""
      ),
      is_threshold = parameter %in% names(mod$alpha)
    ) %>%
    mutate_at(c("z", "p", "significance"), ~ ifelse(is_threshold, NA, .)) %>%
    arrange(is_threshold) %>%
    mutate(
      type = c("Effects", rep("", n()-sum(is_threshold)-1), "Thresholds", rep("", sum(is_threshold)-1))
    ) %>%
    dplyr::select(type, parameter, beta, se, z, p, significance) %>%
    mutate(
      p = ifelse(p<0.001, "<0.001", format(round(p, 3), nsmall=3))
    ) %>%
    kable(digits=3, escape=F, col.names=c("", "Parameter", "Estimate", "Std. Error", "$z$", "$p$", ""), align="llrrrrl", ...) %>%
    column_spec(1, bold=TRUE) %>%
    kable_styling()
  return(table)
}

# Custom function to compute summary data for an ordinal regression model, to be plotted. The "type" argument specifies whether the data are for a latent variable plot, a distributional plot, or a mean rating plot. 
clm_plotdat = function(mod, focal.predictors, type="latent", response.coding=NULL, ...) {
  # For latent variable plot, draw straight from Effect
  if (type=="latent") {
    latentdat <-
      suppressWarnings(Effect(focal.predictors, mod, latent=TRUE, ...)) %>%
      as.data.frame() %>%
      dplyr::select(-se) %>%
      rename("pred"=fit, "lci"=lower, "uci"=upper)
    return(latentdat)
  }
  
  # Get distributional data (used for both of other plots)
  distdat <-
    suppressWarnings(Effect(focal.predictors, mod, ...)) %>%
    as_tibble() %>%
    dplyr::select(all_of(focal.predictors), matches("^(?:(?:L|U)\\.)?prob")) %>%
    pivot_longer(
      cols = -all_of(focal.predictors),
      names_to = c(".value", "response"),
      names_pattern = "^((?:(?:L|U)\\.)?prob)\\.(.+)$"      
    ) %>%
    rename("pred"=prob, "lci"=L.prob, "uci"=U.prob) %>%
    mutate(
      response = rep_len(mod$y.levels, n())
    )
  if (type=="dist") return(distdat)
  
  if (type=="mean") {
    # Get threshold values
    thresholds = c(-Inf, as.numeric(mod$alpha), Inf)
    
    # Get response coding
    if (is.null(response.coding)) {
      response.coding = 1:(length(mod$y.levels))
      names(response.coding) = mod$y.levels
    }
    
    # Build latent variable data based on distributional data
    # Note: this is a HACK since drawing latent variable data from Effect doesn't account for threshold variability. The confidence intervals assume fixed thresholds, which obscures patterns in variation of the thresholds; CIs are thus not correct (but are better than what would be obtained from direct transformation of the latent variable from Effect)
    
    # Get distribution-based latent variable data, assuming fixed thresholds
    latentdat <- distdat %>%
      group_by(across(all_of(focal.predictors))) %>%
      summarise(
        pred = qlogis(1-pred[1]) + thresholds[2],
        lci = qlogis(1-uci[1]) + thresholds[2],
        uci = qlogis(uci[length(uci)]) + thresholds[length(thresholds)-1]
      ) %>%
      as.data.frame()    
    
    # Get probabilities for each response option
    meandat <- latentdat
    for (i in 1:length(mod$y.levels)) {
      meandat[, paste0("(", mod$y.levels[i], ").prob")] = plogis(latentdat[, "pred"] - thresholds[i]) - plogis(latentdat[, "pred"] - thresholds[i+1])
      meandat[, paste0("(", mod$y.levels[i], ").lci")] = plogis(latentdat[, "lci"] - thresholds[i]) - plogis(latentdat[, "lci"] - thresholds[i+1])
      meandat[, paste0("(", mod$y.levels[i], ").uci")] = plogis(latentdat[, "uci"] - thresholds[i]) - plogis(latentdat[, "uci"] - thresholds[i+1])
    }
    meandat <- meandat %>%
      dplyr::select(-pred, -uci, -lci) %>%
      pivot_longer(
        cols = -all_of(focal.predictors),
        names_to = c("response", ".value"),
        names_pattern = "^\\((.+)\\)\\.([^.]+)$"
      ) %>%
      left_join(
        data.frame(response=names(response.coding), code=response.coding, stringsAsFactors=FALSE),
        by = "response"
      ) %>%
      group_by(across(all_of(focal.predictors))) %>%
      summarise(
        pred = sum(prob * code),
        lci = sum(lci * code),
        uci = sum(uci * code)
      ) %>%
      ungroup()
    return(meandat)
  }
}

# Custom function to get the information about thresholds, in a nice format for plotting
clm_thresholds = function(mod) {
  thresholds <- summary(mod)$coefficients %>%
  as.data.frame() %>%
  rownames_to_column() %>%
  dplyr::select(1:3) %>%
  set_names(c("label", "value", "se")) %>%
  filter(label %in% names(mod$alpha)) %>%
  mutate(
    lci = value - 1.96*se,
    uci = value + 1.96*se
  ) %>%
  dplyr::select(-se)
  return(thresholds)
}

# A function to add phonotactic scores from a file to the dataset
add_scores = function(data, scoreFile, scoreName) {
  scored_data <- data %>%
    left_join(
      read_csv(scoreFile, col_types=cols_only(item=col_character(),
                                              logprob=col_double())
               )%>%
        transmute(
          phon = item,
          !!scoreName := logprob / (nchar(item) + 1)
        ) %>%
        distinct()
      , by="phon"
    )
  return(scored_data)
}

# A function to get logMeanExp phonotactic scores from a range of files named sample_X.csv in a folder
logMeanExp_scores = function(path, numFiles) {
  filepath = paste0(path, "sample_1.csv")
  logprobs = read_csv(filepath, col_types=cols_only(item=col_character(),
                                                    logprob=col_double())) %>%
    mutate(
      sample = 1
    ) %>%
    distinct()
  if (numFiles > 1) {
    for (filenum in 2:numFiles) {
      filepath = paste0(path, "sample_", filenum, ".csv")
      logprobs = logprobs %>%
        bind_rows(
          read_csv(filepath, col_types=cols_only(item=col_character(),
                                                 logprob=col_double())) %>%
            mutate(
              sample = !!filenum
            ) %>%
            distinct()
        )
    }
  }
  scores = logprobs %>%
    group_by(item) %>%
    summarise(
      scoreAverage = (logSumExp(logprob) - log(!!numFiles))/ (nchar(first(item)) + 1)
    ) %>%
    ungroup() %>%
    rename("phon" = item)
  return(scores)
}

# A function to run shell scripts on Windows or UNIX-based OS
run_shell = function(cmd) {
  if (Sys.info()[['sysname']] == "Windows") {
    return(shell(cmd, flag="", ignore.stdout=TRUE, ignore.stderr=TRUE))
  } else {
    return(system(cmd, ignore.stdout=TRUE, ignore.stderr=TRUE))
  }
}

# A function to highlight the minimum value in a column, for presentation in a kable
# Highlighting is accomplished by coloring red
highlight_min = function(col, digits=1) {
  rounded <- round(col, digits)
  i <- which(rounded==min(rounded))
  highlighted <- cell_spec(format(rounded, nsmall=digits), align="r")
  highlighted[i] <- cell_spec(format(rounded[i], nsmall=digits), align="r", color="red")
  return(highlighted)
}

# A function to display a table of numbers
display_table = function(dat, caption, digits=3, highlight=c()) {
  table <- dat %>%
    mutate_at(highlight, ~ highlight_min(., digits=digits)) %>%
    kable(caption=caption, digits=digits, escape=F) %>%
    kable_styling()
  return(table)
}

# A function to make a bar plot of counts of levels of a factor in a dataframe
make_count_plot = function(data, varname, xlab, ylab, title) {
  if (is.numeric(data[, varname])) {
    min_val = min(data[, varname])
    max_val = max(data[, varname])
    data[, varname] = factor(data[, varname], levels = min_val:max_val)
    drop = FALSE
  } else {
    drop = TRUE
  }
  p = data %>%
    dplyr::count(across(all_of(varname)), .drop=drop) %>% 
    ggplot(., aes_string(x=varname, y="n", fill=varname)) +
      geom_col(show.legend=F) +
      geom_text(aes(label=n), vjust=-0.2) +
      labs(x=xlab, y=ylab) +
      scale_y_continuous(expand=expansion(add=c(0,15))) +
      theme_bw() +
      theme(
        panel.grid = element_blank()
        ) +
      ggtitle(title)
  
  return(p)
}

# A function to calculate VIFs for a clmm object and display them in a table
# NB: VIFs are calculated from a fixed-effect polr object
clm_vif = function(mod, dat, ...) {
  table <- vif(polr(mod$terms, data=dat, method="logistic", Hess=TRUE))
  # Check if VIFs or GVIFs have been calculated
  if (is.null(dim(table))) {
    table <- vif_table(table, ...)
  } else {
    table <- gvif_table(table, ...)
  }
  return(table)
}

# A function to neatly display a VIF table
vif_table = function(table, ...) {
  table = table %>%
    data.frame() %>%
    rlang::set_names(c("vif")) %>%
    rownames_to_column("parameter") %>%
    mutate(
      parameter = parameter %>%
        str_replace_all(
          .,
          "(?<=^|\\:)(?:c\\.\\()([^:]+)(?:\\))(?=$|\\:)",
          "\\1 (centered)"
        ) %>%
        str_replace_all(., fixed(":"), " &times; ")
    ) %>%
    kable(digits=3, escape=F, col.names=c("Parameter", "VIF"), align="lr", ...) %>%
    kable_styling()
  return(table)
}

# A function to neatly display a GVIF table
gvif_table = function(table, ...) {
  table = table %>%
    data.frame() %>%
    rlang::set_names(c("gvif", "df", "transformed")) %>%
    rownames_to_column("parameter") %>%
    mutate(
      parameter = parameter %>%
        str_replace_all(
          .,
          "(?<=^|\\:)(?:c\\.\\()([^:]+)(?:\\))(?=$|\\:)",
          "\\1 (centered)"
        ) %>%
        str_replace_all(., fixed(":"), " &times; "),
      transformed = transformed ^ 2
    ) %>%
    kable(digits=3, escape=F, col.names=c("Parameter", "GVIF", "df", "Transformed GVIF"), align="lrrr", ...) %>%
    kable_styling()
  return(table)
}
```

# 1 Introduction

This R Markdown file contains all the code used for data exclusion, analysis, and plotting. It describes each step of the analysis and includes all statistical results (e.g. model summaries). For further description of materials and methods, see the Detailed Materials and Methods Supplement.

# 2 Experiment 1: Word identification task

In Experiment 1, non-Spanish-speaking Americans assigned wordhood confidence ratings to words with varying frequencies and phonotactic probabilities, together with phonotactically-matched nonwords.

We provide an overview of participants’ demographics below; for further details of participants, including exclusion criteria, see Detailed Materials and Methods Section 1 (especially 1.1). For details of the stimuli, see Detailed Materials and Methods Section 2 (especially 2.3). For details of the procedure, see Detailed Materials and Methods Section 3 (especially 3.1).

```
# Load Exp1 data and filter participants

dataExp1 = read.csv("../data/word-identification_responses.csv", header = TRUE, encoding = "UTF-8")
dataExp1$txt = as.character(dataExp1$txt)

# Set up factor levels
dataExp1$freq = factor(dataExp1$freq, levels=c("low", "mid", "high"))
dataExp1$age = factor(dataExp1$age, levels=c("18-29", "30-39", "40-49", "50-59", ">60"))
dataExp1$education = factor(dataExp1$education, levels=c("highSchool", "2yrCollege", "4yrCollege", "grad"))

# Remove trials for items identified as problematic after the experiment
dataExp1 = dataExp1 %>%
  # Remove trials where a participant saw the same phonological form twice (with and without accent)
  # this is the pairs: garado/valido (gárado/válido) and mosque/porque (mosqué/porqué)
  anti_join(
    dataExp1 %>%
      group_by(workerId, phon) %>%
      tally() %>%
      ungroup() %>%
      filter(n > 1),
    by = c("workerId", "phon")
  ) %>%
  # Remove trials for items that are orthographically identical to English words, and their matched pairs
  anti_join(
    dataExp1 %>%
      filter(english) %>%
      dplyr::select(txt, pair) %>%
      distinct(),
    by="pair"
  ) %>%
  # Remove trials for items where a word-nonword pair are mismatched for length or score, according to revised phonological forms
  anti_join(
    dataExp1 %>%
      dplyr::select(txt, pair, type, length, score) %>%
      distinct() %>%
      pivot_wider(id_cols=pair, names_from=type, values_from=c(txt, length, score)) %>%
      filter(length_real != length_pseudo | abs(score_real - score_pseudo) >= 0.15),
    by="pair"
  ) %>%
  # Remove trials for items that have a score lower than -1.3, and their matched pairs
  anti_join(
    dataExp1 %>%
      filter(score <= -1.3) %>%
      dplyr::select(txt, score, pair) %>%
      distinct(),
    by="pair"
  )
  
# Load participants list, for ease of tracking participant-level exclusions
participantsExp1 = read.csv("../data/word-identification_workers.csv", header = TRUE)
participantsExp1$age = factor(participantsExp1$age, levels=c("18-29", "30-39", "40-49", "50-59", ">60"))
participantsExp1$education = factor(participantsExp1$education, levels=c("highSchool", "2yrCollege", "4yrCollege", "grad"))

participantsExp1 = participantsExp1 %>%
  filter(!(
    numNone > 3  # Remove participants who skipped more than 3 trials (4 removed)
    | str_detect(otherLang, fixed("Spanish"))  # Remove participants who list Spanish as a language they speak well (18 removed)
    | livedSpanish  # Remove participants who have lived in a Spanish-speaking country (0 removed)
    | str_detect(spanishEd, fixed("grad"))  # Remove participants who have studied Spanish at college level (12 removed)
    | speakSpanish >= 3 | understandSpanish >= 3  # Remove participants whose Spanish speaking / comprehension scores are 3 or higher (6 removed)
    | romanceLang  # Remove participants who speak another Romance language (1 removed)
    | livedRomance  # Remove participants who have lived in a country in which a Romance language is widely spoken (1 removed)
    | numMode > 180  # Remove participants who used a single rating for more than 180/240 trials (4 removed)
  ))

# Check the total number of usable participants for Exp1 (40 usable participants)
# nrow(participantsExp1)

# Copy participant-level exclusions to trials
dataExp1 = dataExp1 %>%
  semi_join(
    participantsExp1,
    by="workerId"
  )

# Filter individual trials
dataExp1 = dataExp1 %>%
  filter(!(
    is.na(response)  # Remove trials with no response (8 removed)
    | rt_ms < 250  # Remove trials with RT faster than 250ms (3 removed)
  ))

# Check the total number of usable trials for Exp1 (8115 usable trials)
# nrow(dataExp1)


# Get the by-item summary
itemsExp1 = dataExp1 %>%
  group_by(txt, phon, length, score, type, pair, freq, borrowing) %>%
  summarise(
    mean_response = mean(response),
    n = n()
  ) %>%
  ungroup()
```

## 2.1 Dataset structure

The dataset is structured as follows:

- *workerId* is the unique ID for each participant.
- *ip\_region* is the region from which the participant’s IP address was logged.
- *state* is the State that the participant indicated they grew up in and currently reside in.
- *time\_outside\_state* is the longest period of time the participant has spent outside of their State since the age of 7.
- *spanishEd* is the highest level of education at which the participant has studied Spanish.
- *speakSpanish* is the participant’s rating of how well they can speak Spanish (with a scale ranging from 0 to 5).
- *understandSpanish* is the participant’s rating of how well they can understand/read Spanish (with a scale ranging from 0 to 5).
- *spanishProf* is the sum of quantified response for speakSpanish and understandSpanish, which refers to the level of the participant’s Spanish proficiency (results can range from 0 to 10).
- *age* is the age group that the participant belongs to.
- *gender* is the gender of the participant (grouped into categories).
- *ethnicity* is the ethnicity of the partipant (grouped into categories based on free responses).
- *politics* is the political party with which the participant identifies most strongly.
- *education* is the participant’s highest level of education.
- *basicSpanish* is the basic elements of Spanish that the participant is able to use.
- *basicSpanishCount* is the number of basic elements of Spanish that the participant is able to use (results can range from 0 to 8).
- *spanishExpoMedia* is a log-like estimate of how often the participant is exposed to Spanish through the media (results can range from 1 to 5).
- *spanishExpoSocial* is a log-like estimate of how often the participant is exposed to Spanish through socializing (results can range from 1 to 5).
- *spanishExpo* is the sum of quantified response for spanishExpoMedia and spanishExpoSocial, which refers to the level of the participant’s overall exposure to Spanish (results can range from 2 to 10).
- *otherLang* is a list of the other languages that the participant can speak well.
- *romanceLang* indicates whether the participant can speak or understand any other Romance languages, even at a basic level.
- *romanceLang\_specify* is a list of the other Romance languages that the participant can speak or understand.
- *livedSpanish* indicates whether the particpant has lived in a country outside the US where Spanish is predominantly spoken.
- *livedSpanish\_specify* is a list of the Spanish-speaking countries in which the participant has lived.
- *livedRomance* indicates whether the particpant has lived in a country outside the US where a Romance language is predominantly spoken.
- *livedRomance\_specify* is a list of the Romance-language-speaking countries in which the participant has lived.
- *proSpanishLang* is the participant’s level of agreement with the statement: “Some Spanish language education should be compulsory in school for all children in ” (responses can range from 1 to 5).
- *proSpanishCult* is the participant’s level of agreement with the statement: “Hispanic and Latino cultures are important in ” (responses can range from 1 to 5).
- *proSpanish* is a measure of how strongly the participant values Spanish language and culture in their home state, obtained by adding the quantified responses to proSpanishLang and proSpanishCult and subtracting 6 (results can range from -4 to 4; positive numbers indicate a positive attitude toward Spanish). This variable is referred to as *Spanish value* in the paper.
- *proEnglishUSA* is the participant’s level of agreement with the statement: “People in the United States should speak English, not foreign languages” (responses can range from 1 to 5).
- *proImmigration* is the participant’s completion of the statement: “I think that the number of immigrants from foreign countries who are permitted to come to the United States to live should be \_\_\_\_\_” (responses can range from 1 to 5, with scores below 3 indicating decrease in immigration and scores above 3 indicating increase in immigration).
- *nationalism* is a measure of the strength of the participant’s nationalist feelings toward the US, obtained by subtracting the quantified response to proImmigration from the quantified response to proEnglishUSA (results can range from -4 to 4; positive numbers indicate a nationalist attitude toward the US).
- *txt* is the orthographic form of an item, presented as a stimulus.
- *phon* is the phonological form of the item.
- *length* is the phoneme length of the item.
- *score* is the phonotactic score for the item.
- *scoreSpOrth* is the score for the item according to Spanish orthotactics (ignoring diacritics), based on the same set of word types use for phonotactic scores.
- *scoreEngOrth* is the score for the item according to English orthotactics (ignoring diacritics), based on word types without non-alphabetic characters that occur in the CMU Pronouncing Dictionary.
- *type* is the ground-truth classification of the item: word (‘real’) or nonword (‘pseudo’).
- *borrowing* is whether the item is a Spanish word that has been borrowed into English, as determined by its appearance in the English lexicon captured by the CMU Pronouncing Dictionary. This includes a few words of Spanish origin which are often used in English without their Spanish diacritic: señor (*senor*), inglés (*ingles*), garcía (*garcia*), and político (*politico*). Such items are *not* removed from our analysis, but are tagged for further study.
- *english* is whether the item is orthographically identical to an English word (which does not appear Spanish in origin); for example, the same word of Latinate origin is used in both English and Spanish, or an English word has been borrowed into Spanish. Such items are removed from our analysis, but are retained in the dataset for further study.
- *diacriticEnglish* is whether the item would be orthographically identical to an English word as above, except that it contains a diacritic that is not typical in English words: <á, é, í, ó, ú, ñ>. Such items are *not* removed from our analysis, but are tagged for further study.
- *diacritic* is whether or not the orthographic item contains a diacritic that is not typical in English words: <á, é, í, ó, ú, ñ>
- *pair* is the ID used to pair phonotactically-similar word and nonword items.
- *freq* is the frequency bin of the real word in a word-nonword pair.
- *response* is the wordhood confidence rating for a stimulus.
- *rt\_ms* is the reaction time for the response (in milliseconds).

The formulations of *proSpanish* and *nationalism* are by design, but have also been confirmed by exploratory factor analysis of responses to the 4 attitude questions across all unique participants analyzed in either experiment (see Appendix C of this supplement for details). We choose to use the manually-designed formulations in our analysis, rather than the inferred factors, in order to ensure that the formulation is independent of the precise set of participants who took the experiment.

## 2.2 Overview of participants’ demographics

Figure S1a summarizes the distribution of participants on demographic and linguistic axes.

```
# Plot overview of participants in Exp1

# Recode ethnicity factor to fit on axis labels
participantsExp1 = participantsExp1 %>%
  mutate(
    ethnicity = fct_recode(ethnicity, 
                           "Asian"="Asian",
                           "Black"="Black",
                           "Hisp. /\nLatinx"="Hispanic",
                           "multi" = "multiple",
                           "Native\nAmerican" = "Native American",
                           "not\nspec." = "not specified",
                           "White"="White",
                           "White &\nAsian" = "White & Asian"
                           )
  )

plots_specs = list(
  basicSpanishCount = list(xlab="Number of elements known", title="Knowledge of basic Spanish elements"),
  spanishProf = list(xlab="Spanish proficiency rating", title="Degree of Spanish proficiency"),
  spanishExpo = list(xlab="Spanish exposure rating", title="Degree of exposure to Spanish"),
  gender = list(xlab="Gender", title="Gender"),
  age = list(xlab="Age group", title="Age"),
  education = list(xlab="Highest education", title="Education"),
  state = list(xlab="Home state", title="State"),
  ethnicity = list(xlab="Ethnicity", title="Ethnicity"),
  politics = list(xlab="Political party most identified with", title="Political affiliation"),
  proSpanish = list(xlab="Score of Spanish value in home state\nbased on ratings of language and culture", title="Spanish value"),
  nationalism = list(xlab="Score of nationalism\nbased on ratings of immigration and English", title="Nationalism")
)

# Make plots
plots_list = imap(plots_specs, 
                  function(fields, varname) {
                    return(make_count_plot(participantsExp1, varname, fields$xlab, "Number of participants", fields$title))
                  })

ggarrange(plots=plots_list, ncol=3)

remove(plots_specs, plots_list)
```

Figure S1a: Overview of participants’ demographics in Exp1.

Figure S1b shows correlations between the key individual difference predictors of *Spanish value*, *nationalism*, and *exposure* for participants in Exp1. We quantified these correlations using the `polychor()` function in the `polycor` package, as follows:

- There is a small negative polychoric correlation between *Spanish value* and *nationalism* (\(\rho=-0.135\)): participants with high Spanish value tend to have low nationalism, but there are also many participants with above-zero Spanish value and above-zero nationalism.
- There is a reasonable polychoric correlation between *Spanish value* and *Spanish exposure* (\(\rho=0.532\)): participants with high Spanish value tend to have more exposure to Spanish than others, while participants with very low Spanish value tend to have very little Spanish exposure, but there is still a lot of variation in degrees of exposure across Spanish value levels.
- Finally, there is a small negative polychoric correlation between *nationalism* and *Spanish exposure* (\(\rho=-0.244\)): participants with very high nationalism tend not to have much Spanish exposure, but there are also participants across the nationalism continuum with low Spanish exposure. The data visualization suggests a non-linear relationship between nationalism and Spanish exposure, where participants with extreme nationalism scores (either positive or negative) tend not to have as much Spanish exposure as participants with near-zero nationalism scores.

To ensure that these correlations do not create harmful multicollinearity, we report Variance Inflation Scores when we model the effects of attitudes and exposure.

```
corplot_a = participantsExp1 %>%
  dplyr::count(across(c(proSpanish, nationalism)), .drop=FALSE) %>%
  ggplot(., aes(x=proSpanish, y=nationalism)) +
  geom_hline(yintercept=0, linetype="dashed", color="darkgray", size=2) +
  geom_vline(xintercept=0, linetype="dashed", color="darkgray", size=2) +
  geom_smooth(data=participantsExp1, method="loess", formula="y~x", alpha=0.3) +
  geom_point(aes(size=n)) +
  scale_size_continuous(name="Participants", range=c(3,7), breaks=1:3) +
  xlab("Spanish value") +
  ylab("Nationalism") +
  coord_cartesian(xlim=c(-4, 4), ylim=c(-4, 4)) +
  theme_bw() +
  theme(
    panel.grid = element_blank(),
    axis.title = element_text(size=16, color="black"),
    axis.text = element_text(size=14, color="black"),
    legend.title = element_text(size=16, color="black"),
    legend.text = element_text(size=15, color="black")
  )

corplot_b = participantsExp1 %>%
  dplyr::count(across(c(proSpanish, spanishExpo)), .drop=FALSE) %>%
  ggplot(., aes(x=proSpanish, y=spanishExpo)) +
  geom_smooth(data=participantsExp1, method="loess", formula="y~x", alpha=0.3) +
  geom_point(aes(size=n)) +
  scale_size_continuous(name="Participants", range=c(3,9), breaks=1:7) +
  xlab("Spanish value") +
  ylab("Spanish exposure") +
  coord_cartesian(xlim=c(-4, 4), ylim=c(2, 10)) +
  theme_bw() +
  theme(
    panel.grid = element_blank(),
    axis.title = element_text(size=16, color="black"),
    axis.text = element_text(size=14, color="black"),
    legend.title = element_text(size=16, color="black"),
    legend.text = element_text(size=15, color="black")
  )

corplot_c = participantsExp1 %>%
  dplyr::count(across(c(nationalism, spanishExpo)), .drop=FALSE) %>%
  ggplot(., aes(x=nationalism, y=spanishExpo)) +
  geom_smooth(data=participantsExp1, method="loess", formula="y~x", alpha=0.3) +
  geom_point(aes(size=n)) +
  scale_size_continuous(name="Participants", range=c(3,7), breaks=1:5) +
  xlab("Nationalism") +
  ylab("Spanish exposure") +
  coord_cartesian(xlim=c(-4, 4), ylim=c(2, 10)) +
  theme_bw() +
  theme(
    panel.grid = element_blank(),
    axis.title = element_text(size=16, color="black"),
    axis.text = element_text(size=14, color="black"),
    legend.title = element_text(size=16, color="black"),
    legend.text = element_text(size=15, color="black")
  )

null_plot = ggplot() + theme_void()

ggarrange(plots=list(corplot_a, null_plot, corplot_b, null_plot, corplot_c), ncol=1, heights=c(1, 0.1, 1, 0.1, 1))

remove(corplot_a, corplot_b, corplot_c, null_plot)
```

Figure S1b: Correlations between participants’ Spanish value, nationalism, and Spanish exposure in Exp1.

## 2.3 Visualizations of raw data

### 2.3.1 Distributions of ratings across bins and stimulus types

Real words appear to receive higher ratings than nonwords. The effect is minimal in the low- and mid-frequency bins, but is noticeably larger in the high-frequency bin.

(Note: some of the highest-rated words are cognate to English words, such as *político* and *clínica*, or are Spanish words borrowed into English, such as *padre* and *grande*.)

```
# Plot raw data for Exp1, by stimulus type and frequency.

ggplot(itemsExp1, aes(x=freq, y=mean_response, fill=type)) +
  geom_violin(alpha=0.4, position=position_identity()) +
  stat_summary(geom="point", fun=mean, aes(shape=type), size=5) +
  stat_summary(geom="line", fun=mean, aes(color=type, linetype=type, group=type), size=1) +
  scale_fill_manual(name="Stimulus type",
                    labels = c("pseudo" = "Nonword", "real" = "Word"),
                    values = c("pseudo" = "black", "real" = "blue"),
                    guide = guide_legend(reverse = TRUE)) +  
  scale_shape_manual(name="Stimulus type",
                     labels = c("pseudo" = "Nonword", "real" = "Word"),
                     values = c("pseudo" = 21, "real" = 24),
                     guide = guide_legend(reverse = TRUE)) +   
  scale_color_manual(name="Effect estimate",
                     labels = c("pseudo" = "Nonword", "real" = "Word"),
                     values = c("pseudo" = "black", "real" = "blue"),
                     guide = "none") +
  scale_linetype_manual(name="Effect estimate",
                        labels = c("pseudo" = "Nonword", "real" = "Word"),
                        values = c("pseudo" = "solid", "real" = "dotted"),
                        guide = "none") + 
  labs(y = "Mean Rating (per stimulus)", x = "Frequency Bin") +
  ylim(1, 5) +
  theme_bw() + 
  theme(
    panel.grid = element_blank(),
    axis.title = element_text(size=16, color="black"),
    axis.text = element_text(size=14, color="black"),
    legend.title = element_text(size=16, color="black"),
    legend.text = element_text(size=15, color="black")
  )
```

Figure S2: Mean wordhood confidence ratings for each stimulus per frequency bin. Each nonword is assigned to frequency bin of its phonotactically matched real word. Points represent mean ratings across all real words and nonwords within each bin (joined by lines as a visual aid only).

### 2.3.2 Mean rating and phonotactic score for each stimulus per frequency bin

There is some indication that both words and nonwords with higher phonotactic scores are rated more likely to be real Spanish words, except in the low-frequency bin. The pattern is strongest in the mid-frequency bin, and among nonwords in the high-frequency bin; it is weaker for real words in the high-frequency bin, where it is possible that participants are more influenced by explicit knowledge.

```
# Plot raw data for Exp1, by stimulus type, frequency, and phonotactic score.

ggplot(itemsExp1, aes(x=score, y=mean_response, color=type)) +
  geom_point(aes(shape=type), alpha=0.3, size=4) +
  geom_smooth(aes(fill=type), method="lm", formula="y~x", alpha=0.6, size=2) +
  facet_grid(. ~ freq, labeller=as_labeller(function(value) str_to_sentence(paste0(value, "-frequency")))) +
  xlab("Phonotactic score") +
  ylab("Wordhood confidence\nMean rating (per stimulus)") +
  scale_shape_manual(name="Stimulus type",
                     labels = c("pseudo" = "Nonword", "real" = "Word"),
                     values = c("pseudo" = 1, "real" = 2),
                     guide = guide_legend(reverse = TRUE)) +   
  scale_color_manual(name="Stimulus type",
                     labels = c("pseudo" = "Nonword", "real" = "Word"),
                     values = c("pseudo" = "black", "real" = "blue"),
                     guide = guide_legend(reverse = TRUE)) +
  scale_fill_manual(name="Stimulus type",
                    labels = c("pseudo" = "Nonword", "real" = "Word"),
                    values = c("pseudo" = "gray70", "real" = "dodgerblue1"),
                    guide = guide_legend(reverse = TRUE)) +  
  theme_bw() + 
  ylim(1, 5) +
  theme(
    panel.grid = element_blank(),
    axis.title = element_text(size=16, color="black"),
    axis.text = element_text(size=14, color="black"),
    legend.title = element_text(size=16, color="black"),
    legend.text = element_text(size=15, color="black"),
    strip.text = element_text(size=15, color="black")
  )
```

Figure S3: Mean rating vs. phonotactic score for each stimulus for real words and nonwords by frequency bin. Lines show correlations within each bin, for each stimulus type.

## 2.4 Statistical analysis

We use mixed-effects ordinal regression to predict participants’ wordhood confidence ratings. For an introduction to ordinal regression, see Section 5.1 of the Detailed Materials and Methods of Oh et al. (2020).

The predictors considered in the analysis are:

- `length`: Stimulus length in phonemes (centered)
  - Note: we include length because it was found to have an effect by Oh et al. (2020); however, owing to the additional measures we took to balance stimuli across lengths (see Section 2.1 of the Detailed Materials and Methods), we do not expect it to have an effect here.
- `score`: Stimulus phonotactic score (centered)
- `type`: Stimulus type: word or nonword (binary factor)
- `diacritic`: Presence of a diacritic not typically found in English words (binary factor)
  - Note: we include this predictor both because it captures a visual component that is distinct from phonotactics, and because accented vowel characters indicate stress, which is not captured by our phonotactic model.
- `freq`: Frequency bin (Helmert-coded factor)
  - Note: we use manually-defined Helmert contrasts rather than the built-in `contr.helmert` function, to ensure interpretable scaling.

We consider `score` and `type` to be the predictors of primary interest, while `diacritic`, `length` and `freq` are control predictors.

We follow the procedure of Oh et al. (2020), in an effort to closely replicate their work. This means that we perform a step-down model fitting procedure in a fixed-effects setting (`clm`) before transitioning to a mixed-effects setting (`clmm`). We begin by considering a model containing all two-way interactions between a predictor of primary interest (`score` and `type`) and a control predictor (`diacritic`, `length` and `freq`), as well as the interaction between the two predictors of primary interest. We remove interactions one at a time if they do not make a significant contribution to the model’s explanatory power, assessed via Analysis of Deviance (using a type III test from the `anova` function), starting with the interaction with the least contribution. We then remove main effects one a time if they are not included in any remaining interactions and they do not make a significant contribution to the model’s explanatory power. Once there are no more candidate terms for removal, we re-fit the model in a mixed-effects setting, adding random intercepts by participant and item and random slopes by participant for each remaining term. For practical reasons of computing time, we identify candidates for removal in the mixed-effects setting if their coefficient is not significant in the model; after performing each removal, we confirm that it was justified via model comparison, using a log-likelihood ratio test.

### 2.4.1 Results

Table S1 reports the results of the ordinal regression analysis.

We see a significant main effect of stimulus `type`, indicating that real words are rated higher than nonwords. Additionally, we see a significant interaction between stimulus `type` and `freq`uency, indicating that the highest-frequency real words are rated higher relative to their matched nonwords than would be expected based on all other words and nonwords.

We also see a significant main effect of phonotactic `score`, where stimuli (words and nonwords) with higher phonotactic scores receive higher ratings.

Finally, we see a significant main effect of the presence of a `diacritic`, indicating that stimuli (words and nonwords) that contain at least one non-English character are rated higher than those that don’t contain any English characters.

For ease of interpretation, we present partial effect plots of the ordinal regression model in Figure S4.

```
# Analysis of Exp1 main results

dataExp1$response <- as.factor(dataExp1$response)

helmert <- matrix(c(c(-1,1,0)/2, c(-1,-1,2)/3), ncol=2)
colnames(helmert) <- c("mid.v.low", "high.v.others")
contrasts(dataExp1$freq) <- helmert
remove(helmert)

# Model fitting process: fixed-effects only
# m1 <- clm(response ~ (c.(score) + type) * (diacritic + c.(length) + freq) + c.(score):type, data=dataExp1)
# anova(m1, type="III") # Removal candidate: type:c.(length)
# m2 <- update(m1, . ~ . - type:c.(length))
# anova(m2, type="III") # Removal candidate: c.(score):c.(length)
# m3 <- update(m2, . ~ . - c.(score):c.(length))
# anova(m3, type="III") # Removal candidate: c.(score):diacritic
# m4 <- update(m3, . ~ . - c.(score):diacritic)
# anova(m4, type="III") # Removal candidate: type:diacritic
# m5 <- update(m4, . ~ . - type:diacritic)
# anova(m5, type="III") # Removal candidate: c.(score):freq
# m6 <- update(m5, . ~ . - c.(score):freq)
# anova(m6, type="III") # Removal candidate: c.(score):type
# m7 <- update(m6, . ~ . - c.(score):type)
# anova(m7, type="III") # Removal candidate: c.(length)
# m8 <- update(m7, . ~ . - c.(length))
# anova(m8, type="III") # No further candidate predictors for removal.

# Switch to mixed-effects

# m9 <- clmm(response ~ c.(score) + diacritic + type * freq + (1 + c.(score) + diacritic + type * freq|workerId) + (1|phon), data=dataExp1)
# summary(m9) # No candidate predictors for removal
# 
# saveRDS(m9, file = "dumps/clmm/modelExp1.rds")
# remove(m1, m2, m3, m4, m5, m6, m7, m8, m9)
modelExp1 <- readRDS("dumps/clmm/modelExp1.rds")

clm_table(modelExp1, caption="Table S1: Ordinal mixed-effects model of wordhood confidence ratings. Phonotactic score is centered, and frequency bin is Helmert-coded.")
```

Table S1: Ordinal mixed-effects model of wordhood confidence ratings. Phonotactic score is centered, and frequency bin is Helmert-coded.

|  | Parameter | Estimate | Std. Error | \(z\) | \(p\) |  |
| --- | --- | --- | --- | --- | --- | --- |
| Effects | score (centered) | 1.083 | 0.355 | 3.050 | 0.002 | \*\* |
|  | diacritic = TRUE | 1.122 | 0.263 | 4.264 | <0.001 | \*\*\* |
|  | type = real | 0.459 | 0.104 | 4.426 | <0.001 | \*\*\* |
|  | freq = mid.v.low | 0.082 | 0.108 | 0.764 | 0.445 |  |
|  | freq = high.v.others | -0.078 | 0.100 | -0.778 | 0.437 |  |
|  | type = real × freq = mid.v.low | 0.045 | 0.152 | 0.297 | 0.766 |  |
|  | type = real × freq = high.v.others | 0.542 | 0.155 | 3.489 | <0.001 | \*\*\* |
| Thresholds | 1|2 | -2.638 | 0.144 |  |  |  |
|  | 2|3 | -0.922 | 0.140 |  |  |  |
|  | 3|4 | 0.490 | 0.139 |  |  |  |
|  | 4|5 | 2.713 | 0.143 |  |  |  |

```
# Plot Exp1 main results

figS4a.mean <- clm_plotdat(modelExp1, c("type", "freq"), type="mean") %>%
  mutate(
    type = fct_recode(type, "Nonword"="pseudo", "Word"="real") %>% fct_relevel("Word")
  ) %>%
  ggplot(., aes(x=as.integer(freq), y=pred, color=type, fill=type, shape=type)) +
    geom_ribbon(aes(ymin=lci, ymax=uci), alpha=0.1, color=NA) +
    geom_line(size=1, alpha=0.4) +
    geom_point(size=4) +
    geom_errorbar(aes(ymin=lci, ymax=uci), size=1, width=0.3) +
    scale_x_continuous(name="Frequency Bin", breaks=1:3, labels=c("Low", "Mid", "High")) +
    ylab("Predicted mean rating") +
    ylim(2.9, 4.1) +
    scale_shape_manual(values = c("Nonword" = 19, "Word" = 17)) +
    scale_color_manual(values = c("Nonword" = "black", "Word" = "blue")) +
    scale_fill_manual(values = c("Nonword" = "black", "Word" = "blue")) +
    theme_bw() +
    theme(
      legend.position="none",
      legend.title=element_blank()
    )

figS4a.dist <- clm_plotdat(modelExp1, c("type", "freq"), type="dist") %>%
  mutate(
    type = fct_recode(type, "Nonword"="pseudo", "Word"="real") %>% fct_relevel("Word")
  ) %>%
  ggplot(aes(x=as.integer(freq), y=pred, color=type, fill=type, shape=type)) +
    geom_ribbon(aes(ymin=lci, ymax=uci), alpha=0.2, color=NA) +
    geom_line(size=1, alpha=0.4) +
    geom_point(size=2) +
    geom_errorbar(aes(ymin=lci, ymax=uci), size=1, width=0.3) +
    facet_grid(. ~ response, labeller=as_labeller(function(x) str_c("Rating: ", x))) +
    ylim(0, 1) +
    scale_x_continuous(name="Frequency Bin", breaks=1:3, labels=c("Low", "Mid", "High")) +
    ylab("Probability of rating") +
    scale_shape_manual(values = c("Nonword" = 19, "Word" = 17)) +
    scale_color_manual(values = c("Nonword" = "black", "Word" = "blue")) +
    scale_fill_manual(values = c("Nonword" = "black", "Word" = "blue")) +
    theme_bw() +
    theme(
      legend.position="right",
      legend.title=element_blank()
    )

figS4b.mean <- clm_plotdat(modelExp1, "score", xlevels=25, type="mean") %>%
  mutate(type="Word and\nNonword") %>%
  ggplot(., aes(x=score, y=pred, color=type, fill=type)) +
    geom_ribbon(aes(ymin=lci, ymax=uci), alpha=0.3, color=NA) +
    geom_line(size=1) +
    scale_x_continuous(n.breaks=4) +
    xlab("Phonotactic score") +
    ylab("Predicted mean rating") +
    ylim(2.9, 4.1) +
    scale_color_manual(values = c("Word and\nNonword" = "purple")) +
    scale_fill_manual(values = c("Word and\nNonword" = "purple")) +
    theme_bw() +
    theme(
      legend.position="none",
      legend.title=element_blank()
    )

figS4b.dist <- clm_plotdat(modelExp1, "score", xlevels=25, type="dist") %>%
  mutate(type="Word and\nNonword") %>%
  ggplot(., aes(x=score, y=pred, color=type, fill=type)) +
    geom_ribbon(aes(ymin=lci, ymax=uci), alpha=0.3, color=NA) +
    geom_line(size=1) +
    scale_x_continuous(n.breaks=4) +
    xlab("Phonotactic score") +
    ylab("Probability of rating") +
    scale_color_manual(values = c("Word and\nNonword" = "purple")) +
    scale_fill_manual(values = c("Word and\nNonword" = "purple")) +
    facet_grid(. ~ response, labeller=as_labeller(function(x) str_c("Rating: ", x))) +
    ylim(0, 1) +
    theme_bw() +
    theme(
      legend.position="right",
      legend.title=element_blank()
    )

figS4c.mean <- clm_plotdat(modelExp1, "diacritic", type="mean") %>%
  mutate(
    type = "Word and\nNonword",
    diacritic = factor(diacritic, levels=c(TRUE, FALSE), labels=c("Present", "Absent"))
  ) %>%
  ggplot(., aes(x=diacritic, y=pred, color=type, fill=type)) +
    geom_point(size=4) +
    geom_errorbar(aes(ymin=lci, ymax=uci), size=1, width=0.3) +
    xlab("Non-English characters") +
    ylab("Predicted mean rating") +
    ylim(2.9, 4.1) +
    scale_color_manual(values = c("Word and\nNonword" = "purple")) +
    scale_fill_manual(values = c("Word and\nNonword" = "purple")) +
    theme_bw() +
    theme(
      legend.position="none",
      legend.title=element_blank()
    )

figS4c.dist <- clm_plotdat(modelExp1, "diacritic", type="dist") %>%
  mutate(
    type = "Word and\nNonword",
    diacritic = factor(diacritic, levels=c(TRUE, FALSE), labels=c("Present", "Absent"))
  ) %>%
  ggplot(., aes(x=diacritic, y=pred, color=type, fill=type)) +
    geom_point(size=4) +
    geom_errorbar(aes(ymin=lci, ymax=uci), size=1, width=0.3) +
    xlab("Non-English characters") +
    ylab("Probability of rating") +
    scale_color_manual(values = c("Word and\nNonword" = "purple")) +
    scale_fill_manual(values = c("Word and\nNonword" = "purple")) +
    facet_grid(. ~ response, labeller=as_labeller(function(x) str_c("Rating: ", x))) +
    ylim(0, 1) +
    theme_bw() +
    theme(
      legend.position="right",
      legend.title=element_blank()
    )

ggarrange(figS4a.mean, figS4a.dist, figS4b.mean, figS4b.dist, figS4c.mean, figS4c.dist, ncol=2, labels=c("a.", "", "b.", "", "c.", ""), widths=c(1,5))

remove(figS4a.mean, figS4a.dist, figS4b.mean, figS4b.dist, figS4c.mean, figS4c.dist)
```

Figure S4: Partial effect plots for ordinal mixed-effects model of wordhood confidence ratings. Left-hand plots show predicted mean ratings; right-hand plots show predicted distributions over ratings (facets). Figure S4a (top) shows the interaction between frequency bin and lexicality (real words vs. nonwords); Figure S4b (middle) shows the effect of phonotactic score; Figure S4c (bottom) shows the effect of presence or absence of non-English characters. Error bars and ribbons represent 95% confidence intervals.

Given the presence of the interaction between `type` and `freq`, the main effect of `type` in the model tells us that real words are rated higher relative to their matched nonwords *when averaging across all frequency bins*. The effects of `freq` tell us that this pattern is stronger in the high-frequency bin than in other bins; however, it is possible that it is *only* evident in the high-frequency bin. To determine whether real words are rated higher than nonwords *within each frequency bin* (i.e. that even low- and mid-frequency words are rated higher than their matched nonwords), we run a post-hoc comparison of estimated marginal means with the `emmeans` package, which compares the model’s predicted ratings for real words (the treatment case) against ratings for pseudowords (the control case) within each frequency bin. The results, shown in Table S2, indicate that, on average, real words are rated significantly higher than nonwords *in each frequency bin*.

```
emm = emmeans(modelExp1, specs= trt.vs.ctrl ~ type|freq)

emm$contrasts %>%
  summary(infer = TRUE) %>%
  as.data.frame() %>%
  mutate(
    confint = str_c("[", round(asymp.LCL, 3), ", ", round(asymp.UCL, 3), "]"),
    significance = case_when(
      p.value < 0.001 ~ "\\*\\*\\*",
      p.value < 0.01 ~ "\\*\\*",
      p.value < 0.05 ~ "\\*",
      p.value < 0.1 ~ ".",
      TRUE ~ ""
    ),
    p.value = ifelse(p.value<0.001, "<0.001", format(round(p.value, 3), nsmall=3))
  ) %>%
  dplyr::select(freq, estimate, SE, confint, z=z.ratio, p=p.value, significance) %>%
  kable(digits=3, escape=F, col.names=c("Freq. bin", "EMM (word-nonword)", "Std. Error", "95% CI", "$z$", "$p$", ""), align="lrrrrrl", caption="Table S2: Post-hoc analysis of the difference between ratings for words and nonwords within each frequency bin, based on estimated marginal means.") %>%
  kable_styling()
```

Table S2: Post-hoc analysis of the difference between ratings for words and nonwords within each frequency bin, based on estimated marginal means.

| Freq. bin | EMM (word-nonword) | Std. Error | 95% CI | \(z\) | \(p\) |  |
| --- | --- | --- | --- | --- | --- | --- |
| low | 0.256 | 0.107 | [0.047, 0.465] | 2.403 | 0.016 | \* |
| mid | 0.301 | 0.125 | [0.056, 0.547] | 2.410 | 0.016 | \* |
| high | 0.821 | 0.182 | [0.465, 1.177] | 4.517 | <0.001 | \*\*\* |

## 2.5 Effects of attitudes and exposure

Having determined the basic structure of the results, we now explore the role that attitudes and exposure to Spanish play in these results.

### 2.5.1 Visualization of raw data

In general, there is some indication that participants who value Spanish and its speakers in their state, have low nationalism, and have high degrees of Spanish exposure may be better able to identify real high-frequency words, and that they may be more sensitive to phonotactic probability in giving their ratings. Among the three measures, Spanish value appears to be the most consistent (for example, the slopes representing sensitivity to phonotactic probability change in a more consistent manner across degrees of Spanish value than they do across degrees of nationalism or exposure, particularly for real worlds).

```
# Plot of raw data for Exp1, by stimulus type, frequency, phonotactic score, and attitude/exposure factors

valueplot = dataExp1 %>%
  mutate(
    value = factor(
      case_when(
        proSpanish < 0 ~ "negative",
        proSpanish > 0 ~ "positive",
        proSpanish == 0 ~ "neutral"
      ),
      levels = c("positive", "neutral", "negative")
    )
  ) %>%
  group_by(value, phon, score, type, freq) %>%
  summarise(
    mean_response = mean(as.numeric(response)),
    n = n()
  ) %>%
  ungroup() %>%
  ggplot(., aes(x=score, y=mean_response, color=type)) +
    geom_point(aes(shape=type), alpha=0.3, size=4) +
    geom_smooth(aes(fill=type), method="lm", formula="y~x", alpha=0.6, size=2) +
    facet_grid(value ~ freq, labeller="label_both", switch="y") +
    xlab("Phonotactic score") +
    ylab("Mean rating (per stimulus)") +
    scale_shape_manual(name="Stimulus type     ",
                       labels = c("pseudo" = "Nonword", "real" = "Word     "),
                       values = c("pseudo" = 1, "real" = 2),
                       guide = guide_legend(reverse = TRUE)) +   
    scale_color_manual(name="Stimulus type     ",
                       labels = c("pseudo" = "Nonword", "real" = "Word     "),
                       values = c("pseudo" = "black", "real" = "blue"),
                       guide = guide_legend(reverse = TRUE)) +
    scale_fill_manual(name="Stimulus type     ",
                      labels = c("pseudo" = "Nonword", "real" = "Word     "),
                      values = c("pseudo" = "gray70", "real" = "dodgerblue1"),
                      guide = guide_legend(reverse = TRUE)) +  
    theme_bw() + 
    ggtitle("Spanish value") +
    ylim(1, 5) +
    theme(
      panel.grid = element_blank(),
      axis.title = element_text(size=12, color="black"),
      axis.text = element_text(size=10, color="black"),
      legend.title = element_text(size=12, color="black"),
      legend.text = element_text(size=11, color="black"),
      strip.text = element_text(size=11, color="black"),
      plot.title = element_text(size=14, color="black", hjust=0.5),
      legend.position = "bottom"
    )

nationalismplot = dataExp1 %>%
  mutate(
    nationalism = factor(
      case_when(
        nationalism < 0 ~ "low",
        nationalism > 0 ~ "mid",
        nationalism == 0 ~ "high"
      ),
      levels = c("high", "mid", "low")
    )
  ) %>%
  group_by(nationalism, phon, score, type, freq) %>%
  summarise(
    mean_response = mean(as.numeric(response)),
    n = n()
  ) %>%
  ungroup() %>%
  ggplot(., aes(x=score, y=mean_response, color=type)) +
    geom_point(aes(shape=type), alpha=0.3, size=4) +
    geom_smooth(aes(fill=type), method="lm", formula="y~x", alpha=0.6, size=2) +
    facet_grid(nationalism ~ freq, labeller="label_both", switch="y") +
    xlab("Phonotactic score") +
    ylab("Mean rating (per stimulus)") +
    scale_shape_manual(name="Stimulus type     ",
                       labels = c("pseudo" = "Nonword", "real" = "Word     "),
                       values = c("pseudo" = 1, "real" = 2),
                       guide = guide_legend(reverse = TRUE)) +   
    scale_color_manual(name="Stimulus type     ",
                       labels = c("pseudo" = "Nonword", "real" = "Word     "),
                       values = c("pseudo" = "black", "real" = "blue"),
                       guide = guide_legend(reverse = TRUE)) +
    scale_fill_manual(name="Stimulus type     ",
                      labels = c("pseudo" = "Nonword", "real" = "Word     "),
                      values = c("pseudo" = "gray70", "real" = "dodgerblue1"),
                      guide = guide_legend(reverse = TRUE)) +  
    theme_bw() + 
    ggtitle("Nationalism") +
    ylim(1, 5) +
    theme(
      panel.grid = element_blank(),
      axis.title = element_text(size=12, color="black"),
      axis.text = element_text(size=10, color="black"),
      legend.title = element_text(size=12, color="black"),
      legend.text = element_text(size=11, color="black"),
      strip.text = element_text(size=11, color="black"),
      plot.title = element_text(size=14, color="black", hjust=0.5),
      legend.position = "bottom"
    )

expoplot = dataExp1 %>%
  mutate(
    exposure = factor(
      case_when(
        spanishExpo == 2 ~ "low",
        spanishExpo <= 4 ~ "mid",
        spanishExpo > 4 ~ "high"
      ),
      levels = c("high", "mid", "low")
    )
  ) %>%
  group_by(exposure, phon, score, type, freq) %>%
  summarise(
    mean_response = mean(as.numeric(response)),
    n = n()
  ) %>%
  ungroup() %>%
  ggplot(., aes(x=score, y=mean_response, color=type)) +
    geom_point(aes(shape=type), alpha=0.3, size=4) +
    geom_smooth(aes(fill=type), method="lm", formula="y~x", alpha=0.6, size=2) +
    facet_grid(exposure ~ freq, labeller="label_both", switch="y") +
    xlab("Phonotactic score") +
    ylab("Mean rating (per stimulus)") +
    scale_shape_manual(name="Stimulus type     ",
                       labels = c("pseudo" = "Nonword", "real" = "Word     "),
                       values = c("pseudo" = 1, "real" = 2),
                       guide = guide_legend(reverse = TRUE)) +   
    scale_color_manual(name="Stimulus type     ",
                       labels = c("pseudo" = "Nonword", "real" = "Word     "),
                       values = c("pseudo" = "black", "real" = "blue"),
                       guide = guide_legend(reverse = TRUE)) +
    scale_fill_manual(name="Stimulus type     ",
                      labels = c("pseudo" = "Nonword", "real" = "Word     "),
                      values = c("pseudo" = "gray70", "real" = "dodgerblue1"),
                      guide = guide_legend(reverse = TRUE)) +  
    theme_bw() + 
    ggtitle("Spanish exposure") +
    ylim(1, 5) +
    theme(
      panel.grid = element_blank(),
      axis.title = element_text(size=12, color="black"),
      axis.text = element_text(size=10, color="black"),
      legend.title = element_text(size=12, color="black"),
      legend.text = element_text(size=11, color="black"),
      strip.text = element_text(size=11, color="black"),
      plot.title = element_text(size=14, color="black", hjust=0.5),
      legend.position = "bottom"
    )

ggarrange(valueplot, nationalismplot, expoplot, ncol=3, labels=c("a.", "b.", "c."))
remove(valueplot, nationalismplot, expoplot)


# Figure from the paper:
# typeplot = dataExp1 %>%
#   mutate(
#     value = factor(
#       case_when(
#         proSpanish <= 0 ~ "neutr./neg.",
#         proSpanish > 0 ~ "pos."
#       ),
#       levels = c("pos.", "neutr./neg."), 
#       labels = c("Positive\nSpanish value", "Neutral / negative\nSpanish value")
#     ),
#     freq = factor(freq, levels=c("low", "mid", "high"), labels=c("Low-frequency", "Mid-frequency", "High-frequency"))
#   ) %>%
#   group_by(value, phon, score, type, freq) %>%
#   summarise(
#     mean_response = mean(as.numeric(response)),
#     n = n()
#   ) %>%
#   ungroup() %>%
#   ggplot(., aes(x=score, y=mean_response, color=type)) +
#     geom_point(aes(shape=type), alpha=0.3, size=4) +
#     geom_smooth(aes(fill=type), method="lm", formula="y~x", alpha=0.6, size=2) +
#     facet_grid(value ~ freq, switch="y") +
#     xlab("Phonotactic score") +
#     ylab("Mean rating (per stimulus)") +
#     scale_shape_manual(name="Stimulus type     ",
#                        labels = c("pseudo" = "Nonword", "real" = "Word     "),
#                        values = c("pseudo" = 1, "real" = 2),
#                        guide = guide_legend(reverse = TRUE)) +   
#     scale_color_manual(name="Stimulus type     ",
#                        labels = c("pseudo" = "Nonword", "real" = "Word     "),
#                        values = c("pseudo" = "black", "real" = "blue"),
#                        guide = guide_legend(reverse = TRUE)) +
#     scale_fill_manual(name="Stimulus type     ",
#                       labels = c("pseudo" = "Nonword", "real" = "Word     "),
#                       values = c("pseudo" = "gray70", "real" = "dodgerblue1"),
#                       guide = guide_legend(reverse = TRUE)) +  
#     theme_bw() + 
#     ylim(1, 5) +
#     theme(
#       panel.grid = element_blank(),
#       axis.title = element_text(size=16, color="black"),
#       axis.text = element_text(size=14, color="black"),
#       legend.title = element_text(size=16, color="black"),
#       legend.text = element_text(size=15, color="black"),
#       strip.text.x = element_text(size=15, color="black"),
#       strip.text.y = element_text(size=15, color="white", face="bold")
#     )
# 
# g <- ggplot_gtable(ggplot_build(typeplot))
# strip_left <- which(grepl('strip-l', g$layout$name))
# fills <- c("forestgreen", "firebrick3")
# k <- 1
# for (i in strip_left) {
# j <- which(grepl('rect', g$grobs[[i]]$grobs[[1]]$childrenOrder))
# g$grobs[[i]]$grobs[[1]]$children[[j]]$gp$fill <- fills[k]
# k <- k+1
# }
# grid.draw(g)
```

Figure S5: Mean rating vs. phonotactic score for each stimulus for real words and nonwords by frequency bin, partitioned according to participants’ self-reported Spanish value in their home state (a), nationalism (b), and exposure to Spanish (c). Lines show correlations within each bin and partition, for each stimulus type.

### 2.5.2 Statistical analysis

To statistically analyze the effects of attitudes and exposure in Experiment 1, we start from the final (reduced) model obtained in the previous stage of the analysis. We add terms involving value of Spanish and its speakers (`proSpanish`), nationalism (`nationalism`), and exposure to Spanish (`spanishExpo`), follow the model fitting procedure again for these terms, and then examine the resulting estimates. We allow each of the 3 predictors `proSpanish`, `nationalism`, and `spanishExpo` to interact with all of the already-established terms, but not with each other, for reasons of limited participant numbers.

We follow the same model fitting procedure as previously. First, we assess the maximal model in a fixed-effects setting, and use Analysis of Deviance to remove terms that do not contribute significantly to the model’s explanatory power. Then, we move into a mixed-effects setting, where our new predictors are given random by-item slopes, and continue to remove terms based on the significance of their effect and their contribution to the model’s explanatory value. We choose not to remove any main effects in the mixed-effects setting, so that the same random effect structure can be used throughout the analysis.

Table S3 reports the results of the analysis.

The interactions retained by the model-fitting procedure concern the degree to which participants value Spanish and its speakers in their home state. We see significant effects whereby participants who value Spanish more (i.e. with more positive attitudes) are significantly more able to identify real words of all frequencies than participants with who value Spanish less (i.e. with more negative attitudes) attitudes, and experience a significantly greater boost for identifying high-frequency words in particular. The same numerical pattern extends to a marginal boost for identifying mid-frequency words (relative to low-frequency words). Moreover, we see a significant effect whereby participants who value Spanish more are more sensitive to phonotactic scores in their assignment of ratings to items. In general, then, we see that participants who value Spanish and its speakers more (i.e. with more positive attitudes) show more evidence of implicit knowledge of Spanish in this experiment.

For ease of interpretation, we present partial effect plots in Figure S6.

```
# Statistical analysis of Exp1 results, taking into account attitude/exposure predictors

dataExp1$response <- as.factor(dataExp1$response)

# helmert <- matrix(c(c(-1,1,0)/2, c(-1,-1,2)/3), ncol=2)
# colnames(helmert) <- c("mid.v.low", "high.v.others")
# contrasts(dataExp1$freq) <- helmert
# remove(helmert)
# 
# m1 <- clm(response ~ (c.(score) + type * freq + diacritic) * (c.(proSpanish) + c.(nationalism) + c.(spanishExpo)), data=dataExp1)
# anova(m1, type="III") # Removal candidate: c.(score):c.(nationalism)
# m2 <- update(m1, . ~ . - c.(score):c.(nationalism))
# anova(m2, type="III") # Removal candidate: c.(score):c.(spanishExpo)
# m3 <- update(m2, . ~ . - c.(score):c.(spanishExpo))
# anova(m3, type="III") # Removal candidate: type:freq:c.(spanishExpo)
# m4 <- update(m3, . ~ . - type:freq:c.(spanishExpo))
# anova(m4, type="III") # Removal candidate: freq:c.(spanishExpo)
# m5 <- update(m4, . ~ . - freq:c.(spanishExpo))
# anova(m5, type="III") # Removal candidate: type:c.(spanishExpo)
# m6 <- update(m5, . ~ . - type:c.(spanishExpo))
# anova(m6, type="III") # Removal candidate: type:freq:c.(nationalism)
# m7 <- update(m6, . ~ . - type:freq:c.(nationalism))
# anova(m7, type="III") # Removal candidate: freq:c.(nationalism)
# m8 <- update(m7, . ~ . - freq:c.(nationalism))
# anova(m8, type="III") # Removal candidate: type:c.(nationalism)
# m9 <- update(m8, . ~ . - type:c.(nationalism))
# anova(m9, type="III") # No further removal candidates

# Switch to mixed-effects

# m10 <- clmm(response ~ (c.(score) + type * freq + diacritic) * c.(proSpanish) + diacritic * (c.(nationalism) + c.(spanishExpo)) + (1 + c.(score) + diacritic + type * freq|workerId) + (1 + c.(proSpanish) + c.(nationalism) + c.(spanishExpo)|phon), data=dataExp1)
# summary(m10) # Removal candidate: diacritic:c.(proSpanish)
# 
# m11 <- clmm(response ~ (c.(score) + type * freq) * c.(proSpanish) + diacritic * (c.(nationalism) + c.(spanishExpo)) + (1 + c.(score) + diacritic + type * freq|workerId) + (1 + c.(proSpanish) + c.(nationalism) + c.(spanishExpo)|phon), data=dataExp1)
# anova(m10, m11) # Removal justified
# summary(m11) # Removal candidate: diacritic:c.(nationalism)
# 
# m12 <- clmm(response ~ (c.(score) + type * freq) * c.(proSpanish) + diacritic * c.(spanishExpo) + c.(nationalism) + (1 + c.(score) + diacritic + type * freq|workerId) + (1 + c.(proSpanish) + c.(nationalism) + c.(spanishExpo)|phon), data=dataExp1)
# anova(m11, m12) # Removal justified
# summary(m12) # Removal candidate: diacritic:c.(spanishExpo)
# 
# m13 <- clmm(response ~ (c.(score) + type * freq) * c.(proSpanish) + diacritic + c.(nationalism) + c.(spanishExpo) + (1 + c.(score) + diacritic + type * freq|workerId) + (1 + c.(proSpanish) + c.(nationalism) + c.(spanishExpo)|phon), data=dataExp1)
# anova(m12, m13) # Removal justified
# summary(m13) # The only further removal candidates are main effects. Therefore, we stop here.
# 
# saveRDS(m13, file = "dumps/clmm/modelExp1_Attitudes.rds")
# remove(m1, m2, m3, m4, m5, m6, m7, m8, m9, m10, m11, m12, m13)

# Download file from Google drive if it doesn't already exist (it is too large for GitHub)
if (!file.exists("dumps/clmm/modelExp1_Attitudes.rds")) {
  download.file("https://drive.google.com/uc?export=download&id=1PsdE_8Z_hO-GFutiZDFvo9jQqzaklf9N&confirm=t", "dumps/clmm/modelExp1_Attitudes.rds")
}
modelExp1Attitudes = readRDS("dumps/clmm/modelExp1_Attitudes.rds")

clm_table(modelExp1Attitudes, caption="Table S3: Ordinal mixed-effects model of wordhood confidence ratings, taking into account attitudes and exposure. All numeric predictors are centered, and frequency bin is Helmert-coded.")
```

Table S3: Ordinal mixed-effects model of wordhood confidence ratings, taking into account attitudes and exposure. All numeric predictors are centered, and frequency bin is Helmert-coded.

|  | Parameter | Estimate | Std. Error | \(z\) | \(p\) |  |
| --- | --- | --- | --- | --- | --- | --- |
| Effects | score (centered) | 1.156 | 0.351 | 3.296 | <0.001 | \*\*\* |
|  | type = real | 0.472 | 0.095 | 4.988 | <0.001 | \*\*\* |
|  | freq = mid.v.low | 0.052 | 0.110 | 0.478 | 0.633 |  |
|  | freq = high.v.others | -0.069 | 0.099 | -0.697 | 0.486 |  |
|  | proSpanish (centered) | -0.070 | 0.079 | -0.885 | 0.376 |  |
|  | diacritic = TRUE | 1.176 | 0.269 | 4.371 | <0.001 | \*\*\* |
|  | nationalism (centered) | 0.102 | 0.058 | 1.764 | 0.078 | . |
|  | spanishExpo (centered) | 0.171 | 0.095 | 1.794 | 0.073 | . |
|  | type = real × freq = mid.v.low | 0.073 | 0.156 | 0.464 | 0.642 |  |
|  | type = real × freq = high.v.others | 0.534 | 0.147 | 3.629 | <0.001 | \*\*\* |
|  | score (centered) × proSpanish (centered) | 0.390 | 0.172 | 2.268 | 0.023 | \* |
|  | type = real × proSpanish (centered) | 0.164 | 0.047 | 3.479 | <0.001 | \*\*\* |
|  | freq = mid.v.low × proSpanish (centered) | -0.022 | 0.051 | -0.430 | 0.667 |  |
|  | freq = high.v.others × proSpanish (centered) | -0.072 | 0.046 | -1.558 | 0.119 |  |
|  | type = real × freq = mid.v.low × proSpanish (centered) | 0.132 | 0.073 | 1.805 | 0.071 | . |
|  | type = real × freq = high.v.others × proSpanish (centered) | 0.189 | 0.068 | 2.776 | 0.006 | \*\* |
| Thresholds | 1|2 | -2.751 | 0.145 |  |  |  |
|  | 2|3 | -0.964 | 0.139 |  |  |  |
|  | 3|4 | 0.503 | 0.139 |  |  |  |
|  | 4|5 | 2.831 | 0.144 |  |  |  |

```
# Plot model results for Exp1, taking into account attitude/exposure predictors

figS6a.mean <- clm_plotdat(modelExp1Attitudes, c("type", "freq", "proSpanish"), xlevels=list(proSpanish=c(-4, 4)), type="mean") %>%
  mutate(
    type = fct_recode(type, "Nonword"="pseudo", "Word"="real") %>% fct_relevel("Word"),
    attitude = factor(proSpanish, levels=c(4, -4), labels=c("Attitude: v. pos.", "Attitude: v. neg."))
  ) %>%
  ggplot(., aes(x=as.integer(freq), y=pred, color=type, fill=type, shape=type)) +
    geom_ribbon(aes(ymin=lci, ymax=uci), alpha=0.1, color=NA) +
    geom_line(size=1, alpha=0.4) +
    geom_point(size=4) +
    geom_errorbar(aes(ymin=lci, ymax=uci), size=1, width=0.3) +
    scale_x_continuous(name="Frequency Bin", breaks=1:3, labels=c("Low", "Mid", "High")) +
    ylab("Predicted mean rating") +
    # ylim(2.9, 4.1) +
    facet_grid(attitude ~ ., switch="y") +
    scale_shape_manual(values = c("Nonword" = 19, "Word" = 17)) +
    scale_color_manual(values = c("Nonword" = "black", "Word" = "blue")) +
    scale_fill_manual(values = c("Nonword" = "black", "Word" = "blue")) +
    theme_bw() +
    theme(
      legend.position="none",
      legend.title=element_blank()
    )

figS6a.dist <- clm_plotdat(modelExp1Attitudes, c("type", "freq", "proSpanish"), xlevels=list(proSpanish=c(-4, 4)), type="dist") %>%
  mutate(
    type = fct_recode(type, "Nonword"="pseudo", "Word"="real") %>% fct_relevel("Word"),
    attitude = factor(proSpanish, levels=c(4, -4), labels=c("Attitude: v. pos.", "Attitude: v. neg.")),
    Response = paste("Rating:", response)
  ) %>%
  ggplot(aes(x=as.integer(freq), y=pred, color=type, fill=type, shape=type)) +
    geom_ribbon(aes(ymin=lci, ymax=uci), alpha=0.2, color=NA) +
    geom_line(size=1, alpha=0.4) +
    geom_point(size=2) +
    geom_errorbar(aes(ymin=lci, ymax=uci), size=1, width=0.3) +
    facet_grid(attitude ~ Response, switch="y") +
    ylim(0, 1) +
    scale_x_continuous(name="Frequency Bin", breaks=1:3, labels=c("Low", "Mid", "High")) +
    ylab("Probability of rating") +
    scale_shape_manual(values = c("Nonword" = 19, "Word" = 17)) +
    scale_color_manual(values = c("Nonword" = "black", "Word" = "blue")) +
    scale_fill_manual(values = c("Nonword" = "black", "Word" = "blue")) +
    theme_bw() +
    theme(
      legend.position="right",
      legend.title=element_blank()
    )

figS6b.mean <- clm_plotdat(modelExp1Attitudes, c("score", "proSpanish"), xlevels=list(proSpanish=c(-4, 4), score=25), type="mean") %>%
  mutate(
    type="Word and\nNonword",
    attitude = factor(proSpanish, levels=c(4, -4), labels=c("Attitude: v. pos.", "Attitude: v. neg."))
  ) %>%
  ggplot(., aes(x=score, y=pred, color=type, fill=type)) +
    geom_ribbon(aes(ymin=lci, ymax=uci), alpha=0.3, color=NA) +
    geom_line(size=1) +
    scale_x_continuous(n.breaks=4) +
    xlab("Phonotactic score") +
    ylab("Predicted mean rating") +
    # ylim(2.9, 4.1) +
    facet_grid(attitude ~ ., switch="y") +
    scale_color_manual(values = c("Word and\nNonword" = "purple")) +
    scale_fill_manual(values = c("Word and\nNonword" = "purple")) +
    theme_bw() +
    theme(
      legend.position="none",
      legend.title=element_blank()
    )

figS6b.dist <- clm_plotdat(modelExp1Attitudes, c("score", "proSpanish"), xlevels=list(proSpanish=c(-4, 4), score=25), type="dist") %>%
  mutate(
    type="Word and\nNonword",
    attitude = factor(proSpanish, levels=c(4, -4), labels=c("Attitude: v. pos.", "Attitude: v. neg.")),
    Response = paste("Rating:", response)
  ) %>%
  ggplot(., aes(x=score, y=pred, color=type, fill=type)) +
    geom_ribbon(aes(ymin=lci, ymax=uci), alpha=0.3, color=NA) +
    geom_line(size=1) +
    scale_x_continuous(n.breaks=4) +
    xlab("Phonotactic score") +
    ylab("Probability of rating") +
    scale_color_manual(values = c("Word and\nNonword" = "purple")) +
    scale_fill_manual(values = c("Word and\nNonword" = "purple")) +
    facet_grid(attitude ~ Response, switch="y") +
    ylim(0, 1) +
    theme_bw() +
    theme(
      legend.position="right",
      legend.title=element_blank()
    )

figS6c.mean <- clm_plotdat(modelExp1Attitudes, "diacritic", type="mean") %>%
  mutate(
    type = "Word and\nNonword",
    diacritic = factor(diacritic, levels=c(TRUE, FALSE), labels=c("Present", "Absent"))
  ) %>%
  ggplot(., aes(x=diacritic, y=pred, color=type, fill=type)) +
    geom_point(size=4) +
    geom_errorbar(aes(ymin=lci, ymax=uci), size=1, width=0.3) +
    xlab("Non-English characters") +
    ylab("Predicted mean rating") +
    ylim(2.9, 4.1) +
    scale_color_manual(values = c("Word and\nNonword" = "purple")) +
    scale_fill_manual(values = c("Word and\nNonword" = "purple")) +
    theme_bw() +
    theme(
      legend.position="none",
      legend.title=element_blank()
    )

figS6c.dist <- clm_plotdat(modelExp1Attitudes, "diacritic", type="dist") %>%
  mutate(
    type = "Word and\nNonword",
    diacritic = factor(diacritic, levels=c(TRUE, FALSE), labels=c("Present", "Absent"))
  ) %>%
  ggplot(., aes(x=diacritic, y=pred, color=type, fill=type)) +
    geom_point(size=4) +
    geom_errorbar(aes(ymin=lci, ymax=uci), size=1, width=0.3) +
    xlab("Non-English characters") +
    ylab("Probability of rating") +
    scale_color_manual(values = c("Word and\nNonword" = "purple")) +
    scale_fill_manual(values = c("Word and\nNonword" = "purple")) +
    facet_grid(. ~ response, labeller=as_labeller(function(x) str_c("Rating: ", x))) +
    ylim(0, 1) +
    theme_bw() +
    theme(
      legend.position="right",
      legend.title=element_blank()
    )

ggarrange(figS6a.mean, figS6a.dist, figS6b.mean, figS6b.dist, figS6c.mean, figS6c.dist, ncol=2, labels=c("a.", "", "b.", "", "c.", ""), widths=c(1,5), heights=c(2,2,1))

remove(figS6a.mean, figS6a.dist, figS6b.mean, figS6b.dist, figS6c.mean, figS6c.dist)
```

Figure S6: Partial effect plots for ordinal mixed-effects model of wordhood confidence ratings, as affected by attitude toward Spanish (measured by value of Spanish and its speakers in the home state). Left-hand plots show predicted mean ratings; right-hand plots show predicted distributions over ratings (facets). Figure S6a (top) shows the interaction between frequency bin, lexicality (real words vs. nonwords), and attitude; Figure S5b (middle) shows the interaction between phonotactic score and attitude; Figure S5c (bottom) shows the effect of presence or absence of non-English characters. Error bars and ribbons represent 95% confidence intervals.

Below, we report Generalized Variance Inflation Factors (GVIFs) for the final model, which are calculated based on the extent to which each fixed effect predictor can be predicted from all other fixed effect predictors. Since this calculation is not dependent on the random effect structures, we perform it by applying the `vif()` function from the `car` package to a (fixed-effect) logit ordinal regression model with all of the fixed effects from the final ordinal regression model; since `vif()` is not applicable to models fit with the `ordinal` package at the time of writing, we instead apply it to a model fit with the `polr()` function from the `MASS` library.

Large GVIFs indicate multicollinearity, whereby one predictor is highly correlated with combinations of others, which decreases the extent to which inferences from the model can be trusted. However, GVIF scales exponentially with the degrees of freedom of the predictor; thus, raw GVIF values are not comparable. We thus additionally report *transformed* GVIF values which have been raised to the power of \(\frac{1}{df}\) in order to be comparable with each other (these are the squared values of `GVIF^(1/(2*Df))` reported by `vif()`; squaring puts these values on a scale which can be interpreted via the standard rule-of-thumb for VIFs). Transformed GVIFs above 5 are interpreted as potentially problematic, while transformed GVIFs above 10 are cause for serious concern.

For our model, all transformed GVIFs are below 5 and most are below 2: thus, even though there may be mild correlations in the data, they do not present much of a problem for using the model for inference.

```
clm_vif(modelExp1Attitudes, dataExp1, caption="Generalized Variance Inflation Factors for the final model assessing interactions with attitudes and exposure from Experiment 1. All numeric variables in this model are centered.")
```

Generalized Variance Inflation Factors for the final model assessing interactions with attitudes and exposure from Experiment 1. All numeric variables in this model are centered.

| Parameter | GVIF | df | Transformed GVIF |
| --- | --- | --- | --- |
| score (centered) | 1.039 | 1 | 1.039 |
| type | 1.008 | 1 | 1.008 |
| freq | 3.823 | 2 | 1.955 |
| proSpanish (centered) | 2.254 | 1 | 2.254 |
| diacritic | 1.028 | 1 | 1.028 |
| nationalism (centered) | 1.068 | 1 | 1.068 |
| spanishExpo (centered) | 1.348 | 1 | 1.348 |
| type × freq | 3.827 | 2 | 1.956 |
| score (centered) × proSpanish (centered) | 1.021 | 1 | 1.021 |
| type × proSpanish (centered) | 1.970 | 1 | 1.970 |
| freq × proSpanish (centered) | 3.899 | 2 | 1.974 |
| type × freq × proSpanish (centered) | 3.894 | 2 | 1.973 |

# 3 Experiment 2: Well-formedness rating task

In Experiment 2, non-Spanish-speaking participants assigned well-formedness ratings to nonwords with varying phonotactic scores.

We provide an overview of participants’ demographics below; for further details of participants, including exclusion criteria, see Detailed Materials and Methods Section 1 (especially 1.2). For details of the stimuli, see Detailed Materials and Methods Section 2 (especially 2.4). For details of the procedure, see Detailed Materials and Methods Section 3 (especially 3.2).

```
# Load data for Exp2 and filter participants

dataExp2 = read.csv("../data/phonotactic-rating_responses.csv", header = TRUE, encoding = "UTF-8")
dataExp2$txt = as.character(dataExp2$txt)

# Set up factor levels
dataExp2$age = factor(dataExp2$age, levels=c("18-29", "30-39", "40-49", "50-59", ">60"))
dataExp2$education = factor(dataExp2$education, levels=c("highSchool", "2yrCollege", "4yrCollege", "grad"))

# Remove trials for items identified as problematic after the experiment:
# items that are orthographically identical to English words
# and items that have a score of lower than -1.3
dataExp2 = dataExp2 %>%
  filter(!english, score > -1.3) %>%
  # Also remove trials where a participant saw the same phonological form twice (with and without accent)
  anti_join(
    dataExp2 %>%
      group_by(workerId, phon) %>%
      tally() %>%
      ungroup() %>%
      filter(n > 1),
    by = c("workerId", "phon")
  )
  
# Load participants list, for ease of tracking participant-level exclusions
participantsExp2 = read.csv("../data/phonotactic-rating_workers.csv", header = TRUE)
participantsExp2$age = factor(participantsExp2$age, levels=c("18-29", "30-39", "40-49", "50-59", ">60"))
participantsExp2$education = factor(participantsExp2$education, levels=c("highSchool", "2yrCollege", "4yrCollege", "grad"))

participantsExp2 = participantsExp2 %>%
  filter(!(
    numNone > 3  # Remove participants who skipped more than 3 trials (7 removed)
    | str_detect(otherLang, fixed("Spanish"))  # Remove participants who list Spanish as a language they speak well (13 removed)
    | livedSpanish  # Remove participants who have lived in a Spanish-speaking country (0 removed)
    | str_detect(spanishEd, fixed("grad"))  # Remove participants who have studied Spanish at college level (20 removed)
    | speakSpanish >= 3 | understandSpanish >= 3  # Remove participants whose Spanish speaking / comprehension scores are 3 or higher (6 removed)
    | romanceLang  # Remove participants who speak another Romance language (1 removed)
    | livedRomance  # Remove participants who have lived in a country in which a Romance language is widely spoken (1 removed)
    | numMode > 180  # Remove participants who used a single rating for more than 180/240 trials (4 removed)
  ))

# Check the total number of usable participants for Exp2 (39 usable participants)
# nrow(participantsExp2)

# Copy participant-level exclusions to trials
dataExp2 = dataExp2 %>%
  semi_join(
    participantsExp2,
    by="workerId"
  )

# Filter individual trials
dataExp2 = dataExp2 %>%
  filter(!(
    is.na(response)  # Remove trials with no response (7 removed)
    | rt_ms < 250  # Remove trials with RT faster than 250ms (1 removed)
  ))

# Check the total number of usable trials for Exp2 (9298 usable trials)
# nrow(dataExp2)


# Get the by-item summary
itemsExp2 = dataExp2 %>%
  group_by(txt, phon, length, score, scoreMorphsParsed, scoreMorphsUnparsed, scoreEngOrth, scoreSpOrth, diacritic) %>%
  summarise(
    mean_response = mean(response),
    n = n()
  ) %>%
  ungroup()
```

## 3.1 Dataset structure

The dataset is structured as follows:

- *workerId* is the unique ID for each participant.
- *ip\_region* is the region from which the participant’s IP address was logged.
- *state* is the State that the participant indicated they grew up in and currently reside in.
- *time\_outside\_state* is the longest period of time the participant has spent outside of their State since the age of 7.
- *spanishEd* is the highest level of education at which the participant has studied Spanish.
- *speakSpanish* is the participant’s rating of how well they can speak Spanish (with a scale ranging from 0 to 5).
- *understandSpanish* is the participant’s rating of how well they can understand/read Spanish (with a scale ranging from 0 to 5).
- *spanishProf* is the sum of quantified response for speakSpanish and understandSpanish, which refers to the level of the participant’s Spanish proficiency (results can range from 0 to 10).
- *age* is the age group that the participant belongs to.
- *gender* is the gender of the participant (grouped into categories).
- *ethnicity* is the ethnicity of the partipant (grouped into categories based on free responses).
- *politics* is the political party with which the participant identifies most strongly.
- *education* is the participant’s highest level of education.
- *basicSpanish* is the basic elements of Spanish that the participant is able to use.
- *basicSpanishCount* is the number of basic elements of Spanish that the participant is able to use(results can range from 0 to 8).
- *spanishExpoMedia* is a log-like estimate of how often the participant is exposed to Spanish through the media (results can range from 1 to 5).
- *spanishExpoSocial* is a log-like estimate of how often the participant is exposed to Spanish through socializing (results can range from 1 to 5).
- *spanishExpo* is the sum of quantified response for spanishExpoMedia and spanishExpoSocial, which refers to the level of the participant’s overall exposure to Spanish (results can range from 2 to 10).
- *otherLang* is a list of the other languages that the participant can speak well.
- *romanceLang* indicates whether the participant can speak or understand any other Romance languages, even at a basic level.
- *romanceLang\_specify* is a list of the other Romance languages that the participant can speak or understand.
- *livedSpanish* indicates whether the particpant has lived in a country outside the US where Spanish is predominantly spoken.
- *livedSpanish\_specify* is a list of the Spanish-speaking countries in which the participant has lived.
- *livedRomance* indicates whether the particpant has lived in a country outside the US where a Romance language is predominantly spoken.
- *livedRomance\_specify* is a list of the Romance-language-speaking countries in which the participant has lived.
- *proSpanishLang* is the participant’s level of agreement with the statement: “Some Spanish language education should be compulsory in school for all children in ” (responses can range from 1 to 5).
- *proSpanishCult* is the participant’s level of agreement with the statement: “Hispanic and Latino cultures are important in ” (responses can range from 1 to 5).
- *proSpanish* is a measure of how strongly the participant values Spanish language and culture in their home state, obtained by adding the quantified responses to proSpanishLang and proSpanishCult and subtracting 6 (results can range from -4 to 4; positive numbers indicate a positive attitude toward Spanish). This variable is referred to as *Spanish value* in the paper.
- *proEnglishUSA* is the participant’s level of agreement with the statement: “People in the United States should speak English, not foreign languages” (responses can range from 1 to 5).
- *proImmigration* is the participant’s completion of the statement: “I think that the number of immigrants from foreign countries who are permitted to come to the United States to live should be \_\_\_\_\_” (responses can range from 1 to 5, with scores below 3 indicating decrease in immigration and scores above 3 indicating increase in immigration).
- *nationalism* is a measure of the strength of the participant’s nationalist feelings toward the US, obtained by subtracting the quantified response to proImmigration from the quantified response to proEnglishUSA (results can range from -4 to 4; po
- *txt* is the orthographic form of an item, presented as a stimulus.
- *phon* is the phonological form of the item.
- *length* is the phoneme length of the item.
- *score* is the word-based phonotactic score for the item, based on all words in the lexicon.
- *score15000* is the word-based phonotactic score for the item, based on an average over frequency-weighted samples of 15,000 words from the lexicon. This is the best-performing word-based phonotactic score are able to identify at present.
- *scoreMorphsParsed* is the morph-based phonotactic score for the item, assuming participants are parsing stimuli into morphs, based on all morphs in the lexicon.
- *scoreMorphsParsed12000* is the morph-based phonotactic score for the item, assuming participants are parsing stimuli into morphs, based on an average over frequency-weighted samples of 12,000 morphs from the lexicon. This is the best-performing morph-based phonotactic score are able to identify at present.
- *scoreMorphsUnparsed* is the morph-based phonotactic score for the item, assuming participants are not parsing stimuli into morphs, based on all morphs in the lexicon.
- *scoreSpOrth* is the (word-based) score for the item according to Spanish orthotactics (ignoring diacritics), based on the same set of word types use for phonotactic scores.
- *scoreEngOrth* is the score for the item according to English orthotactics (ignoring diacritics), based on word types without non-alphabetic characters that occur in the CMU Pronouncing Dictionary.
- *english* is whether the item is orthographically identical to an English word.
- *diacriticEnglish* is whether the item would be orthographically identical to an English word as above, except that it contains a diacritic that is not typical in English words: <á, é, í, ó, ú, ñ>. Such items are *not* removed from our analysis, but are tagged for further study.
- *diacritic* is whether or not the orthographic item contains a diacritic that is not typical in English words: <á, é, í, ó, ú, ñ>
- *response* is the phonotactic wellformedness rating a participant gave for a stimulus.
- *rt\_ms* is the reaction time for the response (in milliseconds).

As previously described, the formulations of *proSpanish* and *nationalism* are by design, but have also been confirmed by exploratory factor analysis of responses to the 4 attitude questions across all unique participants analyzed in either experiment (see Appendix C of this supplement for details). We choose to use the manually-designed formulations in our analysis, rather than the inferred factors, in order to ensure that the formulation is independent of the precise set of participants who took the experiment.

## 3.2 Overview of participants’ demographics

Figure S7a summarizes the distribution of participants on demographic and linguistic axes.

```
# Plot overview of participants in Exp2

# Recode ethnicity factor to fit on axis labels
participantsExp2 = participantsExp2 %>%
  mutate(
    ethnicity = fct_recode(ethnicity, 
                           "Asian"="Asian",
                           "Black"="Black",
                           "Hisp. /\nLatinx"="Hispanic",
                           "Native\nAmerican" = "Native American",
                           "not\nspec." = "not specified",
                           "White"="White",
                           "White &\nAsian" = "White & Asian"
                           )
  )

plots_specs = list(
  basicSpanishCount = list(xlab="Number of elements known", title="Knowledge of basic Spanish elements"),
  spanishProf = list(xlab="Spanish proficiency rating", title="Degree of Spanish proficiency"),
  spanishExpo = list(xlab="Spanish exposure rating", title="Degree of exposure to Spanish"),
  gender = list(xlab="Gender", title="Gender"),
  age = list(xlab="Age group", title="Age"),
  education = list(xlab="Highest education", title="Education"),
  state = list(xlab="Home state", title="State"),
  ethnicity = list(xlab="Ethnicity", title="Ethnicity"),
  politics = list(xlab="Political party most identified with", title="Political affiliation"),
  proSpanish = list(xlab="Score of attitude toward Spanish\nbased on ratings of language and culture", title="Spanish attitude"),
  nationalism = list(xlab="Score of nationalism\nbased on ratings of immigration and English", title="Nationalism")
)

# Make plots
plots_list = imap(plots_specs, 
                  function(fields, varname) {
                    return(make_count_plot(participantsExp2, varname, fields$xlab, "Number of participants", fields$title))
                  })

ggarrange(plots=plots_list, ncol=3)

remove(plots_specs, plots_list)
```

Figure S7a: Overview of participants’ demographics in Exp2.

Figure S7b shows correlations between the key individual difference predictors of *Spanish value*, *nationalism*, and *exposure* for participants in Exp2. We quantified these correlations using the `polychor()` function in the `polycor` package, as follows:

- As in Exp1, there is a small negative polychoric correlation between *Spanish value* and *nationalism* (\(\rho=-0.147\)): participants with high Spanish value tend to have low nationalism, but there are also many participants with above-zero Spanish value and above-zero nationalism.
- As in Exp1, there is a positive polychoric correlation between *Spanish value* and *Spanish exposure*, but it is smaller here (\(\rho=0.246\)): participants with high Spanish value tend to have more exposure to Spanish than others, while participants with very low Spanish value tend to have very little Spanish exposure, but there is still a lot of variation in degrees of exposure across Spanish value levels.
- Finally, there is a negligible polychoric correlation between *nationalism* and *Spanish exposure* (\(\rho=0.032\)): participants with all degrees of nationalism display variation in their degrees of Spanish exposure. As in Exp1, the data visualization suggests a non-linear relationship between nationalism and Spanish exposure, where participants with extreme nationalism scores (either positive or negative) tend not to have as much Spanish exposure as participants with near-zero nationalism scores; however, this pattern is less striking here than in Exp1.

To ensure that these correlations do not create harmful multicollinearity, we report Variance Inflation Scores when we model the effects of attitudes and exposure.

```
corplot_a = participantsExp2 %>%
  dplyr::count(across(c(proSpanish, nationalism)), .drop=FALSE) %>%
  ggplot(., aes(x=proSpanish, y=nationalism)) +
  geom_hline(yintercept=0, linetype="dashed", color="darkgray", size=2) +
  geom_vline(xintercept=0, linetype="dashed", color="darkgray", size=2) +
  geom_smooth(data=participantsExp1, method="loess", formula="y~x", alpha=0.3) +
  geom_point(aes(size=n)) +
  scale_size_continuous(name="Participants", range=c(3,7), breaks=1:3) +
  xlab("Spanish value") +
  ylab("Nationalism") +
  coord_cartesian(xlim=c(-4, 4), ylim=c(-4, 4)) +
  theme_bw() +
  theme(
    panel.grid = element_blank(),
    axis.title = element_text(size=16, color="black"),
    axis.text = element_text(size=14, color="black"),
    legend.title = element_text(size=16, color="black"),
    legend.text = element_text(size=15, color="black")
  )

corplot_b = participantsExp2 %>%
  dplyr::count(across(c(proSpanish, spanishExpo)), .drop=FALSE) %>%
  ggplot(., aes(x=proSpanish, y=spanishExpo)) +
  geom_smooth(data=participantsExp1, method="loess", formula="y~x", alpha=0.3) +
  geom_point(aes(size=n)) +
  scale_size_continuous(name="Participants", range=c(3,9), breaks=1:7) +
  xlab("Spanish value") +
  ylab("Spanish exposure") +
  coord_cartesian(xlim=c(-4, 4), ylim=c(2, 10)) +
  theme_bw() +
  theme(
    panel.grid = element_blank(),
    axis.title = element_text(size=16, color="black"),
    axis.text = element_text(size=14, color="black"),
    legend.title = element_text(size=16, color="black"),
    legend.text = element_text(size=15, color="black")
  )

corplot_c = participantsExp2 %>%
  dplyr::count(across(c(nationalism, spanishExpo)), .drop=FALSE) %>%
  ggplot(., aes(x=nationalism, y=spanishExpo)) +
  geom_smooth(data=participantsExp1, method="loess", formula="y~x", alpha=0.3) +
  geom_point(aes(size=n)) +
  scale_size_continuous(name="Participants", range=c(3,7), breaks=1:5) +
  xlab("Nationalism") +
  ylab("Spanish exposure") +
  coord_cartesian(xlim=c(-4, 4), ylim=c(2, 10)) +
  theme_bw() +
  theme(
    panel.grid = element_blank(),
    axis.title = element_text(size=16, color="black"),
    axis.text = element_text(size=14, color="black"),
    legend.title = element_text(size=16, color="black"),
    legend.text = element_text(size=15, color="black")
  )

null_plot = ggplot() + theme_void()

ggarrange(plots=list(corplot_a, null_plot, corplot_b, null_plot, corplot_c), ncol=1, heights=c(1, 0.1, 1, 0.1, 1))

remove(corplot_a, corplot_b, corplot_c, null_plot)
```

Figure S7b: Correlations between participants’ Spanish value, nationalism, and Spanish exposure in Exp2.

## 3.3 Visualization of raw data

Nonwords with high phonotactic scores appear to receive higher well-formedness ratings than nonwords with low phonotactic scores. Additionally, nonwords that include at least one non-English character appear to receive higher scores than those that don’t include any non-English characters (but the effect of phonotactic score seems to be of similar strength for both groups).

```
# Plot raw data for Exp2, by phonotactic score and presence of non-English characters

ggplot(itemsExp2, aes(x=score, y=mean_response)) +
  geom_point(shape=1, alpha=0.3, size=4) +
  geom_smooth(method="lm", formula="y~x", alpha=0.6, size=2, color="black") +
  facet_grid(. ~ diacritic, labeller=labeller(diacritic=c("FALSE"="Has English characters only", "TRUE"="Has non-English character(s)"))) +
  labs(y = "Nonword wellformedness\nMean rating (per stimulus)", x = "Phonotactic score") +
  theme_bw() +
  ylim(1, 5) +
  theme(
    panel.grid = element_blank(),
    axis.title = element_text(size=16, color="black"),
    axis.text = element_text(size=14, color="black"),
    legend.title = element_text(size=16, color="black"),
    legend.text = element_text(size=15, color="black"),
    strip.text = element_text(size=15, color="black")
  )
```

Figure S8: Mean phonotactic well-formedness ratings for each nonword stimulus. Points represent mean ratings for each unique nonword.

## 3.4 Statistical analysis

We first present an analysis testing whether participants’ ratings of phonotactic wellformedness are sensitive to phonotactic probability, calculated over word types.

Then, we follow Oh et al. (2020) in conducting a series of analyses to investigate the source of the knowledge that underpins participants’ wellformedness ratings. We start from the assumption that phonotactic knowledge derives from implicit knowledge of words, and we conduct Monte Carlo simulations to assess how many words participants would need to know in order to best model their wellformedness ratings. We then ask whether participants might actually know *parts* of words (*morphs*) rather than words themselves, and we conduct Monte Carlo simulations to assess how many morphs participants would need to know in order to best model their wellformedness ratings. Finally, we compare the word-based and morph-based models, to determine the best source for modeling phonotactic knowledge in our non-Spanish-speaking participants. See Detailed Materials and Methods, Section 4, for details of phonotactic scoring.

At each stage of the analysis, we compare a variety of ordinal regression models that are identical apart from the phonotactic scores they employ, and we select the model (and corresponding scores) with lowest AIC. Each model contains a predictor for phonotactic score (centered), as well as a predictor for the presence or absence of non-English characters. Except for in Monte Carlo analyses, we use mixed-effects ordinal regression models, with random intercepts for participant and item, and random slopes by participant for phonotactic score and non-English characters. In Monte Carlo analyses, we use fixed-effects ordinal regression models for practical reasons; these fixed-effects models differ from the mixed-effects models only in omitting the random effects (see Detailed Materials and Methods, Section 5.3, for more information about the Monte Carlo analyses).

To summarize, the analysis unfolds over several stages, asking the following questions:

1. Do non-Spanish-speaking participants show evidence of phonotactic knowledge of Spanish, through wellformedness ratings that are sensitive to phonotactic probability?
2. How many words do participants need to have knowledge of? *(Monte Carlo analysis)*
3. Could phonotactic knowledge be underpinned by morphs, i.e. units that could be smaller than words?
4. How many morphs do participants need to have knowledge of, and do participants attempt to parse stimuli into morphs in order to evaluate them phonotactically? *(Monte Carlo analysis)*
5. Once and for all, what is the source of phonotactic knowledge that best explains participants’ well-formedness ratings?

After Stage 5, we consider the role of self-reported attitudes (Spanish value and nationalism) and exposure in the best-fitting model of wellformedness ratings.

### 3.4.1 Stage 1: Do non-Spanish speakers have phonotactic knowledge?

In the first stage of the analysis, we ask whether participants show sensitivity to phonotactic probability in the assignment of well-formedness ratings. We construct a mixed-effects ordinal regression model, attempting to predict the well-formedness ratings that participants gave to nonwords from the phonotactic scores of those nonwords. The model uses random intercepts for participant and item, and random by-participant slopes for phonotactic score.

Informed by the results of Exp1 and visualization of the raw data, we include a predictor for the presence or absence of non-English characters, together with its own by-participant random slope. We construct two models: one in which this predictor interacts with phonotactic score (where there is also a by-participant random slope for the interaction term), and one in which there is no interaction. We compare the models and move forward with the one that has lower AIC, which is the model that lacks the interaction term.

In this model, phonotactic scores have a statistically significant (positive) effect, which we take as an indication of participants’ possession and use of fine-grained phonotactic knowledge of Spanish. The model is presented in Table S4, and partial effects plots are presented in Figure S9.

```
# Stage 1 analysis of Exp2: evidence of phonotactic knowledge

dataExp2$response <- as.factor(dataExp2$response)

# m1 <- clmm(
#   response ~
#     c.(score) * diacritic +
#     (1 + c.(score) * diacritic | workerId) + (1 | phon),
#   data=dataExp2
# )
# 
# m2 <- clmm(
#   response ~
#     c.(score) + diacritic +
#     (1 + c.(score) + diacritic | workerId) + (1 | phon),
#   data=dataExp2
# )
# 
# anova(m1, m2) # Reduced model has lower AIC (and also no significant difference based on log-likelihood test), so accept
# 
# saveRDS(m2, file = "dumps/clmm/modelExp2_WordTypes.rds")
# remove(m1, m2)

modelWordTypes <- readRDS("dumps/clmm/modelExp2_WordTypes.rds")

clm_table(modelWordTypes, caption="Table S4: Ordinal mixed-effects model summary for well-formedness ratings. Phonotactic score is centered.")
```

Table S4: Ordinal mixed-effects model summary for well-formedness ratings. Phonotactic score is centered.

|  | Parameter | Estimate | Std. Error | \(z\) | \(p\) |  |
| --- | --- | --- | --- | --- | --- | --- |
| Effects | score (centered) | 2.178 | 0.427 | 5.105 | <0.001 | \*\*\* |
|  | diacritic = TRUE | 0.884 | 0.254 | 3.478 | <0.001 | \*\*\* |
| Thresholds | 1|2 | -2.478 | 0.125 |  |  |  |
|  | 2|3 | -1.016 | 0.122 |  |  |  |
|  | 3|4 | 0.044 | 0.122 |  |  |  |
|  | 4|5 | 1.858 | 0.123 |  |  |  |

```
# Plot of stage 1 analysis of Exp2: evidence of phonotactic knowledge

fig.9a.mean <- clm_plotdat(modelWordTypes, c("score"), xlevels=list(score=25), type="mean") %>%
  ggplot(., aes(x=score, y=pred)) +
  geom_ribbon(aes(ymin=lci, ymax=uci), alpha=0.2, color=NA, fill="black") + 
  geom_line(size=1, color="black") +
  xlab("Phonotactic score") +
  ylab("Predicted mean rating") + 
  ylim(2.7, 4.1) +
  theme_bw() + 
  theme(
    legend.position="none"
  )

fig.9a.dist <- clm_plotdat(modelWordTypes, c("score"), xlevels=list(score=25), type="dist") %>%
  ggplot(., aes(x=score, y=pred)) +
  geom_ribbon(aes(ymin=lci, ymax=uci), alpha=0.2, color=NA, fill="black") + 
  geom_line(size=1, color="black") +
  scale_x_continuous(n.breaks=4) +
  xlab("Phonotactic score") +
  ylab("Probability of rating") +    
  facet_grid(. ~ response, labeller=as_labeller(function(x) str_c("Rating: ", x))) +
  ylim(0, 1) +
  theme_bw() + 
  theme(
    legend.position="none"
  )

fig.9b.mean <- clm_plotdat(modelWordTypes, c("diacritic"), type="mean") %>%
  mutate(
    diacritic = factor(diacritic, levels=c(TRUE, FALSE), labels=c("Present", "Absent"))
  ) %>%
  ggplot(., aes(x=diacritic, y=pred)) +
  geom_point(size=4, color="black") +
  geom_errorbar(aes(ymin=lci, ymax=uci), size=1, width=0.3, color="black") +
  xlab("Non-English characters") +
  ylab("Predicted mean rating") + 
  ylim(2.7, 4.1) +
  theme_bw() + 
  theme(
    legend.position="none"
  )

fig.9b.dist <- clm_plotdat(modelWordTypes, c("diacritic"), type="dist") %>%
  mutate(
    diacritic = factor(diacritic, levels=c(TRUE, FALSE), labels=c("Present", "Absent"))
  ) %>%
  ggplot(., aes(x=diacritic, y=pred)) +
  geom_point(size=4, color="black") +
  geom_errorbar(aes(ymin=lci, ymax=uci), size=1, width=0.3, color="black") +
  xlab("Non-English characters") +
  ylab("Probability of rating") +    
  facet_grid(. ~ response, labeller=as_labeller(function(x) str_c("Rating: ", x))) +
  ylim(0, 1) +
  theme_bw() + 
  theme(
    legend.position="none"
  )

ggarrange(fig.9a.mean, fig.9a.dist, fig.9b.mean, fig.9b.dist, ncol=2, labels=c("a.", "", "b.", ""), widths=c(1,5))

remove(fig.9a.mean, fig.9a.dist, fig.9b.mean, fig.9b.dist)
```

Figure S9: Partial effect plots for ordinal mixed-effects model of phonotactic well-formedness of nonwords. Left-hand plots show predicted mean ratings; right-hand plots show predicted distributions over ratings (facets). Figure S9a (top) shows the effect of phonotactic score; Figure S9b (bottom) shows the effect of presence or absence of non-English characters. Error bars and ribbons represent 95% confidence intervals.

### 3.4.2 Stage 2: How many words do we need?

In the second stage of the analysis, we ask how many word types underpin the phonotactic knowledge evidenced by participants, in order to evaluate how many words they would need to ‘know’ (i.e. have in their proto-lexicon) in order to best match the observed behavior. We conduct Monte Carlo analyses that compare fixed-effects ordinal regression models, using phonotactic scores based on different numbers of word types. We consider two different ways of sampling a *vocabulary* consisting of a fixed number of words (see Detailed Materials and Methods, Section 5.3, for details):

- **Unweighted**: samples types uniformly at random, regardless of frequency.
- **Frequency-weighted**: samples types proportional to their frequency.

The results are visualized in Figure S10. Models suffer no substantial increase in AIC (i.e. increase of larger than 4 AIC points) with the use of phonotactic scores based on a subset of approximately 12,000 words, sampled in proportion to frequency.

```
# Stage 2 analysis of Exp2: MC analysis for word-based proto-lexicon size
# Note: this section of the code can be re-run, but it will take a long time and create a lot of files in the directory denoted by the variable "container".
# Use of the scripts requires SRILM to be installed.
# On a Windows machine, use of the scripts additionally requires a POSIX shell (e.g. cygwin) to be installed, with the corresponding env set to the environment variable R_SHELL.

vocabsizes = c(seq(from=500, to=4500, by=500), seq(from=5000, to=14000, by=1000), seq(from=15000, to=45000, by=5000), seq(from=50000, to=90000, by=10000))
numsamples = 1000

container <- "EXCLUDED" # Set to "." for current directory

# Set up file structure
dir.create(paste(container, "MC_words", sep="/"))
for (scheme in c("unweighted", "freq-weighted")) {
  dir.create(paste(container, "MC_words", scheme, sep="/"))
  for (subdir in c("samples", "auxiliaries", "phonotactic-models", "probs")) {
    dir.create(paste(container, "MC_words", scheme, subdir, sep="/"))
    for (vocabsize in vocabsizes) {
      dir.create(paste(container, "MC_words", scheme, subdir, vocabsize, sep="/"))
    }
  }
}
remove(subdir)

# Step 1: get samples
subtlex <- read.csv("spanish_lexicon-and-freq.txt", sep="\t", header=TRUE, stringsAsFactors=FALSE, encoding="UTF-8") %>%
  dplyr::select("word"=phones, count) %>%
  mutate(
    word = gsub("(.)", "\\1 ", word) %>% trimws(.)
  )
set.seed(1234)

for (vocabsize in vocabsizes) {
  for (samplenum in 1:numsamples) {
    
    # Unweighted sample
    sample(subtlex$word, vocabsize, replace=FALSE) %>%
      write.table(., 
                  paste(container, "MC_words/unweighted/samples", 
                        vocabsize, 
                        paste0("sample_", samplenum, ".txt"), 
                        sep="/"), 
                  row.names=FALSE, col.names=FALSE, quote=FALSE)
    
    # Frequency-weighted sample
    sample(subtlex$word, vocabsize, replace=FALSE, prob=subtlex$count) %>%
      write.table(., 
                  paste(container, "MC_words/freq-weighted/samples", 
                        vocabsize, 
                        paste0("sample_", samplenum, ".txt"), 
                        sep="/"), 
                  row.names=FALSE, col.names=FALSE, quote=FALSE)
    
  }    
}
remove(subtlex)

# Step 2: calculate phonotactic probabilities, using the score-unparsed.sh shell script
setwd("scripts")
for (scheme in c("unweighted", "freq-weighted")) {
  for (vocabsize in vocabsizes) {
    for (samplenum in 1:numsamples) {
      shellCommand <- paste0(
        "sh score-unparsed.sh",
        " ../", container, "/MC_words/", scheme, "/samples/", vocabsize, "/sample_", samplenum, ".txt",
        " stimuli.txt",
        " ../", container, "/MC_words/", scheme, "/phonotactic-models/", vocabsize,
        " ../", container, "/MC_words/", scheme, "/auxiliaries/", vocabsize,
        " ../", container, "/MC_words/", scheme, "/probs/", vocabsize
      )
      run_shell(shellCommand)
    }
  }
}
setwd("..")
remove(shellCommand)

# Step 3: compute fixed-effects ordinal regression models and save the AIC values
dataExp2$response <- as.factor(dataExp2$response)
AIC_words = data.frame(
  scheme = rep(c("unweighted", "freq-weighted"), each=length(vocabsizes)*numsamples),
  vocabsize = rep(rep(vocabsizes, each=numsamples), 2),
  samplenum = rep(1:numsamples, 2*length(vocabsizes)),
  aic = rep(NA, 2*length(vocabsizes)*numsamples)
)
for (scheme in c("unweighted", "freq-weighted")) {
  for (vocabsize in vocabsizes) {
    aic_values = rep(NA, numsamples)
    for (samplenum in 1:numsamples) {
      mod <- clm(
        response ~ c.(scoreSample) + diacritic,
        data=add_scores(dataExp2, paste0(container, "/MC_words/", scheme, "/probs/", vocabsize, "/sample_", samplenum, ".csv"), "scoreSample")
      )
      aic_values[samplenum] <- AIC(mod)
    }
    AIC_words[AIC_words$scheme==scheme & AIC_words$vocabsize==vocabsize, "aic"] <- aic_values
  }
}

write.table(AIC_words, file="dumps/monte-carlo/AIC_words.txt", row.names=FALSE, quote=FALSE, sep="\t")
remove(AIC_words, aic_values, mod, samplenum, vocabsize, numsamples, scheme, container)
```

```
# Plot of stage 2 analysis of Exp2: MC analysis for word-based proto-lexicon size

figS10dat <- read.delim("dumps/monte-carlo/AIC_words.txt", sep="\t", header=TRUE) %>%
  group_by(scheme, vocabsize) %>%
  summarise(
    mean.AIC = mean(aic),
    lower.AIC = quantile(aic, probs=c(0.025)),
    upper.AIC = quantile(aic, probs=c(0.975))
  ) %>%
  ungroup()

# Get the AIC values for the full lexicon, in a fixed-effects setting
# These are preserved as a baseline for the morph-based Monte Carlo analysis to come
dataExp2$response <- as.factor(dataExp2$response)
fullLexAIC <- clm(
  response ~ c.(score) + diacritic,
  data=dataExp2
) %>%
  AIC(.)

ggplot(figS10dat, aes(x=vocabsize, y=mean.AIC, ymin=lower.AIC, ymax=upper.AIC, color=scheme, fill=scheme)) +
  geom_errorbar(position=position_dodge(width=200), size=1) +
  geom_line(position=position_dodge(width=200), size=2, alpha=0.5) +
  geom_hline(aes(yintercept=fullLexAIC, linetype="All words (93.8k)"), color="black", size=2) +   
  geom_point(shape=21, color="black", position=position_dodge(width=200), size=3) +
  labs(
    # title="Word-based Monte Carlo analyses",
    x="Vocabulary size", 
    y="AIC score"
  ) + 
  scale_x_continuous(
    breaks=seq(from=10000, to=90000, by=10000),
    labels=function(b){return(paste0(b/1000,"k"))}
    ) +
  scale_color_manual(
    name="Sampling", 
    breaks=c("unweighted", "freq-weighted"), 
    values=c("dodgerblue3", "firebrick3"),
    labels=c("Unweighted", "Frequency-weighted")
  ) +
  scale_fill_manual(
    name="Sampling", 
    breaks=c("unweighted", "freq-weighted"), 
    values=c("dodgerblue3", "firebrick3"),
    labels=c("Unweighted", "Frequency-weighted")
  ) +
  scale_linetype_manual(name=NULL, values=c("All words (93.8k)"="dashed")) +
  guides(color=guide_legend(order=1), fill=guide_legend(order=1), linetype=guide_legend(order=2)) +
  theme_bw() + 
  theme(
    plot.title=element_text(hjust=0.5, size=18),
    axis.title = element_text(size=16, color="black"),
    axis.text = element_text(size=14, color="black"),
    legend.title = element_text(size=16, color="black"),
    legend.text = element_text(size=15, color="black"),
    strip.text = element_text(size=15, color="black")
  )

remove(figS10dat)
```

Figure S10: Monte Carlo analyses with 1,000 random samples over 31 vocabulary sizes. Error bars represent 95% bootstrap percentile intervals.

The Monte Carlo analysis provides us with an idea of the best vocabulary size, but because it only uses fixed-effects regression models, its results may be overly influenced by particular participants or stimuli. To get a better sense of the number of words that we need, we need to conduct mixed-effects modeling. However, we can’t use the Monte Carlo approach, because it is computationally infeasible to fit so many mixed-effects models. To get a more informative sense of the best vocabulary size, we develop a *single* representative phonotactic score system for each vocabulary size, which we compare with mixed-effects regression models.

We base our representative phonotactic scores on the frequency-weighted sampling scheme. To collapse the 1000 different phonotactic score systems at each vocabulary size into a single score system, we convert the 1000 scores for each stimulus to probabilities (via exponentiation), take the means of these probabilities, and then convert them back to scores (via log-transformation). The resultant scores represent an aggregate-level average of a situation in which participants may all possess slightly different proto-lexicons of the same size, taking into account the probability of a participant having a particular proto-lexicon.

Based on our previous results, we compare vocabulary sizes up to 25,000, since that is the point at which the frequency-weighted sampling scheme starts to increase in AIC again. The results of the mixed-effects analysis are presented in Table S5. The lowest AIC comes from the use of averaged phonotactic scores based on a proto-lexicon of 15,000 words. These phonotactic scores are preserved in the original dataset as `score15000`.

```
# Stage 2 analysis of Exp2 continued: MC analysis for word-based proto-lexicon size

vocabsizes = c(seq(from=500, to=4500, by=500), seq(from=5000, to=14000, by=1000), seq(from=15000, to=45000, by=5000), seq(from=50000, to=90000, by=10000))
vocabsizes = vocabsizes[vocabsizes <= 25000]

# container <- "EXCLUDED" # Set to "." for current directory
# 
# scoresExp2 = itemsExp2 %>%
#   dplyr::select(phon, score) %>%
#   distinct()
# 
# for (vocabsize in vocabsizes) {
#   path = paste0(container, "/MC_words/freq-weighted/probs/", vocabsize, "/")
#   scoreName = paste0("score", vocabsize)
#   scoresExp2 = scoresExp2 %>%
#     left_join(
#       logMeanExp_scores(path, 1000) %>%
#         rename(!!scoreName := scoreAverage),
#       by = "phon"
#     )
# }
# 
# write.table(scoresExp2, file="dumps/monte-carlo/logMeanExp-scores_words.txt", row.names=FALSE, quote=FALSE, sep="\t")
# remove(scoresExp2, container, vocabsize, path, scoreName)

scoresExp2 = read.delim("dumps/monte-carlo/logMeanExp-scores_words.txt", sep="\t", header=TRUE)
dataExp2 = dataExp2 %>%
  left_join(
    scoresExp2 %>%
      dplyr::select(-score, -score15000),
    by = "phon"
  )

dataExp2$response = as.factor(dataExp2$response)
AICs = c()
for (vocabsize in vocabsizes) {
  # mod = clmm(
  #   as.formula(paste0("response ~ c.(score", vocabsize, ") + diacritic + (1 + c.(score", vocabsize, ") + diacritic|workerId) + (1|phon)")),
  #   data=dataExp2
  # )
  # saveRDS(mod, file=paste0("dumps/clmm/modelExp2_WordTypes_Vocab", vocabsize, ".rds"))
  mod = readRDS(paste0("dumps/clmm/modelExp2_WordTypes_Vocab", vocabsize, ".rds"))
  AICs = c(AICs, AIC(mod))
}

tibble(
  "Vocabulary size"=vocabsizes,
  "AIC" = AICs
  ) %>%
  display_table(digits=1, highlight=c("AIC"), caption="Table S5: Stage 2 comparison of AIC using phonotactic scores based on different vocabulary sizes)")

remove(mod, vocabsize, vocabsizes, AICs, scoresExp2)
```

Table S5: Stage 2 comparison of AIC using phonotactic scores based on different vocabulary sizes)

| Vocabulary size | AIC |
| --- | --- |
| 500 | 26896.4 |
| 1000 | 26883.2 |
| 1500 | 26881.9 |
| 2000 | 26881.4 |
| 2500 | 26884.4 |
| 3000 | 26884.4 |
| 3500 | 26885.8 |
| 4000 | 26885.5 |
| 4500 | 26886.9 |
| 5000 | 26885.8 |
| 6000 | 26885.6 |
| 7000 | 26883.8 |
| 8000 | 26883.4 |
| 9000 | 26882.4 |
| 10000 | 26882.0 |
| 11000 | 26882.2 |
| 12000 | 26881.9 |
| 13000 | 26881.6 |
| 14000 | 26881.6 |
| 15000 | 26880.7 |
| 20000 | 26882.1 |
| 25000 | 26883.9 |

### 3.4.3 Stage 3: Are morphs better?

In the third stage of the analysis, we ask whether participants’ phonotactic knowledge could be underpinned not by words, but by morphs that could be smaller than words, since morphs occur across words and thus should be easier to implicitly acquire from streams of speech. We compare two mixed-effects ordinal regression models, using phonotactic scores based on two different kinds of units:

- **Word types**: the training data consist of all of the word types in the lexicon (as in the result of stage one).
- **Morph types**: the training data consist of morph types derived from all of the words in the lexicon (see Detailed Materials and Methods, Section 4.2.2, for details of morphs). Scores assume that participants are attempting to parse stimuli into morphs (see Detailed Materials and Methods, Section 4.3.2).

The AIC values for the two models are given in Table S6.

The model with phonotactic scores based on morph types has lowest AIC. The summary of this model is given in Table S7.

```
# Stage 3 analysis of Exp2: evidence for knowledge of morphs

# The model based on word types is modelWordTypes from Stage 1
dataExp2$response = as.factor(dataExp2$response)

# modelMorphTypes <- clmm(
#   response ~
#     c.(scoreMorphsParsed) + diacritic +
#     (1 + c.(scoreMorphsParsed) + diacritic | workerId) + (1 | phon),
#   data=dataExp2
# )
# saveRDS(modelMorphTypes, file = "dumps/clmm/modelExp2_MorphTypes.rds")
modelMorphTypes <- readRDS("dumps/clmm/modelExp2_MorphTypes.rds")

tibble(
  "Phonotactic score basis"=c("Word types", 
                              "Morph types"),
  "AIC" = c(AIC(modelWordTypes),
            AIC(modelMorphTypes))
  ) %>%
  display_table(digits=1, highlight=c("AIC"), caption="Table S6: Stage 3 comparison of AIC using phonotactic scores based on word types and morph types (assuming participants are attempting to parse stimuli into morphs for morph-based scores)")
```

Table S6: Stage 3 comparison of AIC using phonotactic scores based on word types and morph types (assuming participants are attempting to parse stimuli into morphs for morph-based scores)

| Phonotactic score basis | AIC |
| --- | --- |
| Word types | 26902.2 |
| Morph types | 26838.6 |

```
# Stage 3 analysis of Exp2: summary of morph-based model

clm_table(modelMorphTypes, caption="Table S7: Ordinal mixed-effects model summary for well-formedness ratings, assuming morph-based phonotactic scores (and parsing of stimuli into morphs). All numeric variables in this model are centered.")
```

Table S7: Ordinal mixed-effects model summary for well-formedness ratings, assuming morph-based phonotactic scores (and parsing of stimuli into morphs). All numeric variables in this model are centered.

|  | Parameter | Estimate | Std. Error | \(z\) | \(p\) |  |
| --- | --- | --- | --- | --- | --- | --- |
| Effects | scoreMorphsParsed (centered) | 3.253 | 0.607 | 5.355 | <0.001 | \*\*\* |
|  | diacritic = TRUE | 0.921 | 0.253 | 3.636 | <0.001 | \*\*\* |
| Thresholds | 1|2 | -2.476 | 0.125 |  |  |  |
|  | 2|3 | -1.012 | 0.122 |  |  |  |
|  | 3|4 | 0.051 | 0.122 |  |  |  |
|  | 4|5 | 1.874 | 0.123 |  |  |  |

### 3.4.4 Stage 4: How many morphs do we need?

In the fourth stage of the analysis, we ask how many morph types underpin the phonotactic knowledge evidenced by participants, and whether participants attempt to parse the stimuli into morphs when they evaluate them phonotactically. As in Stage 2, our investigation has the goal of reducing the size of the proto-lexicon required to best explain participants’ phonotactic knowledge.

We conduct Monte Carlo analyses that compare fixed-effects ordinal regression models, using phonotactic scores based on different numbers of morph types. As in Stage 2, we consider two different ways of sampling a *morph set* consisting of a fixed number of morphs (see Detailed Materials and Methods, Section 5.3, for details):

- **Unweighted**: samples morph types uniformly at random, regardless of frequency.
- **Frequency-weighted**: samples morph types proportional to their frequency.

For each sample, we also consider two different morph-parsing approaches to stimuli:

- **Unparsed**: participants do not attempt to parse the nonword stimuli into morphs, instead treating them as consisting of a single morph (see Detailed Materials and Methods, Section 4.3.1, for details).
- **Parsed**: participants attempt to parse the nonword stimuli into morphs (see Detailed Materials and Methods, Section 4.3.2, for details).  
  *(Note: this is the approach assumed in Stage 3.)*

The results are visualized in Figure S11. Lowest AIC is seen in models that assume that participants are parsing stimuli into morphs, and parsing-based models have lowest AIC with the use of phonotactic scores based on a all 12,773 morphs.

```
# Stage 4 analysis of Exp2: MC evidence for size of morph-based proto-lexicon
# Note: this section of the code can be re-run, but it will take a long time (~24hrs) and create a lot of files in the directory denoted by the variable "container".
# Use of the scripts requires SRILM to be installed.
# On a Windows machine, use of the scripts additionally requires a POSIX shell (e.g. cygwin) to be installed, with the corresponding env set to the environment variable R_SHELL.

morphsetsizes = c(seq(from=500, to=4500, by=500), seq(from=5000, to=12000, by=1000))
numsamples = 1000

container <- "EXCLUDED" # Set to "." for current directory

# Set up file structure
dir.create(paste(container, "MC_morphs", sep="/"))
for (scheme in c("unweighted", "freq-weighted")) {
  dir.create(paste(container, "MC_morphs", scheme, sep="/"))
  dir.create(paste(container, "MC_morphs", scheme, "samples", sep="/"))
  for (parsing in c("parsed", "unparsed")) {
    dir.create(paste(container, "MC_morphs", scheme, parsing, sep="/"))
    for (subdir in c("auxiliaries", "phonotactic-models", "probs")) {
      dir.create(paste(container, "MC_morphs", scheme, parsing, subdir, sep="/"))
      for (morphsetsize in morphsetsizes) {
        dir.create(paste(container, "MC_morphs", scheme, "samples", morphsetsize, sep="/"))
        dir.create(paste(container, "MC_morphs", scheme, parsing, subdir, morphsetsize, sep="/"))
      }
    }
  }
}
remove(subdir)

# Step 1: get samples
morphFreq <- read.csv("spanish_morphs-and-freq.txt", sep="\t", header=TRUE, stringsAsFactors=FALSE, encoding="UTF-8") %>%
  mutate(
    morph = gsub("(.)", "\\1 ", morph) %>% trimws(.)
  )
set.seed(1234)

for (morphsetsize in morphsetsizes) {
  for (samplenum in 1:numsamples) {
    
    # Unweighted sample
    sample(morphFreq$morph, morphsetsize, replace=FALSE) %>%
      write.table(., 
                  paste(container, "MC_morphs/unweighted/samples", 
                        morphsetsize, 
                        paste0("sample_", samplenum, ".txt"), 
                        sep="/"), 
                  row.names=FALSE, col.names=FALSE, quote=FALSE)
    
    # Frequency-weighted sample
    sample(morphFreq$morph, morphsetsize, replace=FALSE, prob=morphFreq$count) %>%
      write.table(., 
                  paste(container, "MC_morphs/freq-weighted/samples", 
                        morphsetsize, 
                        paste0("sample_", samplenum, ".txt"), 
                        sep="/"), 
                  row.names=FALSE, col.names=FALSE, quote=FALSE)
  }    
}
remove(morphFreq)

# Step 2: calculate phonotactic probabilities, using the score-unparsed.sh and score-parsed.sh shell scripts
setwd("scripts")
for (scheme in c("unweighted", "freq-weighted")) {
  for (parsing in c("parsed", "unparsed")) {
    if (parsing=="parsed") {
      stimsuffix = "_parsed"
    } else {
      stimsuffix = ""
    }
    for (morphsetsize in morphsetsizes) {
      for (samplenum in 1:numsamples) {
        shellCommand <- paste0(
          "sh score-", parsing ,".sh", 
          " ../", container, "/MC_morphs/", scheme, "/samples/", morphsetsize, "/sample_", samplenum, ".txt", #" ../", container, "/MC_morphs/", scheme, "/samples/", morphsetsize, "/sample_", samplenum, ".txt",
          " stimuli", stimsuffix, ".txt",
          " ../", container, "/MC_morphs/", scheme, "/", parsing, "/phonotactic-models/", morphsetsize, #" ../", container, "/MC_morphs/", scheme, "/", parsing, "/phonotactic-models/", morphsetsize,
          " ../", container, "/MC_morphs/", scheme, "/", parsing, "/auxiliaries/", morphsetsize, #" ../", container, "/MC_morphs/", scheme, "/", parsing, "/auxiliaries/", morphsetsize,
          " ../", container, "/MC_morphs/", scheme, "/", parsing, "/probs/", morphsetsize #" ../", container, "/MC_morphs/", scheme, "/", parsing, "/probs/", morphsetsize
        )
        run_shell(shellCommand)
      }
    }
  }
}
setwd("..")
remove(shellCommand, stimsuffix)

# Step 3: compute fixed-effects ordinal regression models and save the AIC values
dataExp2$response <- as.factor(dataExp2$response)
AIC_morphs = data.frame(
  scheme = rep(c("unweighted", "freq-weighted"), each=2*length(morphsetsizes)*numsamples),
  parsing = rep(rep(c("parsed", "unparsed"), each=length(morphsetsizes)*numsamples), 2),
  morphsetsize = rep(rep(morphsetsizes, each=numsamples), 2*2),
  samplenum = rep(1:numsamples, 2*2*length(morphsetsizes)),
  aic = rep(NA, 2*2*length(morphsetsizes)*numsamples)
)
for (scheme in c("unweighted", "freq-weighted")) {
  for (parsing in c("parsed", "unparsed")) {
    for (morphsetsize in morphsetsizes) {
      aic_values = rep(NA, numsamples)
      for (samplenum in 1:numsamples) {
        mod <- clm(
          response ~ c.(scoreSample) + diacritic,
          data=add_scores(dataExp2, paste0(container, "/MC_morphs/", scheme, "/", parsing, "/probs/", morphsetsize, "/sample_", samplenum, ".csv"), "scoreSample")
        )
        aic_values[samplenum] <- AIC(mod)
      }
      AIC_morphs[AIC_morphs$scheme==scheme & AIC_morphs$parsing==parsing & AIC_morphs$morphsetsize==morphsetsize, "aic"] <- aic_values
    }
  }
}

write.table(AIC_morphs, file="dumps/monte-carlo/AIC_morphs.txt", row.names=FALSE, quote=FALSE, sep="\t")
remove(AIC_morphs, aic_values, mod, samplenum, morphsetsize, numsamples, parsing, scheme, container)
```

```
# Plot of stage 4 analysis of Exp2: MC evidence for size of morph-based proto-lexicon

figS11dat <- read.delim("dumps/monte-carlo/AIC_morphs.txt", sep="\t", header=TRUE) %>%
  group_by(scheme, parsing, morphsetsize) %>%
  summarise(
    mean.AIC = mean(aic),
    lower.AIC = quantile(aic, probs=c(0.025)),
    upper.AIC = quantile(aic, probs=c(0.975))
  ) %>%
  ungroup() %>%
  mutate(
    parsing = fct_relevel(parsing, "parsed")
  )

# The AIC values from the full lexicon for each group are preserved from earlier

# Get the AIC values for the full morph set with different parsing, in a fixed-effects setting
fullMorphParsedAIC <- clm(
  response ~ c.(scoreMorphsParsed) + diacritic,
  data=dataExp2
) %>%
  AIC(.)
fullMorphUnparsedAIC <- clm(
  response ~ c.(scoreMorphsUnparsed) + diacritic,
  data=dataExp2
) %>%
  AIC(.)

fullMorphAICs <- data.frame(
  parsing = c("parsed", "unparsed"),
  aic = c(fullMorphParsedAIC, fullMorphUnparsedAIC)
)

remove(fullMorphParsedAIC, fullMorphUnparsedAIC)

ggplot(figS11dat, aes(x=morphsetsize, y=mean.AIC, ymin=lower.AIC, ymax=upper.AIC, color=scheme, fill=scheme)) +
  geom_errorbar(position=position_dodge(width=200), size=1) +
  geom_line(position=position_dodge(width=200), size=2, alpha=0.5) +
  geom_hline(aes(yintercept=fullLexAIC, linetype="All words (93.8k)"), color="gray30", size=1) +   
  geom_hline(data=fullMorphAICs, aes(yintercept=aic, linetype="All morphs (12.8k)"), color="black", size=2) + 
  geom_point(shape=21, color="black", position=position_dodge(width=200), size=3) +
  facet_grid(. ~ parsing, labeller=labeller(parsing=function(s) paste("Stimuli:", s))) +
  labs(
    # title="Morph-based Monte Carlo analyses",
    x="Morph set size", 
    y="AIC score"
  ) + 
  scale_x_continuous(
    breaks=seq(from=2000, to=12000, by=2000),
    labels=function(b){return(paste0(b/1000,"k"))}
    ) +
  scale_color_manual(
    name="Sampling", 
    breaks=c("unweighted", "freq-weighted"), 
    values=c("dodgerblue3", "firebrick3"),
    labels=c("Unweighted", "Frequency-weighted")
  ) +
  scale_fill_manual(
    name="Sampling", 
    breaks=c("unweighted", "freq-weighted"), 
    values=c("dodgerblue3", "firebrick3"),
    labels=c("Unweighted", "Frequency-weighted")
  ) +
  scale_linetype_manual(name=NULL, values=c("All words (93.8k)"="dashed", "All morphs (12.8k)"="dotted")) +
  guides(color=guide_legend(order=1), fill=guide_legend(order=1), linetype=guide_legend(order=2)) +
  theme_bw() + 
  theme(
    plot.title=element_text(hjust=0.5, size=18),
    axis.title = element_text(size=16, color="black"),
    axis.text = element_text(size=14, color="black"),
    legend.title = element_text(size=16, color="black"),
    legend.text = element_text(size=15, color="black"),
    strip.text = element_text(size=15, color="black")
  )

remove(figS11dat, fullLexAIC, fullMorphAICs)
```

Figure S11: Monte Carlo analyses with 1,000 samples over 7 sizes of morph set, with different assumptions about the parsing of stimuli into morphs. The bars represent 95% bootstrap percentile intervals.

As in Stage 3, we also consider comparisons of mixed-effects models that average 1,000 phonotactic score systems, based on proto-lexicons of the same size formed from different frequency-weighted samplings of the lexicon. We compare results for all of the morph set sizes explored in the Monte Carlo analysis. We used phonotactic score systems that assume that participants are attempting to parse stimuli into morphs, since the models with parsing-based scores performed better than those without in the Monte Carlo analysis.

The results of the mixed-effects model comparison are presented in Table S8. The lowest AIC comes from the use of averaged phonotactic scores based on a proto-lexicon of 12,000 morphs, under the assumption that participants are attempting to parse stimuli into morphs. These scores are preserved in the original dataset as `scoreMorphParsed12000`.

```
# Stage 4 analysis of Exp2 continued: MC evidence for size of morph-based proto-lexicon

morphsetsizes = c(seq(from=500, to=4500, by=500), seq(from=5000, to=12000, by=1000))

# container <- "EXCLUDED" # Set to "." for current directory
# 
# scoresExp2 = itemsExp2 %>%
#   dplyr::select(phon, scoreMorphsParsed) %>%
#   distinct()
# 
# for (morphsetsize in morphsetsizes) {
#   path = paste0(container, "/MC_morphs/freq-weighted/parsed/probs/", morphsetsize, "/")
#   scoreName = paste0("scoreMorphsParsed", morphsetsize)
#   scoresExp2 = scoresExp2 %>%
#     left_join(
#       logMeanExp_scores(path, 1000) %>%
#         rename(!!scoreName := scoreAverage),
#       by = "phon"
#     )
# }
# 
# write.table(scoresExp2, file="dumps/monte-carlo/logMeanExp-scores_morphs.txt", row.names=FALSE, quote=FALSE, sep="\t")
# remove(scoresExp2, container, morphsetsize, path, scoreName)

scoresExp2 = read.delim("dumps/monte-carlo/logMeanExp-scores_morphs.txt", sep="\t", header=TRUE)
dataExp2 = dataExp2 %>%
  left_join(
    scoresExp2 %>%
      dplyr::select(-scoreMorphsParsed, -scoreMorphsParsed12000),
    by = "phon"
  )

dataExp2$response = as.factor(dataExp2$response)
AICs = c()
for (morphsetsize in morphsetsizes) {
  # mod = clmm(
  #   as.formula(paste0("response ~ c.(scoreMorphsParsed", morphsetsize, ") + diacritic + (1 + c.(scoreMorphsParsed", morphsetsize, ") + diacritic|workerId) + (1|phon)")),
  #   data=dataExp2, control=list(maxIter=100)
  # )
  # saveRDS(mod, file=paste0("dumps/clmm/modelExp2_MorphTypes_Morphset", morphsetsize, ".rds"))
  mod = readRDS(paste0("dumps/clmm/modelExp2_MorphTypes_Morphset", morphsetsize, ".rds"))
  AICs = c(AICs, AIC(mod))
}

tibble(
  "Morphset size"=morphsetsizes,
  "AIC" = AICs
  ) %>%
  display_table(digits=1, highlight=c("AIC"), caption="Table S8: Stage 4 comparison of AIC using phonotactic scores based on different morphset sizes)")

remove(mod, morphsetsize, morphsetsizes, AICs, scoresExp2)
```

Table S8: Stage 4 comparison of AIC using phonotactic scores based on different morphset sizes)

| Morphset size | AIC |
| --- | --- |
| 500 | 26884.5 |
| 1000 | 26871.3 |
| 1500 | 26871.9 |
| 2000 | 26872.3 |
| 2500 | 26872.7 |
| 3000 | 26871.1 |
| 3500 | 26867.7 |
| 4000 | 26864.0 |
| 4500 | 26860.9 |
| 5000 | 26859.2 |
| 6000 | 26854.6 |
| 7000 | 26851.8 |
| 8000 | 26849.9 |
| 9000 | 26847.6 |
| 10000 | 26844.4 |
| 11000 | 26840.6 |
| 12000 | 26839.2 |

### 3.4.5 Stage 5: What is the best source of phonotactic knowledge?

In the fifth and final stage of the analysis, we compare the final models from previous stages, to identify once and for all the best phonotactic scores for predicting participants’ ratings.

We compare mixed-effects ordinal regression models that use four different phonotactic scores:

- **Word types**: phonotactic scores are based on all 93,777 word types in the lexicon, as in stage one.
- **Best average over subsets of word types**: phonotactic scores are averaged over 1,000 different phonotactic score systems, based on sampling 15,000 word types in proportion to frequency, as in stage two.
- **Morph types**: phonotactic scores are based on the 12,773 morph types derived from all words in the lexicon, and are calculated under the assumption that participants parse stimuli into morphs, as in stage three.
- **Best average over subsets of morph types**: phonotactic scores are averaged over 1,000 different phonotactic score systems, based on sampling 12,000 morph types in proportion to frequency, and are calculated under the assumption that participants parse stimuli into morphs, as in stage four.

The results of the comparison are presented in Table S9. The lowest AIC comes from the use of phonotactic scores based on all morph types. A summary of the best-fitting model is presented in Table S10.

```
# Stage 5 analysis of Exp2: comparison of candidate models

# modelWordTypes <- clmm(
#   response ~
#     c.(score) + diacritic +
#     (1 + c.(score) + diacritic | workerId) + (1 | phon),
#   data=dataExp2
# )
modelWordTypes <- readRDS("dumps/clmm/modelExp2_WordTypes.rds")

# The model based on the best averaged subset of word types is based on 1000 word types

# modelSubsetWordTypes <- clmm(
#   response ~ 
#     c.(score15000) + diacritic + 
#     (1 + c.(score15000) + diacritic | workerId) + (1 | phon), 
#   data=dataExp2
# )
modelSubsetWordTypes <- readRDS("dumps/clmm/modelExp2_WordTypes_Vocab15000.rds")

# modelMorphTypes <- clmm(
#   response ~ 
#     c.(scoreMorphsParsed) + diacritic + 
#     (1 + c.(scoreMorphsParsed) + diacritic | workerId) + (1 | phon), 
#   data=dataExp2
# )
modelMorphTypes <- readRDS("dumps/clmm/modelExp2_MorphTypes.rds")

# modelSubsetMorphTypes <- clmm(
#   response ~ 
#     c.(scoreMorphsParsed12000) + diacritic + 
#     (1 + c.(scoreMorphsParsed12000) + diacritic | workerId) + (1 | phon), 
#   data=dataExp2
# )
modelSubsetMorphTypes <- readRDS("dumps/clmm/modelExp2_MorphTypes_Morphset12000.rds")

tibble(
  "Source of phonotactics"=c("All word types (93.8k)",
                             "Best average over subsets of word types (15k)",
                             "All morph types (12.8k)",
                             "Best average over subsets of morph types (12k)"),
  "AIC" = c(AIC(modelWordTypes),
            AIC(modelSubsetWordTypes),
            AIC(modelMorphTypes),
            AIC(modelSubsetMorphTypes))
  ) %>%
  display_table(digits=1, highlight=c("AIC"), caption="Table S9: Stage 5 comparison of AIC using best-performing phonotactic scores from all stages.")

remove(modelWordTypes, modelSubsetWordTypes, modelSubsetMorphTypes)
```

Table S9: Stage 5 comparison of AIC using best-performing phonotactic scores from all stages.

| Source of phonotactics | AIC |
| --- | --- |
| All word types (93.8k) | 26902.2 |
| Best average over subsets of word types (15k) | 26880.7 |
| All morph types (12.8k) | 26838.6 |
| Best average over subsets of morph types (12k) | 26839.2 |

```
# Stage 5 analysis of Exp2: summary of best model
clm_table(modelMorphTypes, caption="Table S10: Summary of best-fitting ordinal mixed-effects model of well-formedness ratings. The model assumes morph-based phonotactic scores and parsing of stimuli into morphs, with a proto-lexicon consisting of all 12,773 morphs. All numeric variables in this model are centered.")

remove(modelMorphTypes)
```

Table S10: Summary of best-fitting ordinal mixed-effects model of well-formedness ratings. The model assumes morph-based phonotactic scores and parsing of stimuli into morphs, with a proto-lexicon consisting of all 12,773 morphs. All numeric variables in this model are centered.

|  | Parameter | Estimate | Std. Error | \(z\) | \(p\) |  |
| --- | --- | --- | --- | --- | --- | --- |
| Effects | scoreMorphsParsed (centered) | 3.253 | 0.607 | 5.355 | <0.001 | \*\*\* |
|  | diacritic = TRUE | 0.921 | 0.253 | 3.636 | <0.001 | \*\*\* |
| Thresholds | 1|2 | -2.476 | 0.125 |  |  |  |
|  | 2|3 | -1.012 | 0.122 |  |  |  |
|  | 3|4 | 0.051 | 0.122 |  |  |  |
|  | 4|5 | 1.874 | 0.123 |  |  |  |

## 3.5 Effects of attitudes and exposure

Having determined the basic structure of the results, we now explore the role that attitudes and exposure to Spanish play in these results.

In these analyses, we use the best phonotactic score metric established earlier, `scoreMorphsParsed`. The distribution of phonotactic scores according to this metric has two outliers with scores below -1.3; all other stimuli have scores larger than or equal to -1.16. In order to prevent these stimuli exerting undue leverage on the results, we exclude them from these analyses.

### 3.5.1 Visualization of raw data

Across the three predictors of interest – Spanish value, nationalism, and exposure to Spanish – there are two patterns that stand out as particularly strong and consistent in the assignment of wellformedness ratings. First, the more value a participant places on Spanish and its speakers in their home state, the more sensitive they seem to be to phonotactic score. Second, the more exposure a participant has to Spanish, the less affected they seem to be by the presence of non-English characters.

```
# Plot of raw data for Exp2, by phonotactic score, presence of non-English characters, and attitude/exposure predictors

valueplot_sep = dataExp2 %>%
  filter(scoreMorphsParsed >= -1.16) %>%
  mutate(
    value = factor(
      case_when(
        proSpanish < 0 ~ "value: negative",
        proSpanish > 0 ~ "value: positive",
        proSpanish == 0 ~ "value: neutral"
      ),
      levels = c("value: positive", "value: neutral", "value: negative")
    )
  ) %>%
  group_by(value, phon, diacritic, scoreMorphsParsed) %>%
  summarise(
    mean_response = mean(as.numeric(response)),
    n = n()
  ) %>%
  ungroup() %>%
  mutate(
    diacritic = factor(diacritic, levels = c(FALSE, TRUE), labels = c("Non-English chars: absent", "Non-English chars: present"))
  ) %>%
  ggplot(., aes(x=scoreMorphsParsed, y=mean_response, color=value, fill=value)) +
    geom_point(shape=1, alpha=0.3, size=4) +
    geom_smooth(method="lm", formula="y~x", alpha=0.6, size=2) +
    facet_grid(value ~ diacritic, switch="y") +
    labs(y = "Mean Rating (by item)", x = "Phonotactic score") +
    scale_fill_manual(name = "Spanish value",
                      values = c("forestgreen", "goldenrod3", "firebrick3"),
                      guide = "none") +  
    scale_color_manual(name = "Spanish value",
                       values = c("forestgreen", "goldenrod3", "firebrick3"),
                       guide = "none") +
    theme_bw() +
    ggtitle("Spanish value") +
    ylim(1, 5) +
    theme(
      panel.grid = element_blank(),
      axis.title = element_text(size=12, color="black"),
      axis.text = element_text(size=10, color="black"),
      legend.title = element_text(size=12, color="black"),
      legend.text = element_text(size=11, color="black"),
      strip.text = element_text(size=11, color="black"),
      plot.title = element_text(size=14, color="black", hjust=0.5)
    )

nationalismplot_sep = dataExp2 %>%
  filter(scoreMorphsParsed >= -1.16) %>%
  mutate(
    nationalism = factor(
      case_when(
        nationalism < 0 ~ "nationalism: low",
        nationalism > 0 ~ "nationalism: mid",
        nationalism == 0 ~ "nationalism: high"
      ),
      levels = c("nationalism: high", "nationalism: mid", "nationalism: low")
    )
  ) %>%
  group_by(nationalism, phon, diacritic, scoreMorphsParsed) %>%
  summarise(
    mean_response = mean(as.numeric(response)),
    n = n()
  ) %>%
  ungroup() %>%
  mutate(
    diacritic = factor(diacritic, levels = c(FALSE, TRUE), labels = c("Non-English chars: absent", "Non-English chars: present"))
  ) %>%
  ggplot(., aes(x=scoreMorphsParsed, y=mean_response, color=nationalism, fill=nationalism)) +
    geom_point(shape=1, alpha=0.3, size=4) +
    geom_smooth(method="lm", formula="y~x", alpha=0.6, size=2) +
    facet_grid(nationalism ~ diacritic, switch="y") +
    labs(y = "Mean Rating (by item)", x = "Phonotactic score") +
    scale_fill_manual(name = "Nationalism",
                      values = c("firebrick3", "goldenrod3", "forestgreen"),
                      guide = "none") +  
    scale_color_manual(name = "Nationalism",
                       values = c("firebrick3", "goldenrod3", "forestgreen"),
                       guide = "none") +
    theme_bw() +
    ggtitle("Nationalism") +
    ylim(1, 5) +
    theme(
      panel.grid = element_blank(),
      axis.title = element_text(size=12, color="black"),
      axis.text = element_text(size=10, color="black"),
      legend.title = element_text(size=12, color="black"),
      legend.text = element_text(size=11, color="black"),
      strip.text = element_text(size=11, color="black"),
      plot.title = element_text(size=14, color="black", hjust=0.5)
    )

expoplot_sep = dataExp2 %>%
  filter(scoreMorphsParsed >= -1.16) %>%
  mutate(
    exposure = factor(
      case_when(
        spanishExpo == 2 ~ "exposure: low",
        spanishExpo <= 4 ~ "exposure: mid",
        spanishExpo > 4 ~ "exposure: high"
      ),
      levels = c("exposure: high", "exposure: mid", "exposure: low")
    )
  ) %>%
  group_by(exposure, phon, diacritic, scoreMorphsParsed) %>%
  summarise(
    mean_response = mean(as.numeric(response)),
    n = n()
  ) %>%
  ungroup() %>%
  mutate(
    diacritic = factor(diacritic, levels = c(FALSE, TRUE), labels = c("Non-English chars: absent", "Non-English chars: present"))
  ) %>%
  ggplot(., aes(x=scoreMorphsParsed, y=mean_response, color=exposure, fill=exposure)) +
    geom_point(shape=1, alpha=0.3, size=4) +
    geom_smooth(method="lm", formula="y~x", alpha=0.6, size=2) +
    facet_grid(exposure ~ diacritic, switch="y") +
    labs(y = "Mean Rating (by item)", x = "Phonotactic score") +
    scale_fill_manual(name = "Spanish exposure",
                      values = c("forestgreen", "goldenrod3", "firebrick3"),
                      guide = "none") +  
    scale_color_manual(name = "Spanish exposure",
                       values = c("forestgreen", "goldenrod3", "firebrick3"),
                       guide = "none") +
    theme_bw() +
    ggtitle("Spanish exposure") +
    ylim(1, 5) +
    theme(
      panel.grid = element_blank(),
      axis.title = element_text(size=12, color="black"),
      axis.text = element_text(size=10, color="black"),
      legend.title = element_text(size=12, color="black"),
      legend.text = element_text(size=11, color="black"),
      strip.text = element_text(size=11, color="black"),
      plot.title = element_text(size=14, color="black", hjust=0.5)
    )

valueplot_joint = dataExp2 %>%
  filter(scoreMorphsParsed >= -1.16) %>%
  mutate(
    value = factor(
      case_when(
        proSpanish < 0 ~ "negative",
        proSpanish > 0 ~ "positive",
        proSpanish == 0 ~ "neutral"
      ),
      levels = c("positive", "neutral", "negative")
    )
  ) %>%
  group_by(value, phon, diacritic, scoreMorphsParsed) %>%
  summarise(
    mean_response = mean(as.numeric(response)),
    n = n()
  ) %>%
  ungroup() %>%
  mutate(
    diacritic = factor(diacritic, levels = c(FALSE, TRUE), labels = c("Non-English chars: absent", "Non-English chars: present"))
  ) %>%
  ggplot(., aes(x=scoreMorphsParsed, y=mean_response, color=value, fill=value)) +
    geom_smooth(method="lm", formula="y~x", alpha=0.15, size=2) +
    facet_grid(. ~ diacritic, switch="y") +
    labs(y = "Mean Rating (by item)", x = "Phonotactic score") +
    scale_fill_manual(name = "Spanish value   ",
                      labels = c("positive   ", "neutral   ", "negative"),
                      values = c("forestgreen", "goldenrod3", "firebrick3")) +  
    scale_color_manual(name = "Spanish value   ",
                       labels = c("positive   ", "neutral   ", "negative"),
                       values = c("forestgreen", "goldenrod3", "firebrick3")) +
    theme_bw() +
    ylim(1, 5) +
    theme(
      panel.grid = element_blank(),
      axis.title = element_text(size=12, color="black"),
      axis.text = element_text(size=10, color="black"),
      legend.title = element_text(size=12, color="black"),
      legend.text = element_text(size=11, color="black"),
      strip.text = element_text(size=11, color="black"),
      legend.position = "bottom"
    )

nationalismplot_joint = dataExp2 %>%
  filter(scoreMorphsParsed >= -1.16) %>%
  mutate(
    nationalism = factor(
      case_when(
        nationalism < 0 ~ "low",
        nationalism > 0 ~ "mid",
        nationalism == 0 ~ "high"
      ),
      levels = c("high", "mid", "low")
    )
  ) %>%
  group_by(nationalism, phon, diacritic, scoreMorphsParsed) %>%
  summarise(
    mean_response = mean(as.numeric(response)),
    n = n()
  ) %>%
  ungroup() %>%
  mutate(
    diacritic = factor(diacritic, levels = c(FALSE, TRUE), labels = c("Non-English chars: absent", "Non-English chars: present"))
  ) %>%
  ggplot(., aes(x=scoreMorphsParsed, y=mean_response, color=nationalism, fill=nationalism)) +
    geom_smooth(method="lm", formula="y~x", alpha=0.15, size=2) +
    facet_grid(. ~ diacritic, switch="y") +
    labs(y = "Mean Rating (by item)", x = "Phonotactic score") +
    scale_fill_manual(name = "Nationalism   ",
                      labels = c("high   ", "mid   ", "low"),
                      values = c("firebrick3", "goldenrod3", "forestgreen")) +  
    scale_color_manual(name = "Nationalism   ",
                       labels = c("high   ", "mid   ", "low"),
                       values = c("firebrick3", "goldenrod3", "forestgreen")) +
    theme_bw() +
    ylim(1, 5) +
    theme(
      panel.grid = element_blank(),
      axis.title = element_text(size=12, color="black"),
      axis.text = element_text(size=10, color="black"),
      legend.title = element_text(size=12, color="black"),
      legend.text = element_text(size=11, color="black"),
      strip.text = element_text(size=11, color="black"),
      plot.title = element_text(size=14, color="black", hjust=0.5),
      legend.position = "bottom"
    )

expoplot_joint = dataExp2 %>%
  filter(scoreMorphsParsed >= -1.16) %>%
  mutate(
    exposure = factor(
      case_when(
        spanishExpo == 2 ~ "low",
        spanishExpo <= 4 ~ "mid",
        spanishExpo > 4 ~ "high"
      ),
      levels = c("high", "mid", "low")
    )
  ) %>%
  group_by(exposure, phon, diacritic, scoreMorphsParsed) %>%
  summarise(
    mean_response = mean(as.numeric(response)),
    n = n()
  ) %>%
  ungroup() %>%
  mutate(
    diacritic = factor(diacritic, levels = c(FALSE, TRUE), labels = c("Non-English chars: absent", "Non-English chars: present"))
  ) %>%
  ggplot(., aes(x=scoreMorphsParsed, y=mean_response, color=exposure, fill=exposure)) +
    geom_smooth(method="lm", formula="y~x", alpha=0.15, size=2) +
    facet_grid(. ~ diacritic, switch="y") +
    labs(y = "Mean Rating (by item)", x = "Phonotactic score") +
    scale_fill_manual(name = "Spanish exposure   ",
                      labels = c("high   ", "mid   ", "low"),
                      values = c("forestgreen", "goldenrod3", "firebrick3")) +
    scale_color_manual(name = "Spanish exposure   ",
                       labels = c("high   ", "mid   ", "low"),
                       values = c("forestgreen", "goldenrod3", "firebrick3")) +
    theme_bw() +
    ylim(1, 5) +
    theme(
      panel.grid = element_blank(),
      axis.title = element_text(size=12, color="black"),
      axis.text = element_text(size=10, color="black"),
      legend.title = element_text(size=12, color="black"),
      legend.text = element_text(size=11, color="black"),
      strip.text = element_text(size=11, color="black"),
      plot.title = element_text(size=14, color="black", hjust=0.5),
      legend.position = "bottom"
    )

ggarrange(valueplot_sep, nationalismplot_sep, expoplot_sep, valueplot_joint, nationalismplot_joint, expoplot_joint, ncol=3, labels=c("a.", "b.", "c.", "", "", ""), heights=c(3,1))
remove(valueplot_sep, nationalismplot_sep, expoplot_sep, valueplot_joint, nationalismplot_joint, expoplot_joint)


# Figure from the paper
# dataExp2 %>%
#   filter(scoreMorphsParsed >= -1.16) %>%
#   mutate(
#     value = factor(
#       case_when(
#         proSpanish <= 0 ~ "neutr./neg.",
#         proSpanish > 0 ~ "pos."
#       ),
#       levels = c("pos.", "neutr./neg."),
#       labels = c("Positive", "Neutral / negative")
#     )
#   ) %>%
#   group_by(value, phon, diacritic, scoreMorphsParsed) %>%
#   summarise(
#     mean_response = mean(as.numeric(response)),
#     n = n()
#   ) %>%
#   ungroup() %>%
#   mutate(
#     diacritic = factor(diacritic, levels = c(FALSE, TRUE), labels = c("Non-English chars: absent", "Non-English chars: present"))
#   ) %>%
#   ggplot(., aes(x=scoreMorphsParsed, y=mean_response, color=value, fill=value)) +
#     geom_point(shape=1, alpha=0.2, size=3) +
#     geom_smooth(method="lm", formula="y~x", alpha=0.6, size=2) +
#     labs(y = "Mean Rating (by item)", x = "Phonotactic score") +
#     scale_fill_manual(name = "Spanish value",
#                       values = c("forestgreen", "firebrick3")) +
#     scale_color_manual(name = "Spanish value",
#                        values = c("forestgreen", "firebrick3")) +
#     theme_bw() +
#     ylim(1, 5) +
#     theme(
#       panel.grid = element_blank(),
#       axis.title = element_text(size=16, color="black"),
#       axis.text = element_text(size=14, color="black"),
#       legend.text = element_text(size=15, color="black"),
#       legend.title = element_text(size=16, color="black"),
#       strip.text.x = element_text(size=15, color="black"),
#       strip.text.y = element_text(size=15, color="white", face="bold")
#     )
```

Figure S12: Mean phonotactic well-formedness ratings for each nonword stimulus, as affected by attitudes and exposure. Figure S12a partitions participants by Spanish value, Figure S12b partitions participants by nationalism, and Figure S12c partitions participants by exposure to Spanish. In each subplot, the lefthand facets represent responses to nonwords that lack any non-English characters, and the righthand facets represent responses to nonwords that have at least one non-English character. In the top panel of each subplot, participants that are partitioned into different groups are represented in different facets, arranged vertically, and points are used to represent mean ratings for each unique nonword. In the bottom panel of each subplot, trends are represented for all participant groups in the same facet, to aid comparison and identification of patterns.

### 3.5.2 Statistical analysis

As in Experiment 1, we add predictors for attitude toward Spanish (`proSpanish`), nationalism (`nationalism`), and exposure to Spanish (`spanishExpo`) to the final model obtained from the previous analysis. We allow each of these predictors to interact with all of the already-established terms, but not with each other, for reasons of limited participant numbers.

We then follow the stepwise procedure described previously. The final model is given in Table S11, and partial effects plots are given in Figure S13.

Interactions between attitude and phonotactic score are not justified to be retained in the final model, according to our model selection criteria. However, the fit of the final model is ill defined, so we report the penultimate model, which retains an interaction between `proSpanish` and phonotactic score was the last term to be removed from the model. This interaction shows numerical patterns (also evidenced in the raw data) whereby participants who value Spanish more (i.e. with more positive attitudes) appeared to be more sensitive to phonotactic scores in determining their wellformedness ratings than those who valued it less (i.e. with more negative attitudes). Especially in light of the statistically significant equivalent effect in the Word Identification task, this is an intriguing pattern that future work should follow up on.

We see significant interactions between the presence of non-English characters and each of the predictors of interest. Since the presence of non-English characters is treated as a control predictor, its interactions do not bear on our central research question; furthermore, since the subset of stimuli containing non-English characters is relatively small, it is likely that robust estimation of these interactions would require data from more participants than we currently have, especially given a maximal random effect structure. For these reasons, we do not interpret these results, but simply state them as follows: The more value a participant places on Spanish and/or the more exposure they have to Spanish, the less likely they are to be affected by the presence of a non-English character in their determination of a wellformedness rating. A similar pattern with very different implications is seen for nationalism: the more nationalist attitudes a participant expresses, the less likely they are to be affected by the presence of a non-English character.

```
# Statistical analysis of attitude/exposure in Exp2 wellformedness ratings

dataExp2$response <- as.factor(dataExp2$response)

# # Model fitting process: fixed-effects only
# m1 <- clm(response ~ (c.(scoreMorphsParsed) + diacritic) * (c.(proSpanish) + c.(nationalism) + c.(spanishExpo)), data=filter(dataExp2, scoreMorphsParsed >= -1.16))
# anova(m1, type="III") # Removal candidate: c.(scoreMorphsParsed):c.(spanishExpo)
# m2 <- update(m1, . ~ . - c.(scoreMorphsParsed):c.(spanishExpo))
# anova(m2, type="III") # Removal candidate: c.(scoreMorphsParsed):c.(nationalism)
# m3 <- update(m2, . ~ . - c.(scoreMorphsParsed):c.(nationalism))
# anova(m3, type="III") # No further removal candidates
# 
# # Switch to mixed-effects
# 
# m4 <- clmm(response ~ c.(scoreMorphsParsed) * c.(proSpanish) + diacritic * (c.(proSpanish) + c.(nationalism) + c.(spanishExpo)) + (1 + c.(scoreMorphsParsed) + diacritic|workerId) + (1 + proSpanish + nationalism + spanishExpo|phon), data=filter(dataExp2, scoreMorphsParsed >= -1.16))
# summary(m4) # Removal candidate: c.(scoreMorphsParsed):c.(proSpanish)
# 
# m5 <- clmm(response ~ c.(scoreMorphsParsed) + c.(proSpanish) + diacritic * (c.(proSpanish) + c.(nationalism) + c.(spanishExpo)) + (1 + c.(scoreMorphsParsed) + diacritic|workerId) + (1 + proSpanish + nationalism + spanishExpo|phon), data=filter(dataExp2, scoreMorphsParsed >= -1.16))
# anova(m4, m5) # LR test not significant (p=0.1216), so removal is justified. But m4 has (slightly) lower AIC, so save it to facilitate exploration.
# 
# saveRDS(m4, file="dumps/clmm/modelExp2_Attitudes_withScoreInteraction.rds")
# saveRDS(m5, file="dumps/clmm/modelExp2_Attitudes.rds")
# remove(m1, m2, m3, m4, m5)

# The variance-covariance matrix of the parameters of m5 is not defined, so we use m4 instead
modelExp2Attitudes <- readRDS("dumps/clmm/modelExp2_Attitudes_withScoreInteraction.rds")

clm_table(modelExp2Attitudes, digits=3, caption="Table S11: Ordinal mixed-effects model of wellformedness ratings, taking into account attitudes and exposure. All numeric predictors are centered.")
```

Table S11: Ordinal mixed-effects model of wellformedness ratings, taking into account attitudes and exposure. All numeric predictors are centered.

|  | Parameter | Estimate | Std. Error | \(z\) | \(p\) |  |
| --- | --- | --- | --- | --- | --- | --- |
| Effects | scoreMorphsParsed (centered) | 3.382 | 0.599 | 5.643 | <0.001 | \*\*\* |
|  | proSpanish (centered) | 0.048 | 0.064 | 0.760 | 0.447 |  |
|  | diacritic = TRUE | 0.945 | 0.209 | 4.528 | <0.001 | \*\*\* |
|  | nationalism (centered) | 0.070 | 0.052 | 1.337 | 0.181 |  |
|  | spanishExpo (centered) | -0.048 | 0.085 | -0.566 | 0.571 |  |
|  | scoreMorphsParsed (centered) × proSpanish (centered) | 0.434 | 0.276 | 1.570 | 0.116 |  |
|  | proSpanish (centered) × diacritic = TRUE | -0.267 | 0.111 | -2.415 | 0.016 | \* |
|  | diacritic = TRUE × nationalism (centered) | -0.192 | 0.087 | -2.217 | 0.027 | \* |
|  | diacritic = TRUE × spanishExpo (centered) | -0.298 | 0.137 | -2.182 | 0.029 | \* |
| Thresholds | 1|2 | -2.483 | 0.123 |  |  |  |
|  | 2|3 | -1.006 | 0.119 |  |  |  |
|  | 3|4 | 0.064 | 0.119 |  |  |  |
|  | 4|5 | 1.901 | 0.121 |  |  |  |

```
# Plot of attitude/exposure effects in Exp2 wellformedness ratings

figS13a.mean <- clm_plotdat(modelExp2Attitudes, c("scoreMorphsParsed", "proSpanish"), xlevels=list(scoreMorphsParsed=25, proSpanish=c(-4, 4)), type="mean") %>%
  mutate(
    value = factor(proSpanish, levels=c(4, -4), labels=c("V. pos.", "V. neg."))
  ) %>%
  ggplot(., aes(x=scoreMorphsParsed, y=pred, color=value, fill=value)) +
    geom_ribbon(aes(ymin=lci, ymax=uci), alpha=0.3, color=NA) +
    geom_line(size=1) +
    xlab("Phonotactic score") +
    ylab("Predicted mean rating") + 
    scale_color_manual(
      name = "Spanish\nvalue",
      breaks = c("V. pos.", "V. neg."),
      values = c("forestgreen", "firebrick3")
      ) +
    scale_fill_manual(
      name = "Spanish\nvalue",
      breaks = c("V. pos.", "V. neg."),
      values = c("forestgreen", "firebrick3")
    ) +
    scale_x_continuous(n.breaks=4) +
    ylim(1.8, 4.8) +
    theme_bw() + 
    theme(
      legend.position="none"
    )

figS13a.dist <- clm_plotdat(modelExp2Attitudes, c("scoreMorphsParsed", "proSpanish"), xlevels=list(scoreMorphsParsed=25, proSpanish=c(-4, 4)), type="dist") %>%
  mutate(
    value = factor(proSpanish, levels=c(4, -4), labels=c("V. pos.", "V. neg."))
  ) %>%
  ggplot(., aes(x=scoreMorphsParsed, y=pred, color=value, fill=value)) +
    geom_ribbon(aes(ymin=lci, ymax=uci), alpha=0.3, color=NA) +
    geom_line(size=1) +
    xlab("Phonotactic score") +
    ylab("Probability of rating") + 
    scale_x_continuous(n.breaks=4) +
    scale_color_manual(
      name = "Spanish\nvalue",
      breaks = c("V. pos.", "V. neg."),
      values = c("forestgreen", "firebrick3")
      ) +
    scale_fill_manual(
      name = "Spanish\nvalue",
      breaks = c("V. pos.", "V. neg."),
      values = c("forestgreen", "firebrick3")
    ) +
    facet_grid(. ~ response, labeller=as_labeller(function(x) str_c("Rating: ", x))) +
    ylim(0, 1) +  
    theme_bw() + 
    theme(
      legend.position="right"
    )

figS13b.mean <- clm_plotdat(modelExp2Attitudes, c("proSpanish", "diacritic"), xlevels=list(proSpanish=c(-4,4)), type="mean") %>%
  mutate(
    value = factor(proSpanish, levels=c(4, -4), labels=c("V. pos.", "V. neg.")),
    diacritic = factor(diacritic, levels=c(TRUE, FALSE), labels=c("Present", "Absent"))
  ) %>%
  ggplot(., aes(x=diacritic, y=pred, color=value, shape=value)) +
    geom_point(size=4, position=position_dodge(width=0.2)) +
    geom_line(aes(x=as.integer(diacritic)), size=1, alpha=0.4, position=position_dodge(width=0.2)) +
    geom_errorbar(aes(ymin=lci, ymax=uci), size=1, width=0.3, position=position_dodge(width=0.2)) +
    xlab("Non-English characters") +
    ylab("Predicted mean rating") + 
    scale_color_manual(
      name = "Spanish\nvalue",
      breaks = c("V. pos.", "V. neg."),
      values = c("forestgreen", "firebrick3")
      ) +
    scale_fill_manual(
      name = "Spanish\nvalue",
      breaks = c("V. pos.", "V. neg."),
      values = c("forestgreen", "firebrick3")
    ) +
    scale_shape_discrete(name = "Spanish\nvalue") +
    ylim(1.8, 4.8) +
    theme_bw() + 
    theme(
      legend.position="none"
    )

figS13b.dist <- clm_plotdat(modelExp2Attitudes, c("proSpanish", "diacritic"), xlevels=list(proSpanish=c(-4,4)), type="dist") %>%
  mutate(
    value = factor(proSpanish, levels=c(4, -4), labels=c("V. pos.", "V. neg.")),
    diacritic = factor(diacritic, levels=c(TRUE, FALSE), labels=c("Present", "Absent"))
  ) %>%
  ggplot(., aes(x=diacritic, y=pred, color=value, shape=value)) +
    geom_point(size=2, position=position_dodge(width=0.2)) +
    geom_line(aes(x=as.integer(diacritic)), size=1, alpha=0.7, position=position_dodge(width=0.2)) +
    geom_errorbar(aes(ymin=lci, ymax=uci), size=1, width=0.3, position=position_dodge(width=0.2)) +
    xlab("Non-English characers") +
    ylab("Probability of rating") + 
    scale_color_manual(
      name = "Spanish\nvalue",
      breaks = c("V. pos.", "V. neg."),
      values = c("forestgreen", "firebrick3")
      ) +
    scale_fill_manual(
      name = "Spanish\nvalue",
      breaks = c("V. pos.", "V. neg."),
      values = c("forestgreen", "firebrick3")
    ) +
    scale_shape_discrete(name = "Spanish\nvalue") +
    facet_grid(. ~ response, labeller=as_labeller(function(x) str_c("Rating: ", x))) +
    ylim(0, 1) +  
    theme_bw() + 
    theme(
      legend.position="right"
    )

figS13c.mean <- clm_plotdat(modelExp2Attitudes, c("nationalism", "diacritic"), xlevels=list(nationalism=c(-4,4)), type="mean") %>%
  mutate(
    nationalism = factor(nationalism, levels=c(4, -4), labels=c("V. high", "V. low")),
    diacritic = factor(diacritic, levels=c(TRUE, FALSE), labels=c("Present", "Absent"))
  ) %>%
  ggplot(., aes(x=diacritic, y=pred, color=nationalism, shape=nationalism)) +
    geom_point(size=4, position=position_dodge(width=0.2)) +
    geom_line(aes(x=as.integer(diacritic)), size=1, alpha=0.4, position=position_dodge(width=0.2)) +
    geom_errorbar(aes(ymin=lci, ymax=uci), size=1, width=0.3, position=position_dodge(width=0.2)) +
    xlab("Non-English characters") +
    ylab("Predicted mean rating") + 
    scale_color_manual(
      name = "Nationalism",
      breaks = c("V. high", "V. low"),
      values = c("firebrick3", "forestgreen")
      ) +
    scale_fill_manual(
      name = "Nationalism",
      breaks = c("V. high", "V. low"),
      values = c("firebrick3", "forestgreen")
    ) +
    scale_shape_discrete(name = "Nationalism") +
    ylim(1.8, 4.8) +
    theme_bw() + 
    theme(
      legend.position="none"
    )

figS13c.dist <- clm_plotdat(modelExp2Attitudes, c("nationalism", "diacritic"), xlevels=list(nationalism=c(-4,4)), type="dist") %>%
  mutate(
    nationalism = factor(nationalism, levels=c(4, -4), labels=c("V. high", "V. low")),
    diacritic = factor(diacritic, levels=c(TRUE, FALSE), labels=c("Present", "Absent"))
  ) %>%
  ggplot(., aes(x=diacritic, y=pred, color=nationalism, shape=nationalism)) +
    geom_point(size=2, position=position_dodge(width=0.2)) +
    geom_line(aes(x=as.integer(diacritic)), size=1, alpha=0.7, position=position_dodge(width=0.2)) +
    geom_errorbar(aes(ymin=lci, ymax=uci), size=1, width=0.3, position=position_dodge(width=0.2)) +
    xlab("Non-English characers") +
    ylab("Probability of rating") + 
    scale_color_manual(
      name = "Nationalism",
      breaks = c("V. high", "V. low"),
      values = c("firebrick3", "forestgreen")
      ) +
    scale_fill_manual(
      name = "Nationalism",
      breaks = c("V. high", "V. low"),
      values = c("firebrick3", "forestgreen")
    ) +
    scale_shape_discrete(name = "Nationalism") +
    facet_grid(. ~ response, labeller=as_labeller(function(x) str_c("Rating: ", x))) +
    ylim(0, 1) +  
    theme_bw() + 
    theme(
      legend.position="right"
    )

figS13d.mean <- clm_plotdat(modelExp2Attitudes, c("spanishExpo", "diacritic"), xlevels=list(spanishExpo=c(2,7)), type="mean") %>%
  mutate(
    exposure = factor(spanishExpo, levels=c(7, 2), labels=c("High", "V. low")),
    diacritic = factor(diacritic, levels=c(TRUE, FALSE), labels=c("Present", "Absent"))
  ) %>%
  ggplot(., aes(x=diacritic, y=pred, color=exposure, shape=exposure)) +
    geom_point(size=4, position=position_dodge(width=0.2)) +
    geom_line(aes(x=as.integer(diacritic)), size=1, alpha=0.4, position=position_dodge(width=0.2)) +
    geom_errorbar(aes(ymin=lci, ymax=uci), size=1, width=0.3, position=position_dodge(width=0.2)) +
    xlab("Non-English characters") +
    ylab("Predicted mean rating") + 
    scale_color_manual(
      name = "Spanish\nexposure",
      breaks = c("High", "V. low"),
      values = c("forestgreen", "firebrick3")
      ) +
    scale_fill_manual(
      name = "Spanish\nexposure",
      breaks = c("High", "V. low"),
      values = c("forestgreen", "firebrick3")
    ) +
    scale_shape_discrete(name = "Spanish\nexposure") +
  ylim(1.8, 4.8) +
    theme_bw() + 
    theme(
      legend.position="none"
    )

figS13d.dist <- clm_plotdat(modelExp2Attitudes, c("spanishExpo", "diacritic"), xlevels=list(spanishExpo=c(2,7)), type="dist") %>%
  mutate(
    exposure = factor(spanishExpo, levels=c(7, 2), labels=c("High", "V. low")),
    diacritic = factor(diacritic, levels=c(TRUE, FALSE), labels=c("Present", "Absent"))
  ) %>%
  ggplot(., aes(x=diacritic, y=pred, color=exposure, shape=exposure)) +
    geom_point(size=2, position=position_dodge(width=0.2)) +
    geom_line(aes(x=as.integer(diacritic)), size=1, alpha=0.7, position=position_dodge(width=0.2)) +
    geom_errorbar(aes(ymin=lci, ymax=uci), size=1, width=0.3, position=position_dodge(width=0.2)) +
    xlab("Non-English characers") +
    ylab("Probability of rating") + 
    scale_color_manual(
      name = "Spanish\nexposure",
      breaks = c("High", "V. low"),
      values = c("forestgreen", "firebrick3")
      ) +
    scale_fill_manual(
      name = "Spanish\nexposure",
      breaks = c("High", "V. low"),
      values = c("forestgreen", "firebrick3")
    ) +
    scale_shape_discrete(name = "Spanish\nexposure") +
    facet_grid(. ~ response, labeller=as_labeller(function(x) str_c("Rating: ", x))) +
    ylim(0, 1) +  
    theme_bw() + 
    theme(
      legend.position="right"
    )

ggarrange(figS13a.mean, figS13a.dist, figS13b.mean, figS13b.dist, figS13c.mean, figS13c.dist, figS13d.mean, figS13d.dist, ncol=2, labels=c("a.", "", "b.", "", "C.", "", "d.", ""), widths=c(1,5))

remove(figS13a.mean, figS13a.dist, figS13b.mean, figS13b.dist, figS13c.mean, figS13c.dist, figS13d.mean, figS13d.dist)
```

Figure S13: Partial effect plots for ordinal mixed-effects model of wellformedness ratings, as affected by self-identified attitudes and exposure. Left-hand plots show predicted mean ratings; right-hand plots show predicted distributions over ratings (facets). Figure S13a shows the interaction of phonotactic score with Spanish value (not statistically significant); Figures S13b-d show the interaction between presence of non-English characters and Spanish value, nationalism, and exposure to Spanish, respectively. Error bars represent 95% confidence intervals.

Below, we report Variance Inflation Factors for the final model, which are calculated based on the extent to which each fixed effect predictor can be predicted from all other fixed effect predictors. Since this calculation is not dependent on the random effect structures, we perform it by applying the `vif()` function from the `car` package to a (fixed-effect) logit ordinal regression model with all of the fixed effects from the final ordinal regression model; since `vif()` is not applicable to models fit with the `ordinal` package at the time of writing, we instead apply it to a model fit with the `polr()` function from the `MASS` library.

Large VIFs indicate multicollinearity, whereby one predictor is highly correlated with combinations of others, which decreases the extent to which inferences from the model can be trusted. VIFs above 5 are interpreted as potentially problematic, while VIFs above 10 are cause for serious concern.

For our model, all VIFs are below 2, which indicates low multicollinearity. Thus, the model’s results do not appear to be misled by correlations between predictors.

```
clm_vif(modelExp2Attitudes, dataExp2, caption="Variance Inflation Factors for the final model assessing interactions with attitudes and exposure from Experiment 2. All numeric variables in this model are centered.")
```

Variance Inflation Factors for the final model assessing interactions with attitudes and exposure from Experiment 2. All numeric variables in this model are centered.

| Parameter | VIF |
| --- | --- |
| scoreMorphsParsed (centered) | 1.032 |
| proSpanish (centered) | 1.244 |
| diacritic | 1.032 |
| nationalism (centered) | 1.162 |
| spanishExpo (centered) | 1.250 |
| scoreMorphsParsed (centered) × proSpanish (centered) | 1.025 |
| proSpanish (centered) × diacritic | 1.280 |
| diacritic × nationalism (centered) | 1.204 |
| diacritic × spanishExpo (centered) | 1.225 |

# 4 Appendices

## 4.1 Appendix A: Spanish orthotactics

In the Wellformedness Rating experiment, we observed that participants’ ratings increased with the phonotactic score of the nonword. We interpreted this result to indicate phonotactic knowledge of Spanish, but it is possible that participants’ ratings are actually driven by orthotactic knowledge, given the fact that stimuli were presented in written rather than auditory form. Here, we show that participants’ ratings are influenced by both phonotactics and orthotactics, but that phonotactics appears to play a larger role than orthotactics.

For simplicity, we focus on a comparison of phonotactic and orthotactic models that assume word-level representations. For phonotactics, we use the `score` that was used in Section 3.4.1. For orthotactics, we trained a trigram model over letters of unique word types in the SUBTLEX-ESP lexical database (the same word types used to train the phonotactic model), with any accents and diacritics removed, and used this model to generate Spanish orthotactic scores (`scoreSpOrth`). We excluded accents and diacritics on the assumption that, since they are not found in English, an English reader might not know how to interpret them; our statistical model still accounts for the *presence* of such accents and diacritics, but their *position* in the word is not incorporated into the orthotactic score.

As shown in the figure below, the Spanish orthotactic scores are tightly positively correlated with the Spanish (word-based) phonotactic scores (\(r=0.646\), \(p<0.001\)). This is not surprising, given the transparency of the orthography-phonology mapping; the deviations observed here are likely due to cases where an orthographic symbol is unpronounced (*h*), where a digraph corresponds to a single phoneme (e.g. *ll*), or where removing an accent or diacritic neutralizes a distinction (e.g. between *ñ* and *n*, or between *ía* and *ia*).

```
ggplot(itemsExp2, aes(x=score, y=scoreSpOrth)) +
  geom_point() +
  geom_smooth(method="lm", formula="y~x") +
  xlab("Spanish (word-based) phonotactic score") +
  ylab("Spanish orthotactic score") +
  theme_bw()
```

Spanish phonotactics and orthotactics are both positively correlated with participants’ responses in the Wellformedness Rating experiment, as shown in the figure below.

```
itemsExp2 %>%
  dplyr::select(txt, mean_response, SpPhon=score, SpOrth=scoreSpOrth) %>%
  pivot_longer(c(SpPhon, SpOrth), names_to="type", values_to="score") %>%
  mutate(
    type = factor(type, levels=c("SpPhon", "SpOrth"), labels=c("Spanish phonotactics", "Spanish orthotactics"))
  ) %>%
  ggplot(., aes(x=score, y=mean_response)) +
  geom_point() +
  geom_smooth(method="lm", formula="y~x") +
  facet_grid(~ type, scales="free_x") +
  xlab("Score") +
  ylab("Mean rating (per item)") +
  theme_bw()
```

There is some evidence that participants may be affected by *both* phonotactics and orthotactics: when both are put in a model together, both have significant positive coefficients. However, the effect of phonotactics appears to be stronger (larger coefficient) and more robust (smaller \(p\) value), as shown in the model summary below.

```
dataExp2$response <- as.factor(dataExp2$response)

# m_SpOrth_SpPhon <- clmm(
#   response ~
#     c.(score) + c.(scoreSpOrth) + diacritic +
#     (1 + c.(score) + c.(scoreSpOrth) + diacritic | workerId) + (1 | phon),
#   data=dataExp2
# )
#
# saveRDS(m_SpOrth_SpPhon, file = "dumps/clmm/modelExp2_WordTypes_SpOrthAndPhon.rds")
# remove(m_SpOrth_SpPhon)

modelWordTypes_SpOrthAndPhon <- readRDS("dumps/clmm/modelExp2_WordTypes_SpOrthAndPhon.rds")

clm_table(modelWordTypes_SpOrthAndPhon, caption="Ordinal mixed-effects model summary for well-formedness ratings, with both Spanish phonotactics (score) and Spanish orthotactics (scoreSpOrth) as predictors. Both scores are centered.")

remove(modelWordTypes_SpOrthAndPhon)
```

Ordinal mixed-effects model summary for well-formedness ratings, with both Spanish phonotactics (score) and Spanish orthotactics (scoreSpOrth) as predictors. Both scores are centered.

|  | Parameter | Estimate | Std. Error | \(z\) | \(p\) |  |
| --- | --- | --- | --- | --- | --- | --- |
| Effects | score (centered) | 1.521 | 0.460 | 3.308 | <0.001 | \*\*\* |
|  | scoreSpOrth (centered) | 0.754 | 0.319 | 2.363 | 0.018 | \* |
|  | diacritic = TRUE | 0.884 | 0.256 | 3.451 | <0.001 | \*\*\* |
| Thresholds | 1|2 | -2.483 | 0.126 |  |  |  |
|  | 2|3 | -1.018 | 0.122 |  |  |  |
|  | 3|4 | 0.045 | 0.122 |  |  |  |
|  | 4|5 | 1.864 | 0.124 |  |  |  |

Throughout the analyses presented here, we have tried to identify a single phonotactic score that best explains participants’ ratings. In the same vein, we can compare the phonotactic and orthotactic scores and ask: if participants’ knowledge is coming from just one of them, which one is more likely to underpin the pattern of responses seen in the Wellformedness Rating experiment? To do so, we construct a mixed-effects ordinal regression model utilizing orthotactic score, by substituting orthotactic score for phonotactic score in the existing word-based model. We compare the AIC scores of this orthotactic word-based model and the original phonotactic word-based model. As shown in the table below, the phonotactic model has lower AIC score, indicating that it explains participants’ ratings better than the orthotactic model. This result accords with the previous one, suggesting that phonotactics plays a larger role than orthotactics.

```
# m_SpOrth <- clmm(
#   response ~
#     c.(scoreSpOrth) + diacritic +
#     (1 + c.(scoreSpOrth) + diacritic | workerId) + (1 | phon),
#   data=dataExp2
# )
#
# saveRDS(m_SpOrth, file = "dumps/clmm/modelExp2_WordTypes_SpOrth.rds")
# remove(m_SpOrth)

modelWordTypes_SpOrth <- readRDS("dumps/clmm/modelExp2_WordTypes_SpOrth.rds")
modelWordTypes <- readRDS("dumps/clmm/modelExp2_WordTypes.rds")

aics = data.frame("Score" = c("Spanish phonotactics", "Spanish orthotactics"),
                  "AIC" = c(AIC(modelWordTypes), AIC(modelWordTypes_SpOrth)))

display_table(aics, digits=1, highlight=c("AIC"), caption="Comparison of AIC across models predicting participants' wellformedness ratings from either Spanish phonotactic scores or Spanish orthotactic scores")

remove(modelWordTypes_SpOrth, modelWordTypes)
```

Comparison of AIC across models predicting participants’ wellformedness ratings from either Spanish phonotactic scores or Spanish orthotactic scores

| Score | AIC |
| --- | --- |
| Spanish phonotactics | 26902.2 |
| Spanish orthotactics | 26917.7 |

This analysis is preliminary; more comprehensive analyses of the precise nature of the knowledge that participants use to underpin their ratings – including the extent to which it is phonotactic vs. orthotactic and the extent to which it reflects accurate and complete mapping of orthography to phonology – would be valuable, but are beyond the scope of the present work. Nevertheless, this preliminary analysis suggests that participants do, indeed, make (primary) use of phonotactic knowledge in the Wellformedness Rating experiments, and thus that our conclusions are not adversely affected by the use of written stimuli and do not hinge on uncertain assumptions about participants’ ability to map between orthographic and phonological forms.

## 4.2 Appendix B: English orthotactics

In the Wellformedness Rating experiment, we observed that participants’ ratings increased with the phonotactic score of the nonword. We interpreted this result to indicate phonotactic knowledge of Spanish, but it is possible that participants’ ratings are actually driven by their experience with English, not Spanish. Here, we show that the patterns we have observed cannot be explained away by knowledge of English.

For simplicity, we focus on a comparison between knowledge of English word types and knowledge of Spanish word types. If the word-based Spanish model explains participants’ ratings better than the word-based English model, we conclude that participants have phonotactic knowledge of Spanish. This conclusion is not affected by the fact that our final Spanish model was based on morphs, not words, because that model peformed even better than the Spanish word-based model.

We developed an English orthotactic model by training a trigram model over letters of unique word types in the CMU Pronouncing Dictionary (excluding any word types that contained non-alphabetic characters, such as hyphens and apostrophes). We chose to use an orthotactic rather than phonotactic model in order to limit assumptions about how participants may interpret Spanish words within an English phonological framework, given the complex and ambiguous mapping between orthography and phonology in English. We used this model to generate English orthotactic scores for our stimuli in the same way as we previously generated (word-based) phonotactic scores, where the stimuli were presented to the model in orthographic form rather than phonological form (with Spanish diacritics removed, since they do not occur in the English training data).

As shown in the figure below, the English orthotactic scores are positively correlated with the Spanish (word-based) phonotactic scores (\(r=0.347\), \(p<0.01\)).

```
ggplot(itemsExp2, aes(x=score, y=scoreEngOrth)) +
  geom_point() +
  geom_smooth(method="lm", formula="y~x") +
  xlab("Spanish (word-based) phonotactic score") +
  ylab("English orthotactic score") +
  theme_bw()
```

English orthotactics and Spanish phonotactics appear to have different relationships with participants’ ratings in visualizations of the raw data: participants’ ratings *increase* with Spanish phonotactic score, and *decrease* with English orthotactic score. This suggests that participants may give the appearance of increasing ratings with Spanish phonotactics when in fact they are decreasing ratings with English orthotactics, following the assumption that something is “Spanish-like” if it is *not* “English-like”. However, the fact that Spanish phonotactics and English orthotactics are *positively* correlated with each other makes any such assumption unlikely to give good explanation of participants’ ratings.

```
itemsExp2 %>%
  dplyr::select(txt, mean_response, SpPhon=score, EngOrth=scoreEngOrth) %>%
  pivot_longer(c(SpPhon, EngOrth), names_to="type", values_to="score") %>%
  mutate(
    type = factor(type, levels=c("SpPhon", "EngOrth"), labels=c("Spanish phonotactics", "English orthotactics"))
  ) %>%
  ggplot(., aes(x=score, y=mean_response)) +
  geom_point() +
  geom_smooth(method="lm", formula="y~x") +
  facet_grid(~ type, scales="free_x") +
  xlab("Score") +
  ylab("Mean rating (per item)") +
  theme_bw()
```

To confirm that participants’ behaviors do not just boil down to rating non-English-like items as Spanish-like, we take the word-based ordinal regression model for participants’ ratings and add a predictor for English orthotactic score wherever there is a predictor for Spanish phonotactic score. The results summarized in the table below indicate that *both* Spanish phonotactics and English orthotactics have significant effects on participants’ ratings: items receive a higher rating the *higher* their Spanish phonotactic score and the *lower* their English orthotactic score. Since Spanish phonotactics plays an independent role to English orthotactics, we conclude that participants *do* have Spanish phonotactic knowledge: their ratings cannot be explained solely by English orthotactics.

```
dataExp2$response <- as.factor(dataExp2$response)

# m_both <- clmm(
#   response ~
#     c.(score) + c.(scoreEngOrth) + diacritic +
#     (1 + c.(score) + c.(scoreEngOrth) + diacritic | workerId) + (1 | phon),
#   data=dataExp2
# )
#
# saveRDS(m_both, file = "dumps/clmm/modelExp2_WordTypes_SpAndEng.rds")
# remove(m_both)

modelWordTypes_SpAndEng <- readRDS("dumps/clmm/modelExp2_WordTypes_SpAndEng.rds")

clm_table(modelWordTypes_SpAndEng, caption="Ordinal mixed-effects model summary for well-formedness ratings, with both Spanish phonotactics (score) and English orthotactics (scoreEngOrth) as predictors. Both scores are centered.")

remove(modelWordTypes_SpAndEng)
```

Ordinal mixed-effects model summary for well-formedness ratings, with both Spanish phonotactics (score) and English orthotactics (scoreEngOrth) as predictors. Both scores are centered.

|  | Parameter | Estimate | Std. Error | \(z\) | \(p\) |  |
| --- | --- | --- | --- | --- | --- | --- |
| Effects | score (centered) | 2.919 | 0.490 | 5.952 | <0.001 | \*\*\* |
|  | scoreEngOrth (centered) | -1.163 | 0.275 | -4.233 | <0.001 | \*\*\* |
|  | diacritic = TRUE | 0.940 | 0.254 | 3.696 | <0.001 | \*\*\* |
| Thresholds | 1|2 | -2.492 | 0.126 |  |  |  |
|  | 2|3 | -1.019 | 0.123 |  |  |  |
|  | 3|4 | 0.048 | 0.122 |  |  |  |
|  | 4|5 | 1.875 | 0.124 |  |  |  |

Our interpretation assumes that Spanish phonotactic knowledge is the *primary* explanation of participants’ wellformedness ratings. To test this assumption, we construct ordinal regression models using *either* Spanish phonotactic scores *or* English orthotactic scores, and compare their ability to explain participants’ ratings, as measured by AIC. The model containing Spanish phonotactic scores has a much lower AIC score, indicating that it explains participants’ ratings better than English orthotactic scores (when both are considered in isolation).

```
# m_Eng <- clmm(
#   response ~
#     c.(scoreEngOrth) + diacritic +
#     (1 + c.(scoreEngOrth) + diacritic | workerId) + (1 | phon),
#   data=dataExp2
# )
#
# saveRDS(m_Eng, file = "dumps/clmm/modelExp2_WordTypes_Eng.rds")
# remove(m_Eng)

modelWordTypes_Eng <- readRDS("dumps/clmm/modelExp2_WordTypes_Eng.rds")
modelWordTypes <- readRDS("dumps/clmm/modelExp2_WordTypes.rds")

aics = data.frame("Score" = c("Spanish phonotactics", "English orthotactics"),
                  "AIC" = c(AIC(modelWordTypes), AIC(modelWordTypes_Eng)))

display_table(aics, digits=1, highlight=c("AIC"), caption="Comparison of AIC across models predicting participants' wellformedness ratings from either Spanish phonotactic scores or English orthotactic scores")

remove(modelWordTypes_Eng, modelWordTypes)
```

Comparison of AIC across models predicting participants’ wellformedness ratings from either Spanish phonotactic scores or English orthotactic scores

| Score | AIC |
| --- | --- |
| Spanish phonotactics | 26902.2 |
| English orthotactics | 26970.8 |

## 4.3 Appendix C: attitude constructs

In our analysis, we constructed *Spanish value* and *nationalism* scores manually, by adding together participants’ responses on two questions each. We mentioned that this approach was justified by an exploratory factor analysis of responses to the 4 attitude questions. Here, we show the details of that exploratory analysis, to elucidate the validity of our Spanish value and nationalism constructs.

For this analysis, we use the data from all unique participants that were analyzed for either experiment. This means we use the data from 66 unique participants: 40 analyzed in Experiment 1, and 26 analyzed in Experiment 2 that were not already analyzed in Experiment 1 (13 participants were analyzed in both experiments).

We use the `psych` package (with default settings) to explore different numbers of factors that could underpin structured variation in participants’ responses to the 4 attitude questions, where the calculation of factors is based on polychoric correlations. Given that the attitude questions were designed to access 2 distinct properties – Spanish value and nationalism – we are particularly interested in the extent to which 2 principal components capture covariation across the 4 questions. A parallel analysis conducted with the `fa.parallel()` function suggests that 2 factors is indeed best: the top 2 factors of the actual data explain more variance than do the top 2 factors of random data, and adding additional factors does not explain substantially more variance. (Note: `fa.parallel()` reports warnings indicating that factor scores may be incorrect; these issues arise from solutions involving more than 2 factors, and thus strengthen our conclusions.)

```
attitudes = rbind(
    participantsExp1, 
    participantsExp2 %>% anti_join(participantsExp1, by="workerId")
  ) %>%
  dplyr::select(proSpanishLang, proSpanishCult, proEnglishUSA, proImmigration)

fa.parallel(attitudes, cor="poly", fa="fa")

factors = fa(attitudes, nfactors=2, cor="poly")
```

```
## Parallel analysis suggests that the number of factors =  2  and the number of components =  NA
```

Together, the two factors account for 48% of the common variance among responses to different questions: the first factor (MR1) accounts for 28.4% of variance, and the second factor (MR2) accounts for 19.5% of variance. The breakdown of the variance explained by the two factors across different questions (i.e. the communalities) is as follows:

```
data.frame(Question=names(factors$communality), Communality=as.vector(factors$communality)) %>%
  kable(digits=3, escape=F, align="lr") %>%
  kable_styling()
```

| Question | Communality |
| --- | --- |
| proSpanishLang | 0.267 |
| proSpanishCult | 0.709 |
| proEnglishUSA | 0.496 |
| proImmigration | 0.447 |

The loadings of the questions onto the factors are shown in the biplot below, and summarized in the factor diagram. As can be seen, MR1 underpins responses to `proSpanishLang` and `proSpanishCult`: the larger a participant’s latent value on the MR1 dimension, the higher they will rate Spanish language and culture. MR1 therefore corresponds to the construct of Spanish value we had in mind when we designed the questions. MR2 underpins responses to `proEnglishUSA` and `proImmigration`: the smaller a participant’s latent value on the MR2 dimension, the more they will support English as the language of the US, and the less they will support immigration. MR2 therefore corresponds (with reversed polarity) to the construct of nationalism that we had in mind when we designed the questions. From this, we conclude that our questions were effective at mapping onto the constructs of Spanish value and nationalism, as intended.

```
biplot(factors)
fa.diagram(factors)
```

Finally, we note – as summarized in the factor diagram – that for each factor, the two questions that load onto that factor have similar scores; that is, that they are affected to similar extents by the underlying latent factor (i.e. with similar magnitude, disregarding sign). For MR1, we see a score of 0.8 for `proSpanishCult` and 0.5 for `proSpanishLang`; for MR2, we see a score of 0.7 for `proImmigration` and \(-0.5\) for `proEnglishUSA`. This indicates that *both* questions in each case are reflective of the underlying construct, to similar degrees, which supports our decision to weight them equally in our manually-designed calculations of Spanish value and nationalism.

Ultimately, we take the exploratory factor analysis to indicate the appropriateness of our questionnaire design and of our use of questionnaire responses in the statistical analysis. We do not use the inferred factors in the statistical analysis itself, because they are based on the precise set of participants that completed the experiments, and thus may not facilitate interpretation and generalization. Instead, we use the manually-designed scores for Spanish value and nationalism as proxies for the underlying constructs discussed here, which are justified by the findings of the exploratory factor analysis; these manually-designed scores are advantageous because they will not vary if the experimental data are reanalyzed with different inclusion criteria, and they will extend straightforwardly to new data collected in the future to facilitate replication and meta-analytical comparison.

## 4.4 Appendix D: Spanish value without language policy

To measure Spanish value, we combined responses to two questions: one about language policy that would make some Spanish language education compulsory, and one about the importance of Hispanic and Latino cultures. However, the political and authoritarian overtones of language policy may have affected responses to the first question, causing it to measure something different to what we intended. Here, we explore whether this might have affected the results we report for Spanish value.

While responses to the two questions exhibit polychoric correlations in both the Word Identification and Wellformedness Rating experiments (Word Identification \(\rho=0.403\), Wellformedness Rating \(\rho=0.501\)), it is clear upon inspection that the language policy question elicited a wider range of responses than the cultural importance question. As shown in the bubble plots below, many participants gave ratings indicating opposition to language policy, but few gave ratings indicating low cultural importance.

```
participantsExp1 %>% 
  mutate(experiment = "Exp1: Word identification") %>%
  rbind(participantsExp2 %>%
          mutate(experiment = "Exp2: Wellformedness ratings")) %>%
  dplyr::count(across(c(experiment, proSpanishLang, proSpanishCult)), .drop=FALSE) %>%
  ggplot(., aes(x=proSpanishLang, y=proSpanishCult)) +
  geom_hline(yintercept=3, linetype="dashed", color="darkgray", size=2) +
  geom_vline(xintercept=3, linetype="dashed", color="darkgray", size=2) +
  geom_smooth(data=participantsExp1, method="loess", formula="y~x", alpha=0.3) +
  geom_point(aes(size=n)) +
  scale_size_continuous(name="Participants", range=c(2,9), breaks=1:8) +
  xlab("Response to language policy question") +
  ylab("Response to cultural importance question") +
  coord_cartesian(xlim=c(1, 5), ylim=c(1, 5)) +
  facet_grid(~ experiment) +
  theme_bw() +
  theme(
    panel.grid = element_blank(),
    axis.title = element_text(size=16, color="black"),
    axis.text = element_text(size=14, color="black"),
    legend.title = element_text(size=16, color="black"),
    legend.text = element_text(size=15, color="black"),
    strip.text = element_text(size=18, color="black")
  )
```

Given this observation, it is important to analyze the extent to which the results for Spanish value may have been driven by positionalities on language policy that have more to do with political and educational views than they do with attitudes toward Spanish and its speakers per se. To do so, we substitute responses to the cultural importance question (`proSpanishCult`) in place of our combined measure (`proSpanish`) in the final attitude-based model for each experiment, and we analyze whether the reported patterns still hold.

The results for the Word Identification experiment are presented in the table below. To aid with interpretation, the partial effects are also plotted (following the same designation of variables to plot as in the original presentation of results, to facilitate comparison). Compared to the original model, the interaction between Spanish attitude and phonotactic score is no longer significant (though still exhibits a strong numerical trend in the same direction), but the crucial interaction between Spanish attitude and stimulus type remains, as does the three-way interaction between attitude, stimulus type, and the high-frequency bin. Thus, the key observation remains intact: as participants’ Spanish value increases, they rate real words higher than nonwords, especially for high-frequency words. In other words, the results for Spanish value in the Word Identification experiment were *not* driven by positionalities on language policy.

```
# Download file from Google drive if it doesn't already exist (it is too large for GitHub)
if (!file.exists("dumps/clmm/modelExp1_AttitudesCult.rds")) {
  download.file("https://drive.google.com/uc?export=download&id=1Od_noImdCmXSGLezepZDCTtoeJsUI07s&confirm=t", "dumps/clmm/modelExp1_AttitudesCult.rds")
}
modelExp1AttitudesCult = readRDS("dumps/clmm/modelExp1_AttitudesCult.rds")

clm_table(modelExp1AttitudesCult, caption="Ordinal mixed-effects model of wordhood confidence ratings, using perceived importance of Hispanic and Latino cultures as a measure of Spanish value. All numeric predictors are centered, and frequency bin is Helmert-coded.")
```

Ordinal mixed-effects model of wordhood confidence ratings, using perceived importance of Hispanic and Latino cultures as a measure of Spanish value. All numeric predictors are centered, and frequency bin is Helmert-coded.

|  | Parameter | Estimate | Std. Error | \(z\) | \(p\) |  |
| --- | --- | --- | --- | --- | --- | --- |
| Effects | score (centered) | 1.190 | 0.362 | 3.286 | 0.001 | \*\* |
|  | type = real | 0.472 | 0.094 | 5.035 | <0.001 | \*\*\* |
|  | freq = mid.v.low | 0.060 | 0.109 | 0.553 | 0.580 |  |
|  | freq = high.v.others | -0.070 | 0.102 | -0.692 | 0.489 |  |
|  | proSpanishCult (centered) | -0.174 | 0.134 | -1.299 | 0.194 |  |
|  | diacritic = TRUE | 1.195 | 0.273 | 4.386 | <0.001 | \*\*\* |
|  | nationalism (centered) | 0.095 | 0.059 | 1.598 | 0.110 |  |
|  | spanishExpo (centered) | 0.200 | 0.094 | 2.124 | 0.034 | \* |
|  | type = real × freq = mid.v.low | 0.059 | 0.160 | 0.367 | 0.714 |  |
|  | type = real × freq = high.v.others | 0.532 | 0.147 | 3.610 | <0.001 | \*\*\* |
|  | score (centered) × proSpanishCult (centered) | 0.501 | 0.310 | 1.617 | 0.106 |  |
|  | type = real × proSpanishCult (centered) | 0.309 | 0.080 | 3.842 | <0.001 | \*\*\* |
|  | freq = mid.v.low × proSpanishCult (centered) | -0.028 | 0.089 | -0.313 | 0.755 |  |
|  | freq = high.v.others × proSpanishCult (centered) | -0.073 | 0.082 | -0.888 | 0.375 |  |
|  | type = real × freq = mid.v.low × proSpanishCult (centered) | 0.092 | 0.131 | 0.700 | 0.484 |  |
|  | type = real × freq = high.v.others × proSpanishCult (centered) | 0.379 | 0.117 | 3.246 | 0.001 | \*\* |
| Thresholds | 1|2 | -2.760 | 0.144 |  |  |  |
|  | 2|3 | -0.965 | 0.138 |  |  |  |
|  | 3|4 | 0.507 | 0.138 |  |  |  |
|  | 4|5 | 2.850 | 0.143 |  |  |  |

```
figExp1Culta.mean <- clm_plotdat(modelExp1AttitudesCult, c("type", "freq", "proSpanishCult"), xlevels=list(proSpanishCult=c(1, 5)), type="mean") %>%
  mutate(
    type = fct_recode(type, "Nonword"="pseudo", "Word"="real") %>% fct_relevel("Word"),
    attitude = factor(proSpanishCult, levels=c(5, 1), labels=c("Attitude: v. pos.", "Attitude: v. neg."))
  ) %>%
  ggplot(., aes(x=as.integer(freq), y=pred, color=type, fill=type, shape=type)) +
    geom_ribbon(aes(ymin=lci, ymax=uci), alpha=0.1, color=NA) +
    geom_line(size=1, alpha=0.4) +
    geom_point(size=4) +
    geom_errorbar(aes(ymin=lci, ymax=uci), size=1, width=0.3) +
    scale_x_continuous(name="Frequency Bin", breaks=1:3, labels=c("Low", "Mid", "High")) +
    ylab("Predicted mean rating") +
    # ylim(2.9, 4.1) +
    facet_grid(attitude ~ ., switch="y") +
    scale_shape_manual(values = c("Nonword" = 19, "Word" = 17)) +
    scale_color_manual(values = c("Nonword" = "black", "Word" = "blue")) +
    scale_fill_manual(values = c("Nonword" = "black", "Word" = "blue")) +
    theme_bw() +
    theme(
      legend.position="none",
      legend.title=element_blank()
    )

figExp1Culta.dist <- clm_plotdat(modelExp1AttitudesCult, c("type", "freq", "proSpanishCult"), xlevels=list(proSpanishCult=c(1, 5)), type="dist") %>%
  mutate(
    type = fct_recode(type, "Nonword"="pseudo", "Word"="real") %>% fct_relevel("Word"),
    attitude = factor(proSpanishCult, levels=c(5, 1), labels=c("Attitude: v. pos.", "Attitude: v. neg.")),
    Response = paste("Rating:", response)
  ) %>%
  ggplot(aes(x=as.integer(freq), y=pred, color=type, fill=type, shape=type)) +
    geom_ribbon(aes(ymin=lci, ymax=uci), alpha=0.2, color=NA) +
    geom_line(size=1, alpha=0.4) +
    geom_point(size=2) +
    geom_errorbar(aes(ymin=lci, ymax=uci), size=1, width=0.3) +
    facet_grid(attitude ~ Response, switch="y") +
    ylim(0, 1) +
    scale_x_continuous(name="Frequency Bin", breaks=1:3, labels=c("Low", "Mid", "High")) +
    ylab("Probability of rating") +
    scale_shape_manual(values = c("Nonword" = 19, "Word" = 17)) +
    scale_color_manual(values = c("Nonword" = "black", "Word" = "blue")) +
    scale_fill_manual(values = c("Nonword" = "black", "Word" = "blue")) +
    theme_bw() +
    theme(
      legend.position="right",
      legend.title=element_blank()
    )

figExp1Cultb.mean <- clm_plotdat(modelExp1AttitudesCult, c("score", "proSpanishCult"), xlevels=list(proSpanishCult=c(1, 5), score=25), type="mean") %>%
  mutate(
    type="Word and\nNonword",
    attitude = factor(proSpanishCult, levels=c(5, 1), labels=c("Attitude: v. pos.", "Attitude: v. neg."))
  ) %>%
  ggplot(., aes(x=score, y=pred, color=type, fill=type)) +
    geom_ribbon(aes(ymin=lci, ymax=uci), alpha=0.3, color=NA) +
    geom_line(size=1) +
    scale_x_continuous(n.breaks=4) +
    xlab("Phonotactic score") +
    ylab("Predicted mean rating") +
    # ylim(2.9, 4.1) +
    facet_grid(attitude ~ ., switch="y") +
    scale_color_manual(values = c("Word and\nNonword" = "purple")) +
    scale_fill_manual(values = c("Word and\nNonword" = "purple")) +
    theme_bw() +
    theme(
      legend.position="none",
      legend.title=element_blank()
    )

figExp1Cultb.dist <- clm_plotdat(modelExp1AttitudesCult, c("score", "proSpanishCult"), xlevels=list(proSpanishCult=c(1, 5), score=25), type="dist") %>%
  mutate(
    type="Word and\nNonword",
    attitude = factor(proSpanishCult, levels=c(5, 1), labels=c("Attitude: v. pos.", "Attitude: v. neg.")),
    Response = paste("Rating:", response)
  ) %>%
  ggplot(., aes(x=score, y=pred, color=type, fill=type)) +
    geom_ribbon(aes(ymin=lci, ymax=uci), alpha=0.3, color=NA) +
    geom_line(size=1) +
    scale_x_continuous(n.breaks=4) +
    xlab("Phonotactic score") +
    ylab("Probability of rating") +
    scale_color_manual(values = c("Word and\nNonword" = "purple")) +
    scale_fill_manual(values = c("Word and\nNonword" = "purple")) +
    facet_grid(attitude ~ Response, switch="y") +
    ylim(0, 1) +
    theme_bw() +
    theme(
      legend.position="right",
      legend.title=element_blank()
    )

figExp1Cultc.mean <- clm_plotdat(modelExp1AttitudesCult, "diacritic", type="mean") %>%
  mutate(
    type = "Word and\nNonword",
    diacritic = factor(diacritic, levels=c(TRUE, FALSE), labels=c("Present", "Absent"))
  ) %>%
  ggplot(., aes(x=diacritic, y=pred, color=type, fill=type)) +
    geom_point(size=4) +
    geom_errorbar(aes(ymin=lci, ymax=uci), size=1, width=0.3) +
    xlab("Non-English characters") +
    ylab("Predicted mean rating") +
    ylim(2.9, 4.1) +
    scale_color_manual(values = c("Word and\nNonword" = "purple")) +
    scale_fill_manual(values = c("Word and\nNonword" = "purple")) +
    theme_bw() +
    theme(
      legend.position="none",
      legend.title=element_blank()
    )

figExp1Cultc.dist <- clm_plotdat(modelExp1AttitudesCult, "diacritic", type="dist") %>%
  mutate(
    type = "Word and\nNonword",
    diacritic = factor(diacritic, levels=c(TRUE, FALSE), labels=c("Present", "Absent"))
  ) %>%
  ggplot(., aes(x=diacritic, y=pred, color=type, fill=type)) +
    geom_point(size=4) +
    geom_errorbar(aes(ymin=lci, ymax=uci), size=1, width=0.3) +
    xlab("Non-English characters") +
    ylab("Probability of rating") +
    scale_color_manual(values = c("Word and\nNonword" = "purple")) +
    scale_fill_manual(values = c("Word and\nNonword" = "purple")) +
    facet_grid(. ~ response, labeller=as_labeller(function(x) str_c("Rating: ", x))) +
    ylim(0, 1) +
    theme_bw() +
    theme(
      legend.position="right",
      legend.title=element_blank()
    )

ggarrange(figExp1Culta.mean, figExp1Culta.dist, figExp1Cultb.mean, figExp1Cultb.dist, figExp1Cultc.mean, figExp1Cultc.dist, ncol=2, labels=c("a.", "", "b.", "", "c.", ""), widths=c(1,5), heights=c(2,2,1))

remove(figExp1Culta.mean, figExp1Culta.dist, figExp1Cultb.mean, figExp1Cultb.dist, figExp1Cultc.mean, figExp1Cultc.dist)

remove(modelExp1AttitudesCult)
```

Partial effect plots for ordinal mixed-effects model of wordhood confidence ratings, as affected by perceived importance of Hispanic and Latino cultures. Left-hand plots show predicted mean ratings; right-hand plots show predicted distributions over ratings (facets). Top panel shows the interaction between frequency bin, lexicality (real words vs. nonwords), and attitude; middle panel shows the interaction between phonotactic score and attitude; bottom panel shows the effect of presence or absence of non-English characters. Error bars and ribbons represent 95% confidence intervals.

The results for the Wellformedness Rating experiment are presented in the table below, together with partial effects plots. Compared to the original model, the interaction between Spanish attitude and presence of a diacritic is no longer significant, but we did not interpret that interaction anyway. More importantly, the crucial interaction between Spanish attitude and phonotactic score, which exhibited a strong numerical trend that was nevertheless not significant in the original model, is significant here. Thus, the key claim that we made based on the original results is, in fact, strengthened by disregarding positionalities on language policy: as participants’ Spanish value increases, their sensitivity to Spanish phonotactics in this task increases.

```
modelExp2AttitudesCult <- readRDS("dumps/clmm/modelExp2_AttitudesCult_withScoreInteraction.rds")

clm_table(modelExp2AttitudesCult, caption="Ordinal mixed-effects model of wellformedness ratings, using perceived importance of Hispanic and Latino cultures as a measure of Spanish value. All numeric predictors are centered, and frequency bin is Helmert-coded.")
```

Ordinal mixed-effects model of wellformedness ratings, using perceived importance of Hispanic and Latino cultures as a measure of Spanish value. All numeric predictors are centered, and frequency bin is Helmert-coded.

|  | Parameter | Estimate | Std. Error | \(z\) | \(p\) |  |
| --- | --- | --- | --- | --- | --- | --- |
| Effects | scoreMorphsParsed (centered) | 3.399 | 0.588 | 5.783 | <0.001 | \*\*\* |
|  | proSpanishCult (centered) | 0.000 | 0.113 | -0.001 | 0.999 |  |
|  | diacritic = TRUE | 0.927 | 0.220 | 4.224 | <0.001 | \*\*\* |
|  | nationalism (centered) | 0.063 | 0.052 | 1.210 | 0.226 |  |
|  | spanishExpo (centered) | -0.035 | 0.085 | -0.414 | 0.679 |  |
|  | scoreMorphsParsed (centered) × proSpanishCult (centered) | 1.300 | 0.483 | 2.694 | 0.007 | \*\* |
|  | proSpanishCult (centered) × diacritic = TRUE | -0.304 | 0.204 | -1.490 | 0.136 |  |
|  | diacritic = TRUE × nationalism (centered) | -0.166 | 0.090 | -1.845 | 0.065 | . |
|  | diacritic = TRUE × spanishExpo (centered) | -0.338 | 0.143 | -2.368 | 0.018 | \* |
| Thresholds | 1|2 | -2.510 | 0.125 |  |  |  |
|  | 2|3 | -1.024 | 0.121 |  |  |  |
|  | 3|4 | 0.052 | 0.121 |  |  |  |
|  | 4|5 | 1.896 | 0.123 |  |  |  |

```
figExp2Culta.mean <- clm_plotdat(modelExp2AttitudesCult, c("scoreMorphsParsed", "proSpanishCult"), xlevels=list(scoreMorphsParsed=25, proSpanishCult=c(1, 5)), type="mean") %>%
  mutate(
    value = factor(proSpanishCult, levels = c(5, 1), labels=c("V. pos.", "V. neg."))
  ) %>%
  ggplot(., aes(x=scoreMorphsParsed, y=pred, color=value, fill=value)) +
    geom_ribbon(aes(ymin=lci, ymax=uci), alpha=0.3, color=NA) +
    geom_line(size=1) +
    xlab("Phonotactic score") +
    ylab("Predicted mean rating") + 
    scale_color_manual(
      name = "Spanish\nvalue",
      breaks = c("V. pos.", "V. neg."),
      values = c("forestgreen", "firebrick3")
      ) +
    scale_fill_manual(
      name = "Spanish\nvalue",
      breaks = c("V. pos.", "V. neg."),
      values = c("forestgreen", "firebrick3")
    ) +
    scale_x_continuous(n.breaks=4) +
    ylim(1.8, 4.8) +
    theme_bw() + 
    theme(
      legend.position="none"
    )

figExp2Culta.dist <- clm_plotdat(modelExp2AttitudesCult, c("scoreMorphsParsed", "proSpanishCult"), xlevels=list(scoreMorphsParsed=25, proSpanishCult=c(1, 5)), type="dist") %>%
  mutate(
    value = factor(proSpanishCult, levels = c(5, 1), labels=c("V. pos.", "V. neg."))
  ) %>%
  ggplot(., aes(x=scoreMorphsParsed, y=pred, color=value, fill=value)) +
    geom_ribbon(aes(ymin=lci, ymax=uci), alpha=0.3, color=NA) +
    geom_line(size=1) +
    xlab("Phonotactic score") +
    ylab("Probability of rating") + 
    scale_x_continuous(n.breaks=4) +
    scale_color_manual(
      name = "Spanish\nvalue",
      breaks = c("V. pos.", "V. neg."),
      values = c("forestgreen", "firebrick3")
      ) +
    scale_fill_manual(
      name = "Spanish\nvalue",
      breaks = c("V. pos.", "V. neg."),
      values = c("forestgreen", "firebrick3")
    ) +
    facet_grid(. ~ response, labeller=as_labeller(function(x) str_c("Rating: ", x))) +
    ylim(0, 1) +  
    theme_bw() + 
    theme(
      legend.position="right"
    )

figExp2Cultb.mean <- clm_plotdat(modelExp2AttitudesCult, c("proSpanishCult", "diacritic"), xlevels=list(proSpanishCult=c(1, 5)), type="mean") %>%
  mutate(
    value = factor(proSpanishCult, levels = c(5, 1), labels=c("V. pos.", "V. neg.")),
    diacritic = factor(diacritic, levels=c(TRUE, FALSE), labels=c("Present", "Absent"))
  ) %>%
  ggplot(., aes(x=diacritic, y=pred, color=value, shape=value)) +
    geom_point(size=4, position=position_dodge(width=0.2)) +
    geom_line(aes(x=as.integer(diacritic)), size=1, alpha=0.4, position=position_dodge(width=0.2)) +
    geom_errorbar(aes(ymin=lci, ymax=uci), size=1, width=0.3, position=position_dodge(width=0.2)) +
    xlab("Non-English characters") +
    ylab("Predicted mean rating") + 
    scale_color_manual(
      name = "Spanish\nvalue",
      breaks = c("V. pos.", "V. neg."),
      values = c("forestgreen", "firebrick3")
      ) +
    scale_fill_manual(
      name = "Spanish\nvalue",
      breaks = c("V. pos.", "V. neg."),
      values = c("forestgreen", "firebrick3")
    ) +
    scale_shape_discrete(name = "Spanish\nvalue") +
    ylim(1.8, 4.8) +
    theme_bw() + 
    theme(
      legend.position="none"
    )

figExp2Cultb.dist <- clm_plotdat(modelExp2AttitudesCult, c("proSpanishCult", "diacritic"), xlevels=list(proSpanishCult=c(1, 5)), type="dist") %>%
  mutate(
    value = factor(proSpanishCult, levels = c(5, 1), labels=c("V. pos.", "V. neg.")),
    diacritic = factor(diacritic, levels=c(TRUE, FALSE), labels=c("Present", "Absent"))
  ) %>%
  ggplot(., aes(x=diacritic, y=pred, color=value, shape=value)) +
    geom_point(size=2, position=position_dodge(width=0.2)) +
    geom_line(aes(x=as.integer(diacritic)), size=1, alpha=0.7, position=position_dodge(width=0.2)) +
    geom_errorbar(aes(ymin=lci, ymax=uci), size=1, width=0.3, position=position_dodge(width=0.2)) +
    xlab("Non-English characers") +
    ylab("Probability of rating") + 
    scale_color_manual(
      name = "Spanish\nvalue",
      breaks = c("V. pos.", "V. neg."),
      values = c("forestgreen", "firebrick3")
      ) +
    scale_fill_manual(
      name = "Spanish\nvalue",
      breaks = c("V. pos.", "V. neg."),
      values = c("forestgreen", "firebrick3")
    ) +
    scale_shape_discrete(name = "Spanish\nvalue") +
    facet_grid(. ~ response, labeller=as_labeller(function(x) str_c("Rating: ", x))) +
    ylim(0, 1) +  
    theme_bw() + 
    theme(
      legend.position="right"
    )

figExp2Cultc.mean <- clm_plotdat(modelExp2AttitudesCult, c("nationalism", "diacritic"), xlevels=list(nationalism=c(-4,4)), type="mean") %>%
  mutate(
    nationalism = factor(nationalism, levels = c(4, -4), labels=c("V. high", "V. low")),
    diacritic = factor(diacritic, levels=c(TRUE, FALSE), labels=c("Present", "Absent"))
  ) %>%
  ggplot(., aes(x=diacritic, y=pred, color=nationalism, shape=nationalism)) +
    geom_point(size=4, position=position_dodge(width=0.2)) +
    geom_line(aes(x=as.integer(diacritic)), size=1, alpha=0.4, position=position_dodge(width=0.2)) +
    geom_errorbar(aes(ymin=lci, ymax=uci), size=1, width=0.3, position=position_dodge(width=0.2)) +
    xlab("Non-English characters") +
    ylab("Predicted mean rating") + 
    scale_color_manual(
      name = "Nationalism",
      breaks = c("V. high", "V. low"),
      values = c("firebrick3", "forestgreen")
      ) +
    scale_fill_manual(
      name = "Nationalism",
      breaks = c("V. high", "V. low"),
      values = c("firebrick3", "forestgreen")
    ) +
    scale_shape_discrete(name = "Nationalism") +
    ylim(1.8, 4.8) +
    theme_bw() + 
    theme(
      legend.position="none"
    )

figExp2Cultc.dist <- clm_plotdat(modelExp2AttitudesCult, c("nationalism", "diacritic"), xlevels=list(nationalism=c(-4,4)), type="dist") %>%
  mutate(
    nationalism = factor(nationalism, levels = c(4, -4), labels=c("V. high", "V. low")),
    diacritic = factor(diacritic, levels=c(TRUE, FALSE), labels=c("Present", "Absent"))
  ) %>%
  ggplot(., aes(x=diacritic, y=pred, color=nationalism, shape=nationalism)) +
    geom_point(size=2, position=position_dodge(width=0.2)) +
    geom_line(aes(x=as.integer(diacritic)), size=1, alpha=0.7, position=position_dodge(width=0.2)) +
    geom_errorbar(aes(ymin=lci, ymax=uci), size=1, width=0.3, position=position_dodge(width=0.2)) +
    xlab("Non-English characers") +
    ylab("Probability of rating") + 
    scale_color_manual(
      name = "Nationalism",
      breaks = c("V. high", "V. low"),
      values = c("firebrick3", "forestgreen")
      ) +
    scale_fill_manual(
      name = "Nationalism",
      breaks = c("V. high", "V. low"),
      values = c("firebrick3", "forestgreen")
    ) +
    scale_shape_discrete(name = "Nationalism") +
    facet_grid(. ~ response, labeller=as_labeller(function(x) str_c("Rating: ", x))) +
    ylim(0, 1) +  
    theme_bw() + 
    theme(
      legend.position="right"
    )

figExp2Cultd.mean <- clm_plotdat(modelExp2AttitudesCult, c("spanishExpo", "diacritic"), xlevels=list(spanishExpo=c(2,7)), type="mean") %>%
  mutate(
    exposure = factor(spanishExpo, levels=c(7, 2), labels=c("High", "V. low")),
    diacritic = factor(diacritic, levels=c(TRUE, FALSE), labels=c("Present", "Absent"))
  ) %>%
  ggplot(., aes(x=diacritic, y=pred, color=exposure, shape=exposure)) +
    geom_point(size=4, position=position_dodge(width=0.2)) +
    geom_line(aes(x=as.integer(diacritic)), size=1, alpha=0.4, position=position_dodge(width=0.2)) +
    geom_errorbar(aes(ymin=lci, ymax=uci), size=1, width=0.3, position=position_dodge(width=0.2)) +
    xlab("Non-English characters") +
    ylab("Predicted mean rating") + 
    scale_color_manual(
      name = "Spanish\nexposure",
      breaks = c("High", "V. low"),
      values = c("forestgreen", "firebrick3")
      ) +
    scale_fill_manual(
      name = "Spanish\nexposure",
      breaks = c("High", "V. low"),
      values = c("forestgreen", "firebrick3")
    ) +
    scale_shape_discrete(name = "Spanish\nexposure") +
  ylim(1.8, 4.8) +
    theme_bw() + 
    theme(
      legend.position="none"
    )

figExp2Cultd.dist <- clm_plotdat(modelExp2AttitudesCult, c("spanishExpo", "diacritic"), xlevels=list(spanishExpo=c(2,7)), type="dist") %>%
  mutate(
    exposure = factor(spanishExpo, levels=c(7, 2), labels=c("High", "V. low")),
    diacritic = factor(diacritic, levels=c(TRUE, FALSE), labels=c("Present", "Absent"))
  ) %>%
  ggplot(., aes(x=diacritic, y=pred, color=exposure, shape=exposure)) +
    geom_point(size=2, position=position_dodge(width=0.2)) +
    geom_line(aes(x=as.integer(diacritic)), size=1, alpha=0.7, position=position_dodge(width=0.2)) +
    geom_errorbar(aes(ymin=lci, ymax=uci), size=1, width=0.3, position=position_dodge(width=0.2)) +
    xlab("Non-English characers") +
    ylab("Probability of rating") + 
    scale_color_manual(
      name = "Spanish\nexposure",
      breaks = c("High", "V. low"),
      values = c("forestgreen", "firebrick3")
      ) +
    scale_fill_manual(
      name = "Spanish\nexposure",
      breaks = c("High", "V. low"),
      values = c("forestgreen", "firebrick3")
    ) +
    scale_shape_discrete(name = "Spanish\nexposure") +
    facet_grid(. ~ response, labeller=as_labeller(function(x) str_c("Rating: ", x))) +
    ylim(0, 1) +  
    theme_bw() + 
    theme(
      legend.position="right"
    )

ggarrange(figExp2Culta.mean, figExp2Culta.dist, figExp2Cultb.mean, figExp2Cultb.dist, figExp2Cultc.mean, figExp2Cultc.dist, figExp2Cultd.mean, figExp2Cultd.dist, ncol=2, labels=c("a.", "", "b.", "", "C.", "", "d.", ""), widths=c(1,5))

remove(figExp2Culta.mean, figExp2Culta.dist, figExp2Cultb.mean, figExp2Cultb.dist, figExp2Cultc.mean, figExp2Cultc.dist, figExp2Cultd.mean, figExp2Cultd.dist)

remove(modelExp2AttitudesCult)
```

Partial effect plots for ordinal mixed-effects model of wellformedness ratings, as affected by perceived importance of Hispanic and Latino cultures. Left-hand plots show predicted mean ratings; right-hand plots show predicted distributions over ratings (facets). Top panel shows the interaction of phonotactic score with Spanish value (perceived cultural importance); remaining panels show the interaction between presence of non-English characters and Spanish value (perceived cultural importance), nationalism, and exposure to Spanish, respectively. Error bars represent 95% confidence intervals.

Together, these analyses show that the effects of Spanish value that are crucial for our interpretation are not driven by variation in positionalities toward Spanish language policy. If anything, it is possible that considering responses to language policy added noise to our original results, since there are many factors besides Spanish value that might affect someone’s opinion of language policy in education. Ultimately, these results are not surprising, given the exploratory factor analysis conducted in Appendix C, which showed that, while responses to both the language policy and the cultural importance question could be connected to the same underlying factor, the connection with the cultural importance question was stronger.
